# Supplementary material for: Accounting for interactions between Sustainable Development Goals is essential for water pollution control in China
Source: Nat Commun. 2022 Feb 8;13:730. doi: 10.1038/s41467-022-28351-3 (PMC8826988; doi:10.1038/s41467-022-28351-3)
Supplement: Supplementary file 1 — Supplementary Information [file 41467_2022_28351_MOESM1_ESM.pdf]

Supplementary Information (Appendixes)

Accounting for interactions between Sustainable Development Goals is essential for water pollution control in China

Mengru Wang[a,b,#], Annette B.G. Janssen[b], Jeanne Bazin[b], Maryna Strokal[b], Lin Ma[a,#], Carolien Kroeze[b]

[a] Key Laboratory of Agricultural Water Resources, Center for Agricultural Resources Research, Institute of Genetics and Developmental Biology, Chinese Academy of Sciences, 286 Huaizhong Road, Shijiazhuang 050021, China.

[b] Water Systems and Global Change Group, Wageningen University & Research, Droevendaalsesteeg 3, 6708 PB Wageningen, The Netherlands.

#Corresponding authors (Email: mengru.wang@wur.nl, malin1979@sjziam.ac.cn)

This supplementary information includes:

Appendix A. Nutrient pollution in Chinese water systems and the Sustainable Development Goals 2

Appendix B. Subbasins of the six rivers in China 66

Appendix C. Scenarios 68

Appendix D. Water quality standards in China and in other world regions 88

Appendix E. Sensitivity analysis 90

Supplementary References 99

Appendix A. Nutrient pollution in Chinese water systems and the Sustainable Development Goals

Supplementary Table 1 Sustainable development goals (SDGs) and the targets that are relevant to nutrient pollution in water systems in China. Three levels of the relevance of targets to nutrient pollution in water systems were identified– namely “High”, “Moderate” and “Low”. The level referred to as “High” relevance covers the targets that address the direct sources of nutrient losses to the Chinese water systems. The level referred to as “Moderate” relevance covers targets that address the impacts of nutrient pollution on aquatic ecosystems and human health, or influence the resilience of ecosystems to nutrient pollution. The level referred to as “Low” relevance covers targets based on technological, social, administrative or economic interventions indirectly related to nutrient pollution in water systems.

Goal	Targets relevant to nutrient pollution in water systems		Level of relevance	Explanations
SDG 6 & 14 on water systems				
**SDG 6 Clean water & Sanitation** - Ensure availability and sustainable management of water and sanitation for all.	6.1	By 2030, achieve universal and equitable access to safe and affordable drinking water for all.	High	Reducing nutrient pollution in shallow groundwater and surface water resources (e.g. from algal toxins) could help to achieve this target (Shen, 2006, Tao and Xin, 2014, Partnership, 2015, Zhang et al., 2010, Glibert, 2013).
	6.2	By 2030, achieve access to adequate and equitable sanitation and hygiene for all and end open defecation, paying special attention to the needs of women and girls and those in vulnerable situations.	High	Achieving this target could reduce nutrient pollution in water systems via ending open defecation and excreta disposal (Shen, 2006, Katukiza et al., 2012).
	6.3	By 2030, improve water quality by reducing pollution, eliminating dumping and minimizing release of hazardous chemicals and materials, halving the proportion of untreated wastewater and substantially increasing recycling and safe reuse globally.	High	Achieving this target could reduce nutrient pollution in water systems via decreasing (direct) wastewater discharges and improving wastewater treatment capacity, monitoring and maintenance which support Targets 6.1 and 6.2 (Abbasi et al., 2016, Partnership, 2015).

Goal	Targets relevant to nutrient pollution in water systems		Level of relevance	Explanations
**SDG 6 Clean water & Sanitation - Ensure availability and sustainable management of water and sanitation for all.**	6.4	By 2030, substantially increase water-use efficiency across all sectors and ensure sustainable withdrawals and supply of freshwater to address water scarcity and substantially reduce the number of people suffering from water scarcity.	High	Achieving this target could reduce nutrient pollution in water systems via improving water use efficiency in agriculture and upgrading the pipe networks of domestic wastewater that reduce nutrient leaching and runoff to waters (Partnership, 2015, Sun et al., 2012, Sutton et al., 2013, Brooks, 2015, Qin, 2015).
	6.5	By 2030, implement integrated water resources management at all levels, including through transboundary cooperation as appropriate.	Low	Achieving this target could reduce nutrient pollution in water systems via implementing integrated water resources management at all levels (e.g., controlling and monitoring water pollution) which supports Targets 6.14-6.4 (Partnership, 2015, Liu and Yang, 2012).
	6.6	By 2020, protect and restore water-related ecosystems, including mountains, forests, wetlands, rivers, aquifers and lakes.	Moderate	Achieving this target could reduce nutrient pollution in water systems via restoring natural functions of water-related ecosystems (e.g. nutrient retention mechanisms) which supports Targets 6.1 and 6.2 (Abbasi et al., 2016).
	6.A	By 2030, expand international cooperation and capacity-building support to developing countries in water and sanitation-related activities and programmes, including water harvesting, desalination, water efficiency, wastewater treatment, recycling and reuse technologies.	Low	Achieving this target could reduce nutrient pollution in water systems via enabling international cooperation on the use of rivers which supports Target 6.5 (Liu and Yang, 2012).

Goal	Targets relevant to nutrient pollution in water systems		Level of relevance	Explanations
**SDG 6 Clean water & Sanitation** - Ensure availability and sustainable management of water and sanitation for all.	6.B	Support and strengthen the participation of local communities in improving water and sanitation management.	Low	Achieving this target could reduce nutrient pollution in water systems via involving the local administrative units in developing water and sanitation management for reducing nutrient pollution which supports Target 6.5 (Murray et al., 2011).
**SDG 14 Life below water** - Conserve and sustainably use the oceans, seas and marine resources for sustainable development.	14.1	By 2025, prevent and significantly reduce marine pollution of all kinds, in particular from land-based activities, including marine debris and nutrient pollution.	High	Reducing river export of nutrients could help to achieve this target which supports Targets 14.2 and 14.3 (Chen et al., 2011, Zhang et al., 2008).
	14.2	By 2020, sustainably manage and protect marine and coastal ecosystems to avoid significant adverse impacts, including by strengthening their resilience, and take action for their restoration in order to achieve healthy and productive oceans.	Moderate	Achieving this target could reduce the impacts (e.g., eutrophication) of nutrient pollution in water systems via strengthening the resilience, and take action for restoration of the marine and coastal ecosystems (Zhang, 2016, Nyström et al., 2012).
	14.3	Minimize and address the impacts of ocean acidification, including through enhanced scientific cooperation at all levels.	Moderate	Reduce nutrient pollution and eutrophication could help to achieve this target as eutrophication is shown to enhance ocean acidification which supports Target 14.2 (Liu et al., 2014, Kelly et al., 2011, Zeng et al., 2015, Boyd, 2011).

Goal	Targets relevant to nutrient pollution in water systems		Level of relevance	Explanations
**SDG 14 Life below water** - Conserve and sustainably use the oceans, seas and marine resources for sustainable development.	14.4	By 2020, effectively regulate harvesting and end overfishing, illegal, unreported and unregulated fishing and destructive fishing practices and implement science-based management plans, in order to restore fish stocks in the shortest time feasible, at least to levels that can produce maximum sustainable yield as determined by their biological characteristics.	Moderate	Achieving this target could reduce the impacts (e.g., eutrophication) of nutrient pollution in water systems via preventing the collapse of fish communities as it results in adverse ecological consequences which supports Target 14.2 (Zhang, 2016).
	14.5	By 2020, conserve at least 10 per cent of coastal and marine areas, consistent with national and international law and based on the best available scientific information.	Low	Achieving this target could reduce nutrient pollution in marine and coastal ecosystems via the technological, administrative and social interventions which support Targets 14.1, 14.2 and 14.4.
	14.6	By 2020, prohibit certain forms of fisheries subsidies which contribute to overcapacity and overfishing, eliminate subsidies that contribute to illegal, unreported and unregulated fishing and refrain from introducing new such subsidies, recognizing that appropriate and effective special and differential treatment for developing and least developed countries should be an integral part of the World Trade Organization fisheries subsidies negotiation.	Low	Achieving this target could reduce nutrient pollution in marine and coastal ecosystems via the subsidies which support Target 14.4.

Goal	Targets relevant to nutrient pollution in water systems		Level of relevance	Explanations
**SDG 14 Life below water** - Conserve and sustainably use the oceans, seas and marine resources for sustainable development.	14.a	Increase scientific knowledge, develop research capacity and transfer marine technology, taking into account the Intergovernmental Oceanographic Commission Criteria and Guidelines on the Transfer of Marine Technology, in order to improve ocean health and to enhance the contribution of marine biodiversity to the development of developing countries, in particular small island developing States and least developed countries.	Low	Achieving this target could reduce nutrient pollution in marine and coastal ecosystems via the technological interventions which support Targets 14.1-3.
	14.c	Enhance the conservation and sustainable use of oceans and their resources by implementing international law as reflected in the United Nations Convention on the Law of the Sea, which provides the legal framework for the conservation and sustainable use of oceans and their resources, as recalled in paragraph 158 of "The future we want".	Low	Achieving this target could reduce nutrient pollution in marine and coastal ecosystems via the administrative interventions which support Targets 14.1-14.6.
Other SDGs				
**SDG 1 No poverty** - End poverty in all its forms everywhere	1.5	By 2030, build the resilience of the poor and those in vulnerable situations and reduce their exposure and vulnerability to climate-related extreme events and other economic, social and environmental shocks and disasters.	Moderate	Reducing nutrient pollution in water systems and its impacts on ecosystems (e.g. eutrophication) could help to achieve this target via providing a healthy environment and ecosystem services for reducing poverty (Zhen et al., 2014).

Goal	Targets relevant to nutrient pollution in water systems		Level of relevance	Explanations
**SDG 2 Zero hunger** - End hunger, achieve food security and improved nutrition and promote sustainable agriculture.	2.3	By 2030, double the agricultural productivity and incomes of small-scale food producers, in particular women, indigenous peoples, family farmers, pastoralists and fishers, including through secure and equal access to land, other productive resources and inputs, knowledge, financial services, markets and opportunities for value addition and non-farm employment.	High	Achieving this target could increase or reduce nutrient pollution in water systems from agriculture via increasing agricultural productivity, depending on the nutrient management in agriculture (e.g. fertilizer application, manure application) (Szogi et al., 2015, Li et al., 2013, Liu and Yang, 2012, Sutton et al., 2013, Smith and Siciliano, 2015, Brooks, 2015, Wang et al., 2018).
	2.4	By 2030, ensure sustainable food production systems and implement resilient agricultural practices that increase productivity and production, that help maintain ecosystems, that strengthen capacity for adaptation to climate change, extreme weather, drought, flooding and other disasters and that progressively improve land and soil quality.	High	Reducing nutrient losses in agriculture could help to achieve this target via mitigating the degradation of soil and water by providing guidance in fertilization practices, improving manure management and strengthening environmental laws and regulation for agriculture (Li et al., 2013, Sun et al., 2012, Smith and Siciliano, 2015, Sims et al., 2013, Wang et al., 2018).
	2.a	Increase investment, including through enhanced international cooperation, in rural infrastructure, agricultural research and extension services, technology development and plant and livestock gene banks in order to enhance agricultural productive capacity in developing countries, in particular least developed countries.	Low	Achieving this target could reduce nutrient pollution in water systems from agriculture via improving nutrient use efficiency by technological innovations in agriculture (e.g. animal breeding's that use nutrients more efficiently) which support Targets 2.3 and 2.4 (Li et al., 2013).

Goal	Targets relevant to nutrient pollution in water systems		Level of relevance	Explanations
**SDG 3 Good health & Well-being** - Ensure healthy lives and promote well-being for all at all ages.	3.9	By 2030, substantially reduce the number of deaths and illnesses from hazardous chemicals and air, water and soil pollution and contamination.	Moderate	Reducing nutrient pollution in water systems could help to achieve this target via reducing public health risk associated with nutrient pollution such as harmful algal blooms (HAB) (Zhang et al., 2010, Glibert, 2013)
**SDG 4 Quality education** - Ensure inclusive and equitable quality education and promote lifelong learning opportunities for all.	4.7	By 2030, ensure that all learners acquire the knowledge and skills needed to promote sustainable development, including, among others, through education for sustainable development and sustainable lifestyles, human rights, gender equality, promotion of a culture of peace and non-violence, global citizenship and appreciation of cultural diversity and of culture's contribution to sustainable development.	Low	Achieving this target could reduce nutrient pollution in water systems via improved education in sustainable agriculture at all levels (Li et al., 2013, Smith and Siciliano, 2015).
**SDG 5 Gender equality** - Achieve gender equality and empower all women and girls.	5.a	Undertake reforms to give women equal rights to economic resources, as well as access to ownership and control over land and other forms of property, financial services, inheritance and natural resources, in accordance with national laws.	Low	Achieving this target could reduce nutrient pollution in water systems via increasing the participation of women in environmental decision-making to improve water and agriculture management, and mitigate environmental degradation from nutrient pollution (Dankelman, 2012, Khosla et al., 2004).
**SDG 7 Affordable and clean energy** - Ensure access to affordable, reliable, sustainable and modern energy for all.	7.2	By 2030, increase substantially the share of renewable energy in the global energy mix.	Low	Achieving this target could reduce nutrient pollution in water systems via mitigating atmospheric N deposition by reducing reactive N emissions from fossil fuel burning (Zhu et al., 2015, Chen et al., 2011, Liu et al., 2013, Sutton et al., 2013).

Goal	Targets relevant to nutrient pollution in water systems		Level of relevance	Explanations
**SDG 7 Affordable and clean energy** - Ensure access to affordable, reliable, sustainable and modern energy for all.	7.3	By 2030, double the global rate of improvement in energy efficiency.	Low	Achieving this target could reduce nutrient pollution in water systems via mitigating atmospheric N deposition by improved energy efficiency (e.g., improving energy intensity, reducing energy consumption and ensuring technology development) (Zhu et al., 2015, Liu et al., 2015, Sutton et al., 2013).
	7.a	By 2030, enhance international cooperation to facilitate access to clean energy research and technology, including renewable energy, energy efficiency and advanced and cleaner fossil-fuel technology, and promote investment in energy infrastructure and clean energy technology.	Low	Achieving this target could reduce nutrient pollution in water systems via facilitating access to clean energy research and technology by the international society which supports Targets 7.2 and 7.3 (Gu et al., 2012b).
	7.b	By 2030, expand infrastructure and upgrade technology for supplying modern and sustainable energy services for all in developing countries, in particular least developed countries, small island developing States, and land-locked developing countries, in accordance with their respective programmes of support.	Low	Achieving this target could reduce nutrient pollution in water systems via the increased supply of modern and sustainable energy services by expanding infrastructure and upgrade technology, which supports Targets 7.2 and 7.3.
**SDG 8 Decent work & Economic growth** - Promote sustained, inclusive and sustainable economic growth, full and productive employment and decent work for all.	8.1	Sustain per capita economic growth in accordance with national circumstances and, in particular, at least 7 per cent gross domestic product growth per annum in the least developed countries.	Low	Achieving this target could reduce nutrient pollution in water systems via all means to support continued economic growth so nutrient pollution itself can be further tackled (Partnership, 2015, Sutton et al., 2013).

Goal	Targets relevant to nutrient pollution in water systems		Level of relevance	Explanations
**SDG 8 Decent work & Economic growth** - Promote sustained, inclusive and sustainable economic growth, full and productive employment and decent work for all.	8.4	Improve progressively, through 2030, global resource efficiency in consumption and production and endeavour to decouple economic growth from environmental degradation, in accordance with the 10-Year Framework of Programmes on Sustainable Consumption and Production, with developed countries taking the lead.	Low	Reducing nutrient pollution in water systems could help achieve this target via improved nutrient, water and energy efficiency at all levels that mitigate environmental degradation while support economic growth (Partnership, 2015, Liu et al., 2013).
**SDG 9 Industry, Innovation & Infrastructure** Build resilient infrastructure, promote inclusive and sustainable industrialization and foster innovation.	9.1	Develop quality, reliable, sustainable and resilient infrastructure, including regional and trans-border infrastructure, to support economic development and human well-being, with a focus on affordable and equitable access for all.	Low	Achieving this target may increase nutrient pollution in water systems via increasing reactive N emissions from fossil fuel combustion by developing the industrial sector and the transportation network (Gu et al., 2012a, Liu et al., 2013, Chen et al., 2011).
	9.3	Increase the access of small-scale industrial and other enterprises, in particular in developing countries, to financial services, including affordable credit, and their integration into value chains and markets.	Low	Achieving this target could reduce nutrient pollution in water systems via financial services that facilitate access to advanced technologies and wastewater treatment facilities to decrease nutrient pollution (Blanke et al., 2007)
	9.4	By 2030, upgrade infrastructure and retrofit industries to make them sustainable, with increased resource-use efficiency and greater adoption of clean and environmentally sound technologies and industrial processes, with all countries taking action in accordance with their respective capabilities.	Low	Achieving this target could reduce nutrient pollution in water systems via adopting energy-efficient systems, increasing wastewater recycling and upgrading infrastructures (Gu et al., 2012a, Sutton et al., 2013, Han et al., 2016).

Goal	Targets relevant to nutrient pollution in water systems		Level of relevance	Explanations
**SDG 9 Industry, Innovation & Infrastructure**<br><br>Build resilient infrastructure, promote inclusive and sustainable industrialization and foster innovation.	9.5	Enhance scientific research, upgrade the technological capabilities of industrial sectors in all countries, in particular developing countries, including, by 2030, encouraging innovation and substantially increasing the number of research and development workers per 1 million people and public and private research and development spending.	Low	Achieving this target could reduce nutrient pollution in water systems via enhanced research and novel technological capabilities which support Target 9.1 and 9.4.
**SDG 10 Reduced inequalities**<br>**-**<br>Reduce inequality within and among countries.	10.1	By 2030, progressively achieve and sustain income growth of the bottom 40 percent of the population at a rate higher than the national average.	Low	Achieving this target could reduce nutrient pollution in water systems via improving income of the poor to afford education, technology, recourses and infrastructure for sustainable lifestyles (Lu et al., 2019).
**SDG 11 Sustainable cities and communities**<br>-<br>Make cities and human settlements inclusive, safe, resilient and sustainable.	11.1	By 2030, ensure access for all to adequate, safe and affordable housing and basic services and upgrade slums.	High	Achieving this target could increase or reduce nutrient pollution in water systems via reduced open defecation by expanding the sewage network coverage, depending on the nutrient treatment efficiency of the sewage systems (Han et al., 2016, Shen, 2006, Gaffney, 2014).
	11.6	By 2030, reduce the adverse per capita environmental impact of cities, including by paying special attention to air quality and municipal and other waste management.	High	Achieving this target could reduce nutrient pollution in water systems via improved sewage management (e.g., expanding the sewage connection, improving sewage treatment, increasing wastewater reuse) in cities (Sutton et al., 2013, Partnership, 2015, Tao and Xin, 2014, Han et al., 2016).

Goal	Targets relevant to nutrient pollution in water systems		Level of relevance	Explanations
**SDG 12 Responsible consumption and production** - Ensure sustainable consumption and production patterns.	12.1	Implement the 10-Year Framework of Programmes on Sustainable Consumption and Production Patterns, all countries taking action, with developed countries taking the lead, taking into account the development and capabilities of developing countries.	Low	Achieving this target could reduce nutrient pollution in water systems via implementing the 10-Year Framework of Programmes which supports Targets 12.2 and 12.3.
	12.2	By 2030, achieve the sustainable management and efficient use of natural resources.	High	Achieving this target could reduce nutrient pollution in water systems via increasing water, fertilizer and energy efficiency (Partnership, 2015, Sun et al., 2012, Sutton et al., 2013, Brooks, 2015).
	12.3	By 2030, halve per capita global food waste at the retail and consumer levels and reduce food losses along production and supply chains, including post-harvest losses.	High	Achieving this target could reduce nutrient pollution in water systems via reduced food waste in food production, distribution and consumption (Sutton et al., 2013).
	12.5	By 2030, substantially reduce waste generation through prevention, reduction, recycling and reuse.	High	Achieving this target could reduce nutrient pollution in water systems via the prevention, reduction, recycling and reuse of waste (e.g., food residues) (Ma et al., 2019).
	12.8	By 2030, ensure that people everywhere have the relevant information and awareness for sustainable development and lifestyles in harmony with nature.	Low	Achieving this target could reduce nutrient pollution in water systems via enhanced environmental education to promote sustainable food consumption and production patterns which supports Targets 12.2 and 12.3 (Li et al., 2013, Smith and Siciliano, 2015, Chiu, 2011).

Goal	Targets relevant to nutrient pollution in water systems		Level of relevance	Explanations
**SDG 12 Responsible consumption and production** - Ensure sustainable consumption and production patterns.	12.a	Support developing countries to strengthen their scientific and technological capacity to move towards more sustainable patterns of consumption and production.	Low	Achieving this target could reduce nutrient pollution in water systems via strengthened scientific and technological capacity for more sustainable patterns of consumption and production which support Targets 12.1-12.3 and 12.5.
**SDG 13 Climate action** - Take urgent action to combat climate change and its impacts.	13.2	Integrate climate change measures into national policies, strategies and planning.	High	Achieving this target could reduce nutrient pollution in water systems via lowering the changes in climate parameters (e.g., runoff, river discharge, water temperature) that may cause increased nutrient runoff and leaching to water systems, or may increase the chances of eutrophication (Partnership, 2015, Alam and Dutta, 2013, Sutton et al., 2013, Zhang et al., 2010).
	13.3	Improve education, awareness-raising and human and institutional capacity on climate change mitigation, adaptation, impact reduction and early warning.	Low	Achieving this target could reduce nutrient pollution in water systems via improved education on climate change which supports Target 13.2.

Goal	Targets relevant to nutrient pollution in water systems		Level of relevance	Explanations
**SDG 15** **Life on land** - Protect, restore and promote sustainable use of terrestrial ecosystems, sustainably manage forests, combat desertification, and halt and reverse land degradation and halt biodiversity loss.	15.1	By 2020, ensure the conservation, restoration and sustainable use of terrestrial and inland freshwater ecosystems and their services, in particular forests, wetlands, mountains and drylands, in line with obligations under international agreements.	Moderate	Reducing nutrient pollution in water systems could help to achieve this target via mitigating degradation of freshwater ecosystems from nutrient pollution; Achieving this target could reduce nutrient pollution in water systems via conserving natural functions of land freshwater ecosystems (e.g. nutrient retention in land and waters) (Abbasi et al., 2016, Nyström et al., 2012, Gross and Hagy III, 2017, Partnership, 2015).
	15.2	By 2020, promote the implementation of sustainable management of all types of forests, halt deforestation, restore degraded forests and substantially increase afforestation and reforestation globally.	Moderate	Achieving this target could reduce nutrient pollution in water systems via conserving natural functions of forests (e.g. nutrient retention) (Abbasi et al., 2016, Nyström et al., 2012, Sutton et al., 2013).
	15.3	By 2030, combat desertification, restore degraded land and soil, including land affected by desertification, drought and floods, and strive to achieve a land degradation-neutral world.	Moderate	Achieving this target could reduce nutrient pollution via restoring degraded land and soil (e.g. nutrient retention) (Sutton et al., 2013, Zhu et al., 2015).
	15.a	Mobilize and significantly increase financial resources from all sources to conserve and sustainably use biodiversity and ecosystems.	Low	Achieving this target could reduce nutrient pollution in water systems via financial support to conserve ecosystems which supports Targets 15.1, 15.2 and 15.3.

Goal	Targets relevant to nutrient pollution in water systems		Level of relevance	Explanations
**SDG 16 Peace, Justice & Strong institutions** - Promote peaceful and inclusive societies for sustainable development, provide access to justice for all and build effective, accountable and inclusive institutions at all levels.	16.5	Substantially reduce corruption and bribery in all their forms.	Low	Achieving this target could reduce nutrient pollution in water systems via the implementation of laws and regulations for sustainable water management by suppressing conflicts of interests, shifting priorities and ensuring unbiased incentives (Brooks, 2015, Smith and Siciliano, 2015, Wang et al., 2008).
	16.6	Develop effective, accountable and transparent institutions at all levels.	Low	Achieving this target could reduce nutrient pollution in water systems via the implementation of laws and regulations for sustainable water management by strengthening authority in relation to the environment and increasing pressure for transparency and monitoring (Smith and Siciliano, 2015, Liu and Yang, 2012).
**SDG 17 Partnership for the Goals** - Strengthen the means of implementation and revitalize the Global Partnership for Sustainable Development.	17.16	Enhance the Global Partnership for Sustainable Development, complemented by multi-stakeholder partnerships that mobilize and share knowledge, expertise, technology and financial resources, to support the achievement of the Sustainable Development Goals.	Low	Achieving this target could reduce nutrient pollution in water systems via sharing of knowledge, expertise, technology and financial resources for water management with the enhanced global partnership for sustainable development (Liu and Yang, 2012, Sims et al., 2013).

Supplementary Table 2 The 'Seven-Point Scale' framework from Griggs et al. (2017) who classified the interactions at 7 levels: (-3) canceling, (-2) counteracting, (-1) constraining, (0) consistent, (+1) enabling, (+2) reinforcing, (+3) indivisible. Definitions of the seven levels are from Griggs et al. (2017). Examples are given in this table.

**Interactions**	**Synergies**			**Neutral**	**Trade-offs**		
**Scoring**	+3	+2	+1	0	-1	-2	-3
	Indivisible	Reinforcing	Enabling	Consistent	Constraining	Counteracting	Canceling
**Definition***	"The strongest form of positive interaction in which one objective is inextricably linked to the achievement of another."	"One objective directly creates conditions that lead to the achievement of another objective."	"The pursuit of one objective enables the achievement of another objective."	"A neutral relationship where one objective does not significantly interact with another or where interactions are deemed to be neither positive nor negative."	"A mild form of negative interaction when the pursuit of one objective sets a condition or a constraint on the achievement of another."	"The pursuit of one objective counteracts another objective."	"The most negative interaction is where progress in one goal makes it impossible to reach another goal and possibly leads to a deteriorating state of the second. A choice has to be made between the two."
**Example**	Achieving universal and equitable access to safe and affordable drinking water for all (6.1) is indivisible from substantially reducing death and illness from water pollution and contamination (3.9)	Agricultural practices (e.g., balanced fertilization) in sustainable food production system (2.4) reinforces improving water quality (6.3).	Enhance research that support technological capabilities (9.5) enables improve water quality (6.3) by facilitating the development of innovative clean technologies.	By 2020, sustainably manage and protect marine and coastal ecosystems (14.2) is consistent with developing quality, reliable, sustainable and resilient infrastructure, including regional and transborder infrastructure, to support economic development and human well-being (9.1)	(-1) Prohibiting certain forms of fisheries subsidies which contribute to overcapacity and overfishing (14.6) may constrain increasing productivity in aquaculture (2.3).	The reactive nitrogen emissions from energy use in the industrial sector and transportation network (9.1) may pollute water and thus counteract preventing and reducing marine pollution from land-based activities (14.1).	Prohibit certain forms of fisheries subsidies which contribute to overcapacity and overfishing (14.6) could lead to reduced income of the fishermen and thus canceling building the resilience of the poor and reducing their exposure and vulnerability to economic shock (1.5).

* Definitions are from Griggs et al. (2017)

Supplementary Table 3 Interactions between SDG 6 and other SDGs in China at the target level. Interactions between the targets that are identified relevant to nutrient pollution in Chinese water systems are analyzed. The interactions were assessed based on the 'Seven-Point Scale' framework from Griggs et al. (2017) who classified the interactions at 7 levels: (-3) canceling, (-2) counteracting, (-1) constraining, (0) consistent, (+1) enabling, (+2) reinforcing, (+3) indivisible. The definitions of the 7 levels of interactions are given in Supplementary Table 2.

			6 Clean Water and Sanitation							
			6.1	6.2	6.3	6.4	6.5	6.6	6.a	6.b
Goals	Targets		By 2030, achieve universal and equitable access to safe and affordable drinking water for all.	By 2030, achieve access to adequate and equitable sanitation and hygiene for all and end open defecation, paying special attention to the needs of women and girls and those in vulnerable situations.	By 2030, improve water quality by reducing pollution, eliminating dumping and minimizing release of hazardous chemicals and materials, halving the proportion of untreated wastewater and substantially increasing recycling and safe reuse globally.	By 2030, substantially increase water-use efficiency across all sectors and ensure sustainable withdrawals and supply of freshwater to address water scarcity and substantially reduce the number of people suffering from water scarcity.	By 2030, implement integrated water resources management at all levels, including through transboundary cooperation as appropriate.	By 2020, protect and restore water-related ecosystems, including mountains, forests, wetlands, rivers, aquifers and lakes.	By 2030, expand international cooperation and capacity-building support to developing countries in water- and sanitation-related activities and programmes, including water harvesting, desalination, water efficiency, wastewater treatment, recycling and reuse technologies.	Support and strengthen the participation of local communities in improving water and sanitation management.

			6 Clean Water and Sanitation							
			6.1	6.2	6.3	6.4	6.5	6.6	6.a	6.b
**1 No Poverty**	1.5	By 2030, build the resilience of the poor and those in vulnerable situations and reduce their exposure and vulnerability to climate-related extreme events and other economic, social and environmental shocks and disasters.	(+3) Achieving universal and equitable access to safe and affordable drinking water for all is indivisible from building the resilience of the poor and those in vulnerable situations.	(+3) Achieving access to adequate and equitable sanitation and hygiene for all is indivisible from building the resilience of the poor and those in vulnerable situations.	(+3) Improving water quality is indivisible from building the resilience of the poor and those in vulnerable situations caused by water pollution.	(+3) Building the resilience of the poor and those in vulnerable situations is indivisible from reducing water scarcity.	(+1) Implementing integrated water resources management at all levels enables building the resilience of the poor and those in vulnerable situations.	(+2) Protecting and restoring water-related ecosystems reinforces building the resilience of the poor and those in vulnerable situations.	(+1) International cooperation that supports sustainable water- and sanitation-related activities enable building the resilience of the poor and those in vulnerable situations.	(+1) Increasing participation of local communities in water and sanitation management enables building the resilience of the poor and those in vulnerable situations.
**2 Zero Hunger**	2.3	By 2030, double the agricultural productivity and incomes of small-scale food producers, in particular women, indigenous peoples, family farmers, pastoralists and fishers, including through secure and equal access to land, other productive resources and inputs, knowledge, financial services, markets and opportunities for value addition and non-farm employment.	(-2) Achieving universal and equitable access to safe and affordable drinking water for all can counteract adequate water supply for intensive agriculture, and vice versa.	(-2) The demand of water in sanitation and hygiene systems may counteract the adequate water supply for intensive agriculture, and vice versa.	(-2) Measures for improving water quality (e.g., limit fertilizer use) counteracts increasing agricultural productivity. (-3) Pollution in unsustainable agriculture can cancel the improvement of water quality.	(-3) Water use in intensive agriculture could cancel the reduction of water scarcity.	(-2) Integrated water management for transboundary rivers (e.g., Mekong river) can counteract improving agricultural productivity in some areas of the river basin (e.g., limited water for irrigation in the upstream area to maintain water supply for the downstream area).	(-2) Water use and pollution in intensive agriculture counteract protecting and restoring water-related ecosystems.		

			6 Clean Water and Sanitation							
			6.1	6.2	6.3	6.4	6.5	6.6	6.a	6.b
**2 Zero Hunger**	2.4	By 2030, ensure sustainable food production systems and implement resilient agricultural practices that increase productivity and production, that help maintain ecosystems, that strengthen capacity for adaptation to climate change, extreme weather, drought, flooding and other disasters and that progressively improve land and soil quality.			(+2) Agricultural practices (e.g., balanced fertilization) in sustainable food production system reinforces improving water quality.	(+2) Sustainable food production system that is efficient in water use reinforces improving water-use efficiency in the agricultural sector and reducing water scarcity, and vice versa.	(+1) Implementing integrated water recourses management at all levels enables implementing resilient agricultural practices.	(+2) Sustainable food production system reinforces protecting and restoring water-related ecosystems.		

			6 Clean Water and Sanitation							
			6.1	6.2	6.3	6.4	6.5	6.6	6.a	6.b
**2 Zero Hunger**	2.a	Increase investment, including through enhanced international cooperation, in rural infrastructure, agricultural research and extension services, technology development and plant and livestock gene banks in order to enhance agricultural productive capacity in developing countries, in particular least developed countries.			(+1) Infrastructure and technologies for enhancing agricultural productive capacity enable improving water quality by reducing pollution in agriculture (e.g., adopting livestock breed that uses nutrients more efficiently).					
**3 Good Health and Well-being**	3.9	By 2030, substantially reduce the number of deaths and illnesses from hazardous chemicals and air, water and soil pollution and contamination.	(+3) Achieving universal and equitable access to safe and affordable drinking water for all is indivisible from substantially reducing death and illness from water pollution and contamination.	(+3) Achieving access to adequate and equitable sanitation and hygiene for all is indivisible from substantially reducing death from water pollution and contamination.	(+3) Improving water quality is indivisible from substantially reducing death from water pollution and contamination.		(+1) Implement integrated water resources management at all levels enables reducing death from water pollution and contamination.	(+2) Protecting and restoring water-related ecosystems reinforces reducing death from water pollution and contamination.	(+1) International cooperation that supports sustainable water- and sanitation-related activities enable reducing death from water pollution and contamination.	(+1) Increased participation of local communities in water and sanitation management enables reducing death from water pollution and contamination.

			6 Clean Water and Sanitation							
			6.1	6.2	6.3	6.4	6.5	6.6	6.a	6.b
**4 Quality Education**	4.7	By 2030, ensure that all learners acquire the knowledge and skills needed to promote sustainable development, including, among others, through education for sustainable development and sustainable lifestyles, human rights, gender equality, promotion of a culture of peace and non-violence, global citizenship and appreciation of cultural diversity and of culture's contribution to sustainable development.	(+1) Acquire the knowledge and skills needed to promote sustainable development enables the achievement of SDG 6 targets.							
**5 Gender Equality**	5a	Undertake reforms to give women equal rights to economic resources, as well as access to ownership and control over land and other forms of property, financial services, inheritance and natural resources, in accordance with national laws.			(+1) Closing the gender gap enables improving water quality. Women are important labor forces in Chinese agriculture. Providing women access to resources, knowledge and services enables productive agriculture with lower pollution.	(+1) Closing the gender gap enables sustainable water withdraw. For example, providing women access to knowledge and services for sustainable water management enables productive agriculture with efficient water use.	(+1) Closing the gender gap enables integrated water resources management. For example, providing women access to knowledge and services for sustainable water management enables integrated water resources management in agriculture.	(+1) Closing the gender gap enables the protection and restoration of water-related ecosystems. For example, providing women access to knowledge and services for sustainable water management enables sustainable agriculture.		

			6 Clean Water and Sanitation							
			6.1	6.2	6.3	6.4	6.5	6.6	6.a	6.b
**7 Affordable and Clean Energy**	7.2	By 2030, increase substantially the share of renewable energy in the global energy mix.	(+2/-2) Increasing the share of renewable energy reinforce/counteract achieve universal and equitable access to safe and affordable drinking water for all, and vice versa. For example, building multipurpose hydropower reservoirs helps to provide drinking water meanwhile producing renewable energy. However, when reservoirs in upstream take too much water for other purposes, there is a threat to adequate drinking water in downstream.		(+2) Increased use of renewable energy may reduce the nitrogen emissions from energy use and thus reinforces reducing water pollution from atmospheric nitrogen deposition.	(+2/-2) Increasing the share of renewable energy could reinforce/counteract improving water-use efficiency and reducing water scarcity. For example, building multipurpose hydropower reservoirs helps to increase water use efficiency while producing renewable energy. However, when reservoirs in upstream take too much water for other purposes, there is a risk of water scarcity in downstream.		(+2) Increased use of renewable energy may counteract protecting and restoring water-related ecosystems through energy production (e.g., hydropower).		
	7.3	By 2030, double the global rate of improvement in energy efficiency.			(+2) Increased energy use efficiency may reduce the nitrogen emissions from energy use and thus reinforces reducing water pollution from atmospheric nitrogen deposition.					

			6 Clean Water and Sanitation							
			6.1	6.2	6.3	6.4	6.5	6.6	6.a	6.b
**7 Affordable and Clean Energy**	7.a	By 2030, enhance international cooperation to facilitate access to clean energy research and technology, including renewable energy, energy efficiency and advanced and cleaner fossil-fuel technology, and promote investment in energy infrastructure and clean energy technology.			(+1) International cooperation that facilities clean energy research and technology may reduce the nitrogen emissions from energy use and thus enables reducing water pollution from atmospheric nitrogen deposition.					
	7.b	By 2030, expand infrastructure and upgrade technology for supplying modern and sustainable energy services for all in developing countries, in particular least developed countries, small island developing States, and land-locked developing countries, in accordance with their respective programmes of support.			(+1) Expanded infrastructure and upgrade technology for modern and sustainable energy services may reduce the nitrogen emissions from energy use and thus enables reducing water pollution from atmospheric nitrogen deposition.		(+1) Integrated water management enables expanding infrastructure for sustainable energy services, for example, by hydropower reservoirs.			

			6 Clean Water and Sanitation							
			6.1	6.2	6.3	6.4	6.5	6.6	6.a	6.b
**8 Decent Work and Economic Growth**	8.1	Sustain per capita economic growth in accordance with national circumstances and, in particular, at least 7 per cent gross domestic product growth per annum in the least developed countries.	(+1) Economic growth enables the achievement of SDG 6 targets for example by supporting the development of technologies and infrastructure, implementation of laws and regulations.							
	8.4	Improve progressively, through 2030, global resource efficiency in consumption and production and endeavour to decouple economic growth from environmental degradation, in accordance with the 1-year framework of programmes on sustainable consumption and production, with developed countries taking the lead.	(+1) Improving resource efficiency in consumption and production (e.g., water use efficiency in all sectors) enables universal and equitable access to safe and affordable drinking water for all.		(+1) Improve resource efficiency in consumption and production (e.g., nutrients) enables improve water quality.		(+2) Improving resource (e.g., water) efficiency in consumption and production reinforces the implementation of integrated water resources management at all levels.	(+1) Improve resource(e.g., nutrients) efficiency in consumption and production enables preventing environmental degradation of water-related ecosystems.		

			6 Clean Water and Sanitation							
			6.1	6.2	6.3	6.4	6.5	6.6	6.a	6.b
**9 Industry, innovation and Infrastructure**	9.1	Develop quality, reliable, sustainable and resilient infrastructure, including regional and transborder infrastructure, to support economic development and human well-being, with a focus on affordable and equitable access for all.			(-2) The reactive nitrogen emissions from energy use in the industrial sector and transportation network may pollute water and thus counteract improve water quality.					
	9.3	Increase the access of small-scale industrial and other enterprises, in particular in developing countries, to financial services, including affordable credit, and their integration into value chains and markets.			(+1) Access to financial services may facilitate access to clean technologies/services that enable improving water quality.	(+1) Access to financial services may facilitate access to clean technologies/services that enable improving water-use efficiency.				

			6 Clean Water and Sanitation							
			6.1	6.2	6.3	6.4	6.5	6.6	6.a	6.b
**9 Industry, innovation and Infrastructure**	9.4	By 2030, upgrade infrastructure and retrofit industries to make them sustainable, with increased resource-use efficiency and greater adoption of clean and environmentally sound technologies and industrial processes, with all countries taking action in accordance with their respective capabilities.	(+2) Upgrade infrastructure and retrofit industries to make them sustainable reinforce universal and equitable access to safe and affordable drinking water for all by reducing polluted water (e.g., water contains various chemicals, warm water from thermal pollution) from industries affecting drinking water.		(+3) Increase resource-use efficiency and adoption of clean technologies for industries and infrastructure is indivisible from improving water quality.	(+2) Upgrade infrastructure and retrofit industries to make them sustainable with increase water-use efficiency reinforce reducing water scarcity.	(+1) Implementing integrated water resources management enables increasing resource-use efficiency and adoption of clean technologies for industries and infrastructures.	(+1) Sustainable infrastructure and industries enable preventing environmental degradation and its impact on water-related ecosystems.		
	9.5	Enhance scientific research, upgrade the technological capabilities of industrial sectors in all countries, in particular developing countries, including, by 2030, encouraging innovation and substantially increasing the number of research and development workers per 1 million people and public and private research and development spending.			(+1) Enhance research that support technological capabilities enables improving water quality by facilitating the development of innovative clean technologies.	(+1) Enhance research that support technological capabilities in industrial sectors enables improving water use efficiency and sustainable withdrawals of freshwater by facilitating the development of innovative clean technologies.		(+1) Enhance research that support technological capabilities in industrial sectors enables limiting environmental degradation of water-related ecosystems by facilitating the development of innovative clean technologies.		

			6 Clean Water and Sanitation							
			6.1	6.2	6.3	6.4	6.5	6.6	6.a	6.b
**10 Reduced Inequalities**	10.1	By 2030, progressively achieve and sustain income growth of the bottom 40 per cent of the population at a rate higher than the national average.	(+1) Income growth of the poor enables the achievement of SDG 6 targets by supporting the poor with more access to knowledge, technologies, infrastructure and services for sustainable water management.							
**11 Sustainable Cities and Communities**	11.1	By 2030, ensure access for all to adequate, safe and affordable housing and basic services and upgrade slums.	(+3) Achieving universal and equitable access to safe and affordable drinking water for all is indivisible from ensuring access to adequate, safe and affordable housing and basic services.	(+3) Achieving access to adequate and equitable sanitation and hygiene for all is indivisible from ensuring access to adequate, safe and affordable housing and basic services.	(+2/-2) Access to adequate, safe and affordable housing and basic services (e.g. sanitation) reinforces or counteracts improving water quality, and vice versa, depending on the pollution management in cities (e.g., wastewater treatment).	(-2) Ensuring access to adequate, safe and affordable housing and basic services could counteract reducing water scarcity as adequate housed people generally use water less efficient, e.g. by bathing/showering often and long.		(-2) Ensuring access to adequate, safe and affordable housing and basic services could counteract protecting and restoring water-related ecosystems due to loss of land/water area as the result of housing.		
	11.6	By 2030, reduce the adverse per capita environmental impact of cities, including by paying special attention to air quality and municipal and other waste management.			(+3) Reducing water pollution in cities by improving wastewater management is indivisible improving water quality, and vice versa.	(+2) Improving water-use efficiency and sustainable withdrawals and supply of freshwater in cities reinforces reducing the adverse environmental impact of cities.	(+1) Implementing integrated water resources management in cities enables reducing the adverse environmental impact of cities.	(+2) Reducing the adverse environmental impact of cities reinforces the protection of water-related ecosystems.		

			6 Clean Water and Sanitation							
			6.1	6.2	6.3	6.4	6.5	6.6	6.a	6.b
**12 Responsible Consumption and Production**	12.1	Implement the 10-year framework of programmes on sustainable consumption and production, all countries taking action, with developed countries taking the lead, taking into account the development and capabilities of developing countries.			(+1) Implement programmes on sustainable consumption and production enable improving water quality by reducing water pollution from for example food production and consumption.	(+1) Implement programmes on sustainable consumption and production enable improving water use efficiency and sustainable withdrawals and supply of freshwater.	(+1) Implement programmes on sustainable consumption and production enable implementing integrated water resources management at all levels.	(+1) Implement programmes on sustainable consumption and production enable the protection of water-related ecosystems by limiting environmental degradation.		
	12.2	By 2030, achieve the sustainable management and efficient use of natural resources.	(+3) Sustainable management and efficient use of surface and groundwater resources are indivisible from achieving universal and equitable access to safe and affordable drinking water.		(+2) Sustainable management and efficient use of natural resources (e.g., nutrients, water) reinforce improving water quality by reducing pollution, and vice versa.	(+2) Increasing water-use efficiency reinforces sustainable management of efficient use of natural resources, and vice versa.	(+3) Sustainable management and efficient use of surface and groundwater resources are indivisible from improving water use efficiency and sustainable water withdraws and supply, and vice versa.	(+2) Achieve the sustainable management and efficient use of natural resources reinforces the protection of water-related ecosystems.		
	12.3	By 2030, halve per capita global food waste at the retail and consumer levels and reduce food losses along production and supply chains, including post-harvest losses.			(+2) Reduce food waste in the food production-consumption chain reinforces improving water quality.					

			6 Clean Water and Sanitation							
			6.1	6.2	6.3	6.4	6.5	6.6	6.a	6.b
**12 Responsible Consumption and Production**	12.5	By 2030, substantially reduce waste generation through prevention, reduction, recycling and reuse.	(+2) Reducing water waste from consumption reinforces achieving universal and equitable access to safe and affordable drinking water.		(+2) Reduce food waste in the food production-consumption chain reinforces improving water quality.	(+2) Improving water-use efficiency and sustainable withdrawals and supply of freshwater reinforces reducing waste from water consumption and food production through prevention, reduction, recycling and reuse, and vice versa.	(+1) Implementing integrated water resources management enables reducing waste from water consumption.	(+1) Reduce waste generation may reduce adverse environmental impact related to production and consumption and thus enables the protection of water-related ecosystems.		
	12.8	By 2030, ensure that people everywhere have the relevant information and awareness for sustainable development and lifestyles in harmony with nature.	(+1) Information and awareness for sustainable development by people enables sustainable water consumption to ensure safe and adequate drinking water for all.		(+1) Information and awareness for sustainable development by people enables improving water quality in relevant sectors.	(+1) Information and awareness for sustainable development by people enables increasing water use efficiency in relevant sectors.		(+1) Information and awareness for sustainable development by people enables protecting water-related ecosystems.		
	12.a	Support developing countries to strengthen their scientific and technological capacity to move towards more sustainable patterns of consumption and production.	(+1) The scientific and technological support for more sustainable patterns of consumption and production enables ensuring safe and adequate drinking water for all.		(+1) The scientific and technological support for more sustainable patterns of consumption and production enables improving water quality in relevant sectors.	(+1) The scientific and technological support for more sustainable patterns of consumption and production enables improving water use efficiency and sustainable withdrawal and supply.		(+1) The scientific and technological support for more sustainable patterns of consumption and production enables protecting water-related ecosystems.		

			6 Clean Water and Sanitation							
			6.1	6.2	6.3	6.4	6.5	6.6	6.a	6.b
**13 Climate Action**	13.2	Integrate climate change measures into national policies, strategies and planning.	(+2) Integrating climate change measures into national policies, strategies and planning reinforces reducing impacts of climate extremes on water (e.g., droughts, flooding), thus enables ensuring safe and adequate drinking water for all.		(+2) Integrating climate change measures into national policies, strategies and planning reinforce improving water quality in the long term, as climate change induced changes in hydrology (e.g., surface runoff) may increase water pollution by nutrients in the future.			(+1) Integrating climate change measures into national policies, strategies and planning enable protecting the water-related ecosystems, as climate change induces changes in climate extremes (e.g., flood, drought) affect the ecosystems.		
	13.3	Improve education, awareness-raising and human and institutional capacity on climate change mitigation, adaptation, impact reduction and early warning.	(+1) Improving education, awareness-raising and human and institutional capacity on climate change mitigation, adaptation, impact reduction and early warning enables reducing impacts of climate extremes on water (e.g., droughts, flooding), thus reinforces ensuring safe and adequate drinking water for all.		(+1) Improving education, awareness-raising and human and institutional capacity on climate change mitigation, adaptation, impact reduction and early warning enables improving water quality in the long term, as climate change induced changes in hydrology (e.g., surface runoff) may increase water pollution by nutrients in the future.			(+1) Improving education, awareness-raising and human and institutional capacity on climate change mitigation, adaptation, impact reduction and early warning enables protecting the water-related ecosystems, as climate change induces changes in climate extremes (e.g., flood, drought) affect the ecosystems.		

			6 Clean Water and Sanitation							
			6.1	6.2	6.3	6.4	6.5	6.6	6.a	6.b
**14 Life Below Water**	14.1	By 2025, prevent and significantly reduce marine pollution of all kinds, in particular from land-based activities, including marine debris and nutrient pollution.		(+2/-2) Achieving access to adequate sanitation for all reinforces or constraints preventing and reducing marine pollution from human waste, depending on the sewage treatment efficiencies.	(+3) Improving water quality by reducing pollution is indivisible from preventing and significantly reducing marine pollution; (+2) Measures for preventing and reducing marine pollution from land-based activities (e.g., agriculture, urbanization) reinforces improving water quality by reducing pollution.		(+1) Implement integrated water resources management at all levels enables preventing and reducing marine pollution from all kinds, in particular from land-based activities.	(+1) Protecting and restoring water-related ecosystems enables preventing and reducing marine pollution via the ecosystem functions (e.g., nutrient retention by the water-related ecosystems).	(+1) International cooperation that supports sustainable water- and sanitation-related activities enable preventing and reducing marine pollution from all kinds, in particular from land-based activities.	(+1) Increased participation of local communities in water and sanitation management enables preventing and reducing marine pollution from human waste.
	14.2	By 2020, sustainably manage and protect marine and coastal ecosystems to avoid significant adverse impacts, including by strengthening their resilience, and take action for their restoration in order to achieve healthy and productive oceans.						(+2) Protecting and restoring inland water-related ecosystems reinforces sustainably managing and protecting marine and coastal ecosystems.		
	14.3	Minimize and address the impacts of ocean acidification, including through enhanced scientific cooperation at all levels.						(+2) Protecting and restoring inland water-related ecosystems reinforces minimizing and addressing the impacts of ocean acidification.		

			6 Clean Water and Sanitation							
			6.1	6.2	6.3	6.4	6.5	6.6	6.a	6.b
**14 Life Below Water**	14.4	By 2020, effectively regulate harvesting and end overfishing, illegal, unreported and unregulated fishing and destructive fishing practices and implement science-based management plans, in order to restore fish stocks in the shortest time feasible, at least to levels that can produce maximum sustainable yield as determined by their biological characteristics.								
	14.5	By 2020, conserve at least 10 per cent of coastal and marine areas, consistent with national and international law and based on the best available scientific information.						(+1) Conserving marine ecosystems enables conserving other water-related ecosystems that are degraded, and vice versa.		

			6 Clean Water and Sanitation							
			6.1	6.2	6.3	6.4	6.5	6.6	6.a	6.b
**14 Life Below Water**	14.6	By 2020, prohibit certain forms of fisheries subsidies which contribute to overcapacity and overfishing, eliminate subsidies that contribute to illegal, unreported and unregulated fishing and refrain from introducing new such subsidies, recognizing that appropriate and effective special and differential treatment for developing and least developed countries should be an integral part of the World Trade Organization fisheries subsidies negotiation.								

			6 Clean Water and Sanitation							
			6.1	6.2	6.3	6.4	6.5	6.6	6.a	6.b
**14 Life Below Water**	14.a	Increase scientific knowledge, develop research capacity and transfer marine technology, taking into account the Intergovernmental Oceanographic Commission Criteria and Guidelines on the Transfer of Marine Technology, in order to improve ocean health and to enhance the contribution of marine biodiversity to the development of developing countries, in particular small island developing States and least developed countries.								

			6 Clean Water and Sanitation							
			6.1	6.2	6.3	6.4	6.5	6.6	6.a	6.b
**14 Life Below Water**	14.c	Enhance the conservation and sustainable use of oceans and their resources by implementing international law as reflected in UNCLOS, which provides the legal framework for the conservation and sustainable use of oceans and their resources, as recalled in paragraph 158 of The Future We Want.								

			6 Clean Water and Sanitation							
			6.1	6.2	6.3	6.4	6.5	6.6	6.a	6.b
**15 Life on Land**	15.1	By 2020, ensure the conservation, restoration and sustainable use of terrestrial and inland freshwater ecosystems and their services, in particular forests, wetlands, mountains and drylands, in line with obligations under international agreements.	(-2) Achieving universal and equitable access to safe and affordable drinking water for all in regions with water scarcity counteracts the conservation, restoration and sustainable use of terrestrial and inland freshwater ecosystem (demand of drinking water vs. demand for water for nature), and vice versa.		(+3) The conservation, restoration and sustainable use of terrestrial and inland freshwater ecosystems is indivisible from improving water quality; (-3) Measures for reducing water pollution (e.g., nutrient pollution from agriculture) may cause higher emissions of pollutants to the air (e.g., ammonia emissions), land and water (e.g., through atmospheric N deposition) and canceling the effects of measures for ensuring the conservation and restoration of terrestrial and inland freshwater ecosystems.	(+2) Improving the water use efficiency and sustainable withdrawal and supply of freshwater reinforces the conservation and sustainable use of terrestrial and inland freshwater ecosystems.	(+2) Integrated water management reinforces the conservation, restoration and sustainable use of the inland terrestrial and freshwater ecosystem.	(+3) Protecting and restoring water-related ecosystems is indivisible from the conservation, restoration and sustainable use of terrestrial and inland freshwater ecosystems, and vice versa.		
	15.2	By 2020, promote the implementation of sustainable management of all types of forests, halt deforestation, restore degraded forests and substantially increase afforestation and reforestation globally.			(+2) Sustainable management of forests reinforces improving water quality through ecosystem functions of forest (e.g., nutrient retention by forest).			(+2) Sustainable management of forests reinforces protecting and restoring water-related ecosystems, and vice versa.		

			6 Clean Water and Sanitation							
			6.1	6.2	6.3	6.4	6.5	6.6	6.a	6.b
**15 Life on Land**	15.3	By 2030, combat desertification, restore degraded land and soil, including land affected by desertification, drought and floods, and strive to achieve a land degradation-neutral world.	(+1) Combating desertification, restoring degraded land and soil affected by desertification, droughts and floods enable ecosystems to provide more drinking water.		(+2) Restoring degraded land and soil reinforces improving water quality through ecosystem functions of land (e.g., nutrient retention by soil).		(+2) Integrated water management reinforces combatin g desertification, restoring degraded land and soil affected by desertification, drought and floods.	(+2) Restoring degraded land and soil reinforces protecting and restoring water-related ecosystems, and vice versa.		
	15.a	Mobilize and significantly increase financial resources from all sources to conserve and sustainably use biodiversity and ecosystems.			(+1) Financial support for conserving and sustainably using biodiversity and ecosystems enables improve water quality through ecosystem functions (e.g., nutrient retention by the land and aquatic ecosystems).			(+2) Financial support for conserving and sustainably using biodiversity and ecosystems reinforces protecting and restoring water-related ecosystems.		
**16 Peace, Justice and Strong Institutions**	16.5	Substantially reduce corruption and bribery in all their form.	(+1) Reduce corruption and bribery in all their form enables the achievement of SDG 6 targets by suppressing conflicts of interests, shifting priorities and ensuring unbiased incentives.							
	16.6	Develop effective, accountable and transparent institutions at all levels.	(+1) Develop effective, accountable and transparent institutions at all levels enables the achievement of SDG 6 targets by strengthening authority in relation to the environment and increasing pressure for transparency and monitoring of policies, laws and regulations.							

			6 Clean Water and Sanitation							
			6.1	6.2	6.3	6.4	6.5	6.6	6.a	6.b
**17 Partnerships**	17.16	Enhance the global partnership for sustainable development, complemented by multi-stakeholder partnerships that mobilize and share knowledge, expertise, technology and financial resources, to support the achievement of the sustainable development goals in all countries, in particular developing countries.	(+1) Enhance the global partnership for sustainable development enables the achievement of SDG 6 targets by the sharing of knowledge, expertise, technology and financial resources for water management.							

Supplementary Table 4 Interactions between SDG 14 and other SDGs in China at the target level. Interactions between the targets that are identified relevant to nutrient pollution in Chinese water systems are analyzed. The interactions were assessed based on the 'Seven-Point Scale' framework from Griggs et al. (2017) who classified the interactions at 7 levels: (-3) canceling, (-2) counteracting, (-1) constraining, (0) consistent, (+1) enabling, (+2) reinforcing, (+3) indivisible. The definitions of the 7 levels of interactions are given in Supplementary Table 2.

			14 Life Below Water							
			14.1	14.2	14.3	14.4	14.5	14.6	14.a	14.c
SDGs	Targets		By 2025, prevent and significantly reduce marine pollution of all kinds, in particular from land-based activities, including marine debris and nutrient pollution.	By 2020, sustainably manage and protect marine and coastal ecosystems to avoid significant adverse impacts, including by strengthening their resilience, and take action for their restoration in order to achieve healthy and productive oceans.	Minimize and address the impacts of ocean acidification, including through enhanced scientific cooperation at all levels.	By 2020, effectively regulate harvesting and end overfishing, illegal, unreported and unregulated fishing and destructive fishing practices and implement science-based management plans, in order to restore fish stocks in the shortest time feasible, at least to levels that can produce maximum sustainable yield as determined by their biological characteristics.	By 2020, conserve at least 10 per cent of coastal and marine areas, consistent with national and international law and based on the best available scientific information.	By 2020, prohibit certain forms of fisheries subsidies which contribute to overcapacity and overfishing, eliminate subsidies that contribute to illegal, unreported and unregulated fishing and refrain from introducing new such subsidies, recognizing that appropriate and effective special and differential treatment for developing and least developed countries should be an integral part of the World Trade Organization fisheries subsidies negotiation.	Increase scientific knowledge, develop research capacity and transfer marine technology, taking into account the Intergovernmental Oceanographic Commission Criteria and Guidelines on the Transfer of Marine Technology, in order to improve ocean health and to enhance the contribution of marine biodiversity to the development of developing countries, in particular small island developing States and least developed countries.	Enhance the conservation and sustainable use of oceans and their resources by implementing international law as reflected in UNCLOS, which provides the legal framework for the conservation and sustainable use of oceans and their resources, as recalled in paragraph 158 of The Future We Want.

			14 Life Below Water							
			14.1	14.2	14.3	14.4	14.5	14.6	14.a	14.c
**1 No Poverty**	1.5	By 2030, build the resilience of the poor and those in vulnerable situations and reduce their exposure and vulnerability to climate-related extreme events and other economic, social and environmental shocks and disasters.	(+3) Preventing and reducing marine pollution is indivisible from building the resilience of the poor and those in vulnerable situations in the coastal regions.	(+2) Sustainably managing and protecting marine and coastal ecosystems reinforce building the resilience of the poor and those in vulnerable situations in the coastal regions.	(+2) Minimizing and address the impacts of ocean acidification reinforces building the resilience of the poor an those in vulnerable situations in the coastal regions.		(+1) Conserving coastal and marine areas based on laws and scientific information enables building the resilience of the poor and those in vulnerable situations in the coastal regions.	(-3) Prohibit certain forms of fisheries subsidies could lead to reduced income of the fishermen and thus canceling building the resilience of the poor and reducing their exposure and vulnerability to an economic shock.	(+1) The scientific support to improve ocean health enables building the resilience of the poor and those in vulnerable situations in the coastal regions.	(+1) International law that enhances the conservation and sustainable use of oceans enables building the resilience of the poor and those in vulnerable situations in the coastal regions.
**2 Zero Hunger**	2.3	By 2030, double the agricultural productivity and incomes of small-scale food producers, in particular women, indigenous peoples, family farmers, pastoralists and fishers, including through secure and equal access to land, other productive resources and inputs, knowledge, financial services, markets and opportunities for value addition and non-farm employment.	(-2) Measures for preventing marine pollution from land-based activities and aquaculture (e.g., limit fertilizer use in agriculture) can counteract increasing agricultural productivity. (-3) Pollution in unsustainable agriculture (e.g., industrialized animal production with poor manure management) can cancel preventing and reducing marine pollution.	(-2) Measures for protecting marine and coastal ecosystems from land-based activities (e.g., limit fertilizer use in agriculture) can counteract increasing agricultural productivity. (-3) Pollution in unsustainable agriculture (e.g., industrialized animal production with poor manure management) can cancel the restoration of the marine and coastal ecosystems.	(-2) Measures for minimizing and addressing the impacts of ocean acidification (e.g., reduce nutrient pollution in aquaculture) can counteract increasing agricultural productivity. Pollution in unsustainable agriculture (e.g., industrialized animal production with poor manure management) can counteract the restoration of the marine and coastal ecosystems.	(-2) Regulating harvesting and ending overfishing can counteract increasing productivity of aquaculture. Overfishing in aquaculture to improve agricultural productivity can counteract regulating harvesting and ending overfishing.	(-1) Measures for conserving coastal and marine areas may constrain increasing agricultural productivity. (-2) Pollution in unsustainable agriculture (e.g., nutrient pollution in aquaculture, overfishing) can counteract conserving coastal and marine areas.	(-1) Prohibiting certain forms of fisheries subsidies may constrain increasing productivity in aquaculture.		

			14 Life Below Water							
			14.1	14.2	14.3	14.4	14.5	14.6	14.a	14.c
**2 Zero Hunger**	2.4	By 2030, ensure sustainable food production systems and implement resilient agricultural practices that increase productivity and production, that help maintain ecosystems, that strengthen capacity for adaptation to climate change, extreme weather, drought, flooding and other disasters and that progressively improve land and soil quality.	(+2) Measures for preventing marine pollution from land-based activities (e.g., limit fertilizer use in agriculture) reinforces sustainable food production systems and resilient agricultural practices, and vice versa.	(+2) Measures for protecting marine and coastal ecosystems from land-based activities (e.g., limit fertilizer use in agriculture) reinforces sustainable food production systems and implementing resilient agricultural practices, and vice versa.	(+2) Measures for minimizing and addressing the impacts of ocean acidification (e.g., reduce nutrient pollution in aquaculture) reinforces sustainable food production systems and implementing resilient agricultural practices, and vice versa.	(+2) Regulating harvesting and ending overfishing reinforces sustainable food production systems and implementing resilient agricultural practices, and vice versa.	(+1) Ensuring sustainable food production systems and implementing resilient agricultural practices enables conserving coastal and marine areas.	(+2) Prohibiting certain forms of fisheries subsidies that contribute to overcapacity and overfishing reinforces sustainable food production systems and implementing resilient agricultural practices.		

			14 Life Below Water							
			14.1	14.2	14.3	14.4	14.5	14.6	14.a	14.c
**2 Zero Hunger**	2.a	Increase investment, including through enhanced international cooperation, in rural infrastructure, agricultural research and extension services, technology development and plant and livestock gene banks in order to enhance agricultural productive capacity in developing countries, in particular least developed countries.	(+1) Infrastructure and technologies for enhancing agricultural productive capacity enables reducing marine pollution from land-based agricultural activities (e.g., adopting livestock breed that uses nutrients more efficiently).							
**3 Good Health and Well-being**	3.9	By 2030, substantially reduce the number of deaths and illnesses from hazardous chemicals and air, water and soil pollution and contamination.	(+3) Preventing and reducing marine pollution is indivisible from substantially reducing death and illness from water pollution and contamination.	(+2) Sustainably managing and protecting marine and coastal ecosystems reinforces substantially reducing death and illness from water pollution and contamination.	(+2) Minimizing and address the impacts of ocean acidification reinforces substantially reducing death and illness from water pollution and contamination.		(+1) Conserving coastal and marine areas based on laws and scientific information enables conserving the marine ecosystems, thus reducing death and illness from coastal water pollution and contamination.			

			14 Life Below Water							
			14.1	14.1	14.1	14.1	14.1	14.1	14.1	14.1
**4 Quality Education**	4.7	By 2030, ensure that all learners acquire the knowledge and skills needed to promote sustainable development, including, among others, through education for sustainable development and sustainable lifestyles, human rights, gender equality, promotion of a culture of peace and non-violence, global citizenship and appreciation of cultural diversity and of culture's contribution to sustainable development.	(+1) Acquire the knowledge and skills needed to promote sustainable development enables the achievement of SDG 14 targets.							
**5 Gender Equality**	5a	Undertake reforms to give women equal rights to economic resources, as well as access to ownership and control over land and other forms of property, financial services, inheritance and natural resources, in accordance with national laws.	(+1) Closing the gender gap enables preventing and reducing marine pollution from human activities. For example, providing women access to resources, knowledge and services enables productive agriculture with lower water pollution.			(+1) Closing the gender gap enables effectively regulating harvesting and ending overfishing. For example, providing women access to resources, knowledge and services enables sustainable fishing.				

			14 Life Below Water							
			14.1	14.2	14.3	14.4	14.5	14.6	14.a	14.c
**6 Clean Water and Sanitation**	6.1	By 2030, achieve universal and equitable access to safe and affordable drinking water for all.								
	6.2	By 2030, achieve access to adequate and equitable sanitation and hygiene for all and end open defecation, paying special attention to the needs of women and girls and those in vulnerable situations.	(+2/-2) Achieving access to adequate sanitation for all reinforces or counteracts preventing and reducing marine pollution from human waste, depending on the sewage treatment efficiencies.							
	6.3	By 2030, improve water quality by reducing pollution, eliminating dumping and minimizing release of hazardous chemicals and materials, halving the proportion of untreated wastewater and substantially increasing recycling and safe reuse globally.	(+3) Improving water quality by reducing pollution is indivisible from preventing and significantly reducing marine pollution; (+2) Measures for preventing and reducing marine pollution from land-based activities (e.g., agriculture, urbanization) reinforces improving water quality by reducing pollution.							

			14 Life Below Water							
			14.1	14.2	14.3	14.4	14.5	14.6	14.a	14.c
**6 Clean Water and Sanitation**	6.4	By 2030, substantially increase water-use efficiency across all sectors and ensure sustainable withdrawals and supply of freshwater to address water scarcity and substantially reduce the number of people suffering from water scarcity.								
	6.5	By 2030, implement integrated water resources management at all levels, including through transboundary cooperation as appropriate.	(+1) Implement integrated water resources management at all levels enables preventing and reducing marine pollution from all kinds, in particular from land-based activities.							
	6.6	By 2020, protect and restore water-related ecosystems, including mountains, forests, wetlands, rivers, aquifers and lakes.	(+2) Protecting and restoring water-related ecosystems reinforces preventing and reducing marine pollution via the ecosystem functions (e.g., nutrient retention by the water-related ecosystems).	(+2) Protecting and restoring inland water-related ecosystems reinforces sustainably managing and protecting marine and coastal ecosystems.	(+2) Protecting and restoring inland water-related ecosystems reinforces minimizing and addressing the impacts of ocean acidification.		(+1) Conserving marine ecosystems enables conserving other water-related ecosystems that are degraded, and vice versa.			

			14 Life Below Water							
			14.1	14.2	14.3	14.4	14.5	14.6	14.a	14.c
	6.a	By 2030, expand international cooperation and capacity-building support to developing countries in water- and sanitation-related activities and programmes, including water harvesting, desalination, water efficiency, wastewater treatment, recycling and reuse technologies.	(+1) International cooperation that supports sustainable water- and sanitation-related activities enables preventing and reducing marine pollution from all kinds, in particular from land-based activities.							
	6.b	Support and strengthen the participation of local communities in improving water and sanitation management.	(+1) Increased participation of local communities in water and sanitation management enables preventing and reducing marine pollution from human waste.							

			14 Life Below Water							
			14.1	14.2	14.3	14.4	14.5	14.6	14.a	14.c
**7 Affordable and Clean Energy**	7.2	By 2030, increase substantially the share of renewable energy in the global energy mix.	(+2) Increased use of renewable energy may reduce the nitrogen emissions from energy use and thus reinforces reducing marine pollution from atmospheric nitrogen deposition.	(+2/-2) Production of renewable energy reinforces or counteracts sustainably managing and protecting marine and coastal ecosystems. For example, offshore wind energy can negatively influence marine animals and birds, thus damage marine ecosystems. The production of the same wind energy, on the other hand, does not emit environmental pollutants or greenhouse gases, which is good for protecting the marine ecosystems from pollution.	(+2) Increased use of renewable energy may reduce the nitrogen emissions from energy use and thus reinforces mitigating ocean acidification enhanced by atmospheric nitrogen deposition.		(+1) Increased use of renewable energy may reduce the nitrogen emissions from energy use and thus enables conserving marine and coastal areas by limiting adverse environmental impacts of unsustainable energy use.			
	7.3	By 2030, double the global rate of improvement in energy efficiency.	(+2) Increased energy use efficiency may reduce the nitrogen emissions from energy use and thus reinforces reducing marine pollution from atmospheric nitrogen deposition.		(+2) Increased energy use efficiency may reduce the nitrogen emissions from energy use and thus reinforces mitigating ocean acidification enhanced by atmospheric nitrogen deposition.		(+1) Increased energy use efficiency may reduce the nitrogen emissions from energy use and thus enables conserving marine and coastal areas by limiting adverse environmental impacts of unsustainable energy use.			

			14 Life Below Water							
			14.1	14.2	14.3	14.4	14.5	14.6	14.a	14.c
**7 Affordable and Clean Energy**	7.a	By 2030, enhance international cooperation to facilitate access to clean energy research and technology, including renewable energy, energy efficiency and advanced and cleaner fossil-fuel technology, and promote investment in energy infrastructure and clean energy technology.	(+1) International cooperation that facilities clean energy research and technology may reduce the nitrogen emissions from energy use and thus enables reducing marine pollution from atmospheric nitrogen deposition.		(+1) International cooperation that facilities clean energy research and technology may reduce the nitrogen emissions from energy use and thus enables mitigating ocean acidification, enhanced by atmospheric nitrogen deposition.		(+1) International cooperation that facilities clean energy research and technology may reduce the nitrogen emissions from energy use and thus enables conserving marine and coastal areas by limiting adverse environmental impacts of unsustainable energy production.			
	7.b	By 2030, expand infrastructure and upgrade technology for supplying modern and sustainable energy services for all in developing countries, in particular least developed countries, small island developing States, and land-locked developing countries, in accordance with their respective programmes of support.	(+1) Sustainable energy services may reduce the nitrogen emissions from energy use and thus enables reducing marine pollution from atmospheric nitrogen deposition.		(+1) Sustainable energy services may reduce the nitrogen emissions from energy use and thus enables mitigating ocean acidification, enhanced by atmospheric nitrogen deposition.		(+1) Sustainable energy services may reduce the nitrogen emissions from energy use and enables conserving marine and coastal areas by limiting adverse environmental impacts of unsustainable energy production and supply.			

			14 Life Below Water							
			14.1	14.2	14.3	14.4	14.5	14.6	14.a	14.c
**8 Decent Work and Economic Growth**	8.1	Sustain per capita economic growth in accordance with national circumstances and, in particular, at least 7 per cent gross domestic product growth per annum in the least developed countries.	(+1) Economic growth enables the achievement of SDG 14 targets by supporting the development of technologies and infrastructure, implementation of laws and regulations.							
	8.4	Improve progressively, through 2030, global resource efficiency in consumption and production and endeavour to decouple economic growth from environmental degradation, in accordance with the 10-year framework of programmes on sustainable consumption and production, with developed countries taking the lead.	(+1) Improve resource efficiency in consumption and production (e.g., nutrients) enables reducing marine pollution from resource consumption and production.			(+2) Improving resource efficiency in consumption and production and endeavour to decouple economic growth from environmental degradation reinforces effectively regulating harvesting and ending overfishing in the marine ecosystems, and vice versa.				

			14 Life Below Water							
			14.1	14.2	14.3	14.4	14.5	14.6	14.a	14.c
**9 Industry, innovation and Infrastructure**	9.1	Develop quality, reliable, sustainable and resilient infrastructure, including regional and transborder infrastructure, to support economic development and human well-being, with a focus on affordable and equitable access for all.	(-2) The reactive nitrogen emissions from energy use in the industrial sector and transportation network may pollute water and thus counteract preventing and reducing marine pollution from land-based activities.		(-2) The reactive carbon emissions from energy use in the industrial sector and transportation network may counteract mitigating ocean acidification.		(-1) The reactive nitrogen emissions from energy use in the industrial sector and transportation network may pollute water and the development of marine traffic thus constrain conserving marine and coastal areas.			
	9.3	Increase the access of small-scale industrial and other enterprises, in particular in developing countries, to financial services, including affordable credit, and their integration into value chains and markets.	(+1) Access to financial services may facilitate access to clean technologies/services that enables preventing and reducing marine pollution from land-based activities.		(+1) Access to financial services may facilitate access to clean technologies/services that enables mitigating ocean acidification by reducing carbon emissions from industrial activities.		(+1) Access to financial services may facilitate access to clean technologies/services that enables preventing and reducing marine pollution from industrial activities.			

			14 Life Below Water							
			14.1	14.2	14.3	14.4	14.5	14.6	14.a	14.c
**9 Industry, innovation and Infrastructure**	9.4	By 2030, upgrade infrastructure and retrofit industries to make them sustainable, with increased resource-use efficiency and greater adoption of clean and environmentally sound technologies and industrial processes, with all countries taking action in accordance with their respective capabilities.	(+2) Increase resource-use efficiency and adoption of clean technologies for industries and infrastructure reinforces preventing and reducing marine pollution from land-based activities.		(+2) Increase resource-use efficiency and adoption of clean technologies for industries and infrastructure may reduce carbon emissions from industries and thus reinforces mitigating ocean acidification.		(+1) Increase resource-use efficiency and adoption of clean technologies for industries and infrastructure enables conserving marine and coastal areas.			

			14 Life Below Water							
			14.1	14.2	14.3	14.4	14.5	14.6	14.a	14.c
**9 Industry, innovation and Infrastructure**	9.5	Enhance scientific research, upgrade the technological capabilities of industrial sectors in all countries, in particular developing countries, including, by 2030, encouraging innovation and substantially increasing the number of research and development workers per 1 million people and public and private research and development spending.	(+1) Enhance research that support technological capabilities enables preventing and reducing marine pollution by facilitating the development of innovative clean technologies.		(+1) Enhance research that supports technological capabilities may reduce carbon emissions from industries and thus reinforces mitigating ocean acidification.					
**10 Reduced Inequalities**	10.1	By 2030, progressively achieve and sustain income growth of the bottom 40 per cent of the population at a rate higher than the national average.	(+1) Income growth of the poor enables the achievement of SDG 14 targets by supporting the poor with more access to knowledge , technologies, infrastructure and services for sustainable marine management.							

			14 Life Below Water							
			14.1	14.2	14.3	14.4	14.5	14.6	14.a	14.c
**11 Sustainable Cities and Communities**	11.1	By 2030, ensure access for all to adequate, safe and affordable housing and basic services and upgrade slums.	(+2/-2) Access to adequate, safe and affordable housing and basic services (e.g. sanitation) reinforces or counteracts preventing and reducing marine pollution from human activities, and vice versa, depending on the pollution management in cities (e.g., wastewater treatment).							
	11.6	By 2030, reduce the adverse per capita environmental impact of cities, including by paying special attention to air quality and municipal and other waste management.	(+2) Preventing and reducing marine pollution from land-based activities reinforces reducing water pollution in cities by improving wastewater management, and vice versa.				(+1) Reducing the adverse environmental impact of cities in deltas enables conserving marine and coastal areas.			
**12 Responsible Consumption and Production**	12.1	Implement the 10-year framework of programmes on sustainable consumption and production, all countries taking action, with developed countries taking the lead, taking into account the development and capabilities of developing countries.	(+1) Implement programmes on sustainable consumption and production enables preventing and reducing marine pollution by reducing water pollution from for example food production and consumption.		(+1) Implement programmes on sustainable consumption and production enables mitigating ocean acidification resulting from human activities, involved with for example food production and consumption.	(+1) Implement programmes on sustainable consumption and production enables regulating harvesting and ending overfishing.	(+1) Implement programmes on sustainable consumption and production may prevent and reduce marine pollution and other adverse environmental impacts of human activities, and thus enables protecting marine and coastal ecosystems.			(+1) Implement programmes on sustainable consumption and production enables enhancing the conservation and sustainable use of oceans and their resources by implementing laws, and vice versa.

			14 Life Below Water							
			14.1	14.2	14.3	14.4	14.5	14.6	14.a	14.c
**12 Responsible Consumption and Production**	12.2	By 2030, achieve the sustainable management and efficient use of natural resources.	(+2) Sustainable management and efficient use of natural resources (e.g., nutrient management in aquaculture) reinforces preventing and reducing marine pollution from all kinds by reducing pollution, and vice versa.	(+2) Achieving the sustainable management and efficient use of natural resources (e.g., fish) reinforces sustainably manage and protect marine and coastal ecosystems to avoid significant adverse impacts.		(+2) Effectively regulating harvesting and ending overfishing in the marine ecosystems reinforces achieving sustainable management and efficient use of natural resources (e.g., fish), and vice versa.				(+1) Enhancing the conservation and sustainable use of oceans and their resources (e.g., fish) by implementing laws enables achieving sustainable management and efficient use of natural resources.
	12.3	By 2030, halve per capita global food waste at the retail and consumer levels and reduce food losses along production and supply chains, including post-harvest losses.	(+2) Reducing food waste in the food production-consumption chain reinforces preventing and reducing marine pollution from food waste.			(+2) Reducing food waste in the food production-consumption chain reduces the demand for food (e.g., fish), thus reinforces regulating harvesting and ending overfishing in the marine ecosystems.	(+1) Reducing food waste and its adverse environmental impacts enables conserving marine and coastal areas.			
	12.5	By 2030, substantially reduce waste generation through prevention, reduction, recycling and reuse.	(+2) Reducing food waste in the food production-consumption chain reinforces preventing and reducing marine pollution from food waste.				(+1) Reducing food waste and its adverse environmental impacts enables conserving marine and coastal areas.			

			14 Life Below Water							
			14.1	14.2	14.3	14.4	14.5	14.6	14.a	14.c
**12 Responsible Consumption and Production**	12.8	By 2030, ensure that people everywhere have the relevant information and awareness for sustainable development and lifestyles in harmony with nature.	(+1) Information and awareness for sustainable development by people enables preventing and reducing marine pollution from human activities.	(+1) Information and awareness for sustainable development and harmony with nature by people enables sustainably manage and protect marine and coastal ecosystems to avoid significant adverse impacts.	(+1) Information and awareness for sustainable development and harmony with nature by people enables mitigating ocean acidification resulting from human activities.	(+1) Information and awareness for sustainable development and harmony with nature by people enables regulating harvesting and ending overfishing in the marine ecosystems.	(+1) Information and awareness for sustainable development and harmony with nature by people enables conserving marine and coastal areas.			
	12.a	Support developing countries to strengthen their scientific and technological capacity to move towards more sustainable patterns of consumption and production.	(+1) The scientific and technological support for more sustainable patterns of consumption and production enables, preventing and reducing marine pollution from human activities.		(+1) The scientific and technological support for more sustainable patterns of consumption and production enables mitigating ocean acidification resulting from human activities.	(+1) The scientific and technological support for more sustainable patterns of fish consumption and production enables regulating harvesting and ending overfishing in the marine ecosystems.	(+1) The scientific and technological support for more sustainable patterns of consumption and production enables conserving marine and coastal areas.			
**13 Climate Action**	13.2	Integrate climate change measures into national policies, strategies and planning.	(+2) Integrate climate change measures into national policies, strategies and planning reinforces preventing and reducing marine pollution in the long term, as climate change induced changes in hydrology (e.g., surface runoff) may increase water pollution by nutrients in the future.	(+1) Integrate climate change measures into national policies, strategies and planning enables protecting marine and coastal ecosystems in the long term.	(+2) Integrate climate change measures into national policies, strategies and planning reinforces preventing and reducing marine pollution in the long term, as climate change induced changes (e.g., water temperature) may increase ocean acidification in the future.		(+1) Integrate climate change measures into national policies, strategies and planning enables conserving marine and coastal areas in the long term.			

			14 Life Below Water							
			14.1	14.2	14.3	14.4	14.5	14.6	14.a	14.c
**13 Climate Action**	13.3	Improve education, awareness-raising and human and institutional capacity on climate change mitigation, adaptation, impact reduction and early warning.	(+1) Improving education, awareness-raising and human and institutional capacity on climate change mitigation, adaptation, impact reduction and early warning enables preventing and reducing marine pollution in the long term, as climate change induced changes in hydrology (e.g., surface runoff) may increase water pollution by nutrients in the future.	(+1) Improving education, awareness-raising and human and institutional capacity on climate change mitigation, adaptation, impact reduction and early warning enables protecting marine and coastal ecosystems in the long term.	(+1) Improving education, awareness-raising and human and institutional capacity on climate change mitigation, adaptation, impact reduction and early warning enables preventing and reducing marine pollution in the long term, as climate change induced changes (e.g., water temperature) may increase ocean acidification in the future.		(+1) Improving education, awareness-raising and human and institutional capacity on climate change mitigation, adaptation, impact reduction and early warning enables conserving marine and coastal areas in the long term.			
**15 Life on Land**	15.1	By 2020, ensure the conservation, restoration and sustainable use of terrestrial and inland freshwater ecosystems and their services, in particular forests, wetlands, mountains and drylands, in line with obligations under international agreements.	(+3) Preventing and reducing marine pollution is indivisible from the conservation, restoration and sustainable use of terrestrial and inland freshwater ecosystems, and vice versa.	(+2) Conserving the function of terrestrial and inland freshwater ecosystems reinforces sustainably manage and protect marine and coastal ecosystems to avoid significant adverse impacts. For example, marine fish species (such as salmon and eel) that spawn in fresh water are highly dependent on the inland freshwater ecosystems.			(+2) Conserving the function of terrestrial and inland freshwater ecosystems reinforces conserving marine and coastal areas.			

			14 Life Below Water							
			14.1	14.2	14.3	14.4	14.5	14.6	14.a	14.c
**15 Life on Land**	15.2	By 2020, promote the implementation of sustainable management of all types of forests, halt deforestation, restore degraded forests and substantially increase afforestation and reforestation globally.	(+2) Sustainable management of forests reinforces preventing and reducing marine pollution from land-based activities through ecosystem functions of forest (e.g., nutrient retention by forest).		(+1) Sustainable management of forests may enable mitigating ocean acidification through carbon sequestration by trees.					
	15.3	By 2030, combat desertification, restore degraded land and soil, including land affected by desertification, drought and floods, and strive to achieve a land degradation-neutral world.	(+2) Restoring degraded land and soil reinforces preventing and reducing marine pollution from land-based activities through ecosystem functions of land (e.g., nutrient retention by soil).							
	15.a	Mobilize and significantly increase financial resources from all sources to conserve and sustainably use biodiversity and ecosystems.	(+1) Financial support for conserving and sustainably using biodiversity and ecosystems enables preventing and reducing marine pollution from land-based activities through ecosystem functions (e.g., nutrient retention by the land and aquatic ecosystems).	(+1) Financial support for conserving and sustainably using biodiversity and ecosystems enables sustainably manage and protect marine and coastal ecosystems.		(+1) Financial support for conserving and sustainably using biodiversity and ecosystems enables regulating harvesting and ending overfishing.	(+1) Financial support for conserving and sustainably using biodiversity and ecosystems enables conserving 10% of the coastal and marine areas.			

			14 Life Below Water							
			14.1	14.2	14.3	14.4	14.5	14.6	14.a	14.c
**16 Peace, Justice and Strong Institutions**	16.5	Substantially reduce corruption and bribery in all their form.	(+1) Reduce corruption and bribery in all their form enables the achievement of SDG 14 targets by suppressing conflicts of interests, shifting priorities and ensuring unbiased incentives.							
	16.6	Develop effective, accountable and transparent institutions at all levels.	(+1) Develop effective, accountable and transparent institutions at all levels enables the achievement of SDG 14 targets by strengthening authority in relation to the environment and increasing pressure for transparency and monitoring of policies, laws and regulations.							
**17 Partnerships**	17.16	Enhance the global partnership for sustainable development, complemented by multi-stakeholder partnerships that mobilize and share knowledge, expertise, technology and financial resources, to support the achievement of the sustainable development goals in all countries, in particular developing countries.	(+1) Enhance the global partnership for sustainable development enables the achievement of SDG 14 targets by the sharing of knowledge, expertise, technology and financial resources for water management.							

Supplementary Table 5 Sustainable development goals (SDGs) and targets. The keywords of the targets are indicated in bold. These keywords are opposed to different keywords related to nutrient pollution such as “nutrient pollution”, “nutrient management”, or else “water quality” to span the array of academic literature potentially existing on the subject of nutrient pollution in water systems. Additional keywords such as “China” or “Chinese waters” were added to the query, so the literature review was made specific to the national or regional context. The resulted literature are shown in Supplementary Table 1.

Goals and targets	Keywords
Goal 1. End poverty in all its forms everywhere	
1.1	By 2030, **eradicate extreme poverty** for all people everywhere, currently measured as people living on less than $1.25 a day
1.2	By 2030, **reduce** at least by half the proportion of men, women and children of **all ages living in poverty** in all its dimensions according to national definitions
1.3	**Implement** nationally appropriate **social protection systems** and **measures** for all, including floors, and by 2030 achieve substantial coverage of the poor and the vulnerable
1.4	By 2030, **ensure** that all men and women, in particular the poor and the vulnerable, have **equal rights to economic resources**, as well as access to **basic services**, **ownership and control over land and other forms of property, inheritance, natural resources,** appropriate new **technology and financial services**, including microfinance
1.5	By 2030, **build** the **resilience** of the poor and those **in vulnerable situations** and **reduce** their **exposure and vulnerability to climate-related extreme events and other economic, social and environmental shocks and disasters**
1.a	**Ensure** significant **mobilization of resources** from a variety of sources, including through enhanced development cooperation, in order to provide adequate and predictable means for developing countries, in particular least developed countries, to implement programmes and policies to end poverty in all its dimensions
1.b	**Create sound policy frameworks** at the national, regional and international levels, based on pro-poor and gender-sensitive development strategies, to support accelerated **investment in poverty eradication actions**
Goal 2. End hunger, achieve food security and improved nutrition and promote sustainable agriculture	
2.1	By 2030, **end hunger** and ensure access by all people, in particular the poor and people in vulnerable situations, including infants, to **safe, nutritious and sufficient food** all year round
2.2	By 2030, **end** all forms of **malnutrition**, including achieving, by 2025, the internationally agreed targets on stunting and wasting in children under 5 years of age, and address the nutritional needs of adolescent girls, pregnant and lactating women and older persons
2.3	By 2030, **double the agricultural productivity and incomes of small-scale food producers**, in particular women, indigenous peoples, family farmers, pastoralists and fishers, including through secure and equal access to land, other productive resources and inputs, knowledge, financial services, markets and opportunities for value addition and non-farm employment
2.4	By 2030, **ensure sustainable food production systems** and **implement resilient agricultural practices** that increase productivity and production, that help maintain ecosystems, that strengthen capacity for adaptation to climate change, extreme weather, drought, flooding and other disasters and that progressively improve land and soil quality
2.5	By 2020, **maintain the genetic diversity of seeds, cultivated plants and farmed and domesticated animals and their related wild species,** including through soundly managed and diversified seed and plant banks at the national, regional and international levels, and promote access to and fair and equitable sharing of benefits arising from the utilization of genetic resources and associated traditional knowledge, as internationally agreed
2.a	**Increase investment**, including through enhanced **international cooperation**, **in rural infrastructure, agricultural research and extension services, technology development and plant and livestock gene banks** in order to enhance agricultural productive capacity in developing countries, in particular least developed countries
2.b	**Correct and prevent trade restrictions and distortions in world agricultural markets**, including through the parallel elimination of all forms of agricultural export subsidies and all export measures with equivalent effect, in accordance with the mandate of the Doha Development Round
2.c	Adopt measures to **ensure the proper functioning of food commodity markets** and their **derivatives** and **facilitate** timely **access to market information**, including on food reserves, in order to help limit extreme food price volatility
Goal 3. Ensure healthy lives and promote well-being for all at all ages	
3.1	By 2030, **reduce** the global **maternal mortality ratio** to less than 70 per 100,000 live births
3.2	By 2030, **end preventable deaths of newborns and children** under 5 years of age, with all countries aiming to reduce neonatal mortality to at least as low as 12 per 1,000 live births and under-5 mortality to at least as low as 25 per 1,000 live births
3.3	By 2030, **end the epidemics** of AIDS, tuberculosis, malaria and neglected tropical diseases and combat hepatitis, water-borne diseases and other communicable diseases
3.4	By 2030, **reduce by one third premature mortality from non-communicable diseases** through prevention and treatment and promote mental health and well-being

Goals and targets	Keywords
3.5	**Strengthen the prevention and treatment of substance abuse**, including narcotic drug abuse and harmful use of alcohol
3.6	By 2020, **halve** the number of global **deaths and injuries** from **road traffic accidents**
3.7	By 2030, **ensure universal access to sexual and reproductive health-care services**, including for family planning, information and education, and the integration of reproductive health into national strategies and programmes
3.8	**Achieve universal health coverage**, including financial risk protection, access to quality essential health-care services and access to safe, effective, quality and affordable essential medicines and vaccines for all
3.9	By 2030, substantially **reduce** the number of **deaths and illnesses** from **hazardous chemicals and air, water and soil pollution and contamination**
3.a	**Strengthen** the implementation of the World Health Organization Framework Convention on **Tobacco Control** in all countries, as appropriate
3.b	Support the **research and development** of **vaccines and medicines** for the communicable and non-communicable diseases that primarily affect developing countries, provide access to **affordable essential medicines and vaccines**, in accordance with the Doha Declaration on the TRIPS Agreement and Public Health, which affirms the right of developing countries to use to the full the provisions in the Agreement on Trade-Related Aspects of Intellectual Property Rights regarding flexibilities to protect public health, and, in particular, provide access to medicines for all
3.c	Substantially **increase health financing and the recruitment, development, training and retention of the health workforce** in developing countries, especially in least developed countries and small island developing States
3.d	**Strengthen the capacity** of all countries, in particular developing countries, **for early warning, risk reduction and management of** national and global **health risks**
Goal 4. Ensure inclusive and equitable quality education and promote lifelong learning opportunities for all	
4.1	By 2030, **ensure** that all girls and boys **complete free, equitable** and **quality primary and secondary education** leading to relevant and effective learning outcomes
4.2	By 2030, **ensure** that all girls and boys have **access to quality early childhood development**, **care and pre-primary education** so that they are ready for primary education
4.3	By 2030, **ensure equal access** for all women and men to **affordable and quality technical, vocational and tertiary education**, including university
4.4	By 2030, substantially **increase the number** of youth and adults **who have relevant skills**, including technical and vocational skills, for employment, decent jobs and entrepreneurship
4.5	By 2030, **eliminate gender disparities in education** and **ensure equal access to** all levels of **education and vocational training** for the **vulnerable**, including persons with disabilities, indigenous peoples and children in vulnerable situations
4.6	By 2030, **ensure** that all youth and a substantial proportion of adults, both men and women, **achieve literacy and numeracy**
4.7	By 2030, **ensure** that all **learners acquire the knowledge and skills needed to promote sustainable development**, including, among others, through education for sustainable development and sustainable lifestyles, human rights, gender equality, promotion of a culture of peace and non-violence, global citizenship and appreciation of cultural diversity and of culture's contribution to sustainable development
4.a	**Build and upgrade education facilities** that are child, disability and gender sensitive and provide safe, non-violent, inclusive and effective learning environments for all
4.b	By 2020, substantially **expand** globally the number of **scholarships available to developing countries**, in particular least developed countries, small island developing States and African countries, for enrolment in higher education, including vocational training and information and communications technology, technical, engineering and scientific programmes, in developed countries and other developing countries
4.c	By 2030, substantially **increase the supply of qualified teachers**, including through international cooperation for teacher training in developing countries, especially least developed countries and small island developing States

Goals and targets	Keywords
Goal 5. Achieve gender equality and empower all women and girls	
5.1	**End** all forms of **discrimination against all women and girls** everywhere
5.2	**Eliminate** all forms of **violence against all women and girls** in the public and private spheres, including trafficking and sexual and other types of exploitation
5.3	**Eliminate all harmful practices**, such as child, early and forced marriage and female genital mutilation
5.4	**Recognize and value unpaid care and domestic work** through the provision of public services, infrastructure and social protection policies and the promotion of shared responsibility within the household and the family as nationally appropriate
5.5	**Ensure women's full and effective participation and equal opportunities for leadership** at all levels of decisionmaking in political, economic and public life
5.6	**Ensure universal access to sexual and reproductive health and reproductive rights** as agreed in accordance with the Programme of Action of the International Conference on Population and Development and the Beijing Platform for Action and the outcome documents of their review conferences
5.a	**Undertake reforms to give women equal rights to economic resources**, as well as access to **ownership and control over land and other forms of property, financial services, inheritance and natural resources,** in accordance with national laws
5.b	**Enhance the use of enabling technology**, in particular information and communications technology, to promote the empowerment of women
5.c	**Adopt and strengthen sound policies** and **enforceable legislation** for the **promotion of gender equality** and the **empowerment of all women and girls** at all levels
Goal 6. Ensure availability and sustainable management of water and sanitation for all	
6.1	By 2030, **achieve** universal and equitable **access to safe and affordable drinking wate**r for all
6.2	By 2030, **achieve access** to adequate and equitable **sanitation and hygiene** for all and **end open defecation**, paying special attention to the needs of women and girls and those in vulnerable situations
6.3	By 2030, **improve water quality by reducing pollution**, **eliminating dumping and minimizing release of hazardous chemicals and materials**, **halving** the proportion of **untreated wastewater** and substantially **increasing recycling and safe reuse** globally
6.4	By 2030, substantially **increase water-use efficiency** across all sectors and **ensure sustainable withdrawals and supply of freshwater** to address water scarcity and substantially reduce the number of people suffering from water scarcity
6.5	By 2030, **implement integrated water resources management** at all levels, including through transboundary cooperation as appropriate
6.6	By 2020, **protect and restore water-related ecosystems**, including mountains, forests, wetlands, rivers, aquifers and lakes
6.a	By 2030, **expand international cooperation and capacity-building support** to developing countries **in water and sanitation-related activities and programmes**, including water harvesting, desalination, water efficiency, wastewater treatment, recycling and reuse technologies
6.b	**Support** and strengthen the participation of **local communities in improving water and sanitation management**
Goal 7. Ensure access to affordable, reliable, sustainable and modern energy for all	
7.1	By 2030, **ensure** universal **access** to affordable, reliable and modern **energy services**
7.2	By 2030, **increase** substantially the **share of renewable energy** in the global energy mix
7.3	By 2030, **double** the global **rate of improvement in energy efficiency**
7.a	By 2030, **enhance international cooperation to facilitate access to clean energy research and technology**, including renewable energy, energy efficiency and advanced and cleaner fossil-fuel technology, and promote investment in energy infrastructure and clean energy technology
7.b	By 2030, **expand infrastructure and upgrade technology for supplying modern and sustainable energy services** for all in developing countries, in particular least developed countries, small island developing States and landlocked developing countries, in accordance with their respective programmes of support
Goal 8. Promote sustained, inclusive and sustainable economic growth, full and productive employment and decent work for all	
8.1	**Sustain per capita economic growth** in accordance with national circumstances and, in particular, at least 7 per cent gross domestic product growth per annum in the least developed countries
8.2	**Achieve higher** levels of **economic productivity** through diversification, technological upgrading and innovation, including through a focus on high-value added and labour-intensive sectors
8.3	**Promote development-oriented policies** that **support productive activities, decent job creation, entrepreneurship, creativity and innovatio**n, and encourage the **formalization and growth of micro-, small- and medium-sized enterprises**, including through access to financial services
8.4	**Improve** progressively, through 2030, **global resource efficiency in consumption and production** and endeavour to **decouple economic growth from environmental degradation**, in accordance with the 10-Year Framework of Programmes on Sustainable Consumption and Production, with developed countries taking the lead
8.5	By 2030, **achieve full and productive employment and decent work** for all women and men, including for young people and persons with disabilities, and **equal pay for work of equal value**
8.6	By 2020, substantially **reduce the proportion of youth not in employment, education or training**
8.7	Take immediate and effective **measures to eradicate forced labour, end modern slavery and human trafficking** and s**ecure the prohibition and elimination of the worst forms of child labour**, including recruitment and use of child soldiers, and by 2025 end child labour in all its forms

Goals and targets	Keywords
8.8	**Protect labour rights** and p**romote safe and secure working environments** for all workers, including migrant workers, in particular women migrants, and those in precarious employment
8.9	By 2030, devise and **implement policies to promote sustainable tourism** that creates jobs and promotes local culture and products
8.10	**Strengthen the capacity of domestic financial institutions** to encourage and expand access to **banking, insurance and financial services for all**
8.a	**Increase Aid for Trade support for developing countries**, in particular least developed countries, including through the Enhanced Integrated Framework for Traderelated Technical Assistance to Least Developed Countries
8.b	By 2020, **develop and operationalize a global strategy for youth employment** and implement the Global Jobs Pact of the International Labour Organization
Goal 9. Build resilient infrastructure, promote inclusive and sustainable industrialization and foster innovation	
9.1	**Develop** quality, reliable, sustainable and resilient **infrastructure**, including regional and transborder infrastructure, **to support economic development and human well-being**, with a focus on **affordable and equitable access for all**
9.2	**Promote inclusive and sustainable industrialization** and, by 2030, significantly **raise industry's share of employment and gross domestic product**, in line with national circumstances, and double its share in least developed countries
9.3	**Increase the access of small-scale industrial and other enterprises**, in particular in developing countries, **to financial services**, including affordable credit, and their integration into value chains and markets
9.4	By 2030, **upgrade infrastructure and retrofit industries to make them sustainable**, with **increased resource-use efficiency** and greater **adoption of clean and environmentally sound technologies and industrial processes**, with all countries taking action in accordance with their respective capabilities
9.5	**Enhance scientific research**, **upgrade the technological capabilities of industrial sectors** in all countries, in particular developing countries, including, by 2030, encouraging innovation and substantially increasing the number of research and development workers per 1 million people and public and private research and development spending
9.a	**Facilitate sustainable and resilient infrastructure development** in developing countries through **enhanced financial, technological and technical support** to African countries, least developed countries, landlocked developing countries and small island developing States
9.b	**Support domestic technology development**, **research and innovation** in developing countries, including by ensuring a conducive policy environment for, inter alia, industrial diversification and value addition to commodities
9.c	**Significantly increase access to information and communications technology** and strive to provide **universal and affordable access to the Internet** in least developed countries by 2020
Goal 10. Reduce inequality within and among countries	
10.1	By 2030, progressively **achieve and sustain income growth of the bottom 40 per cent of the population** at a rate higher than the national average
10.2	By 2030, **empower and promote the social, economic and political inclusion of all**, irrespective of age, sex, disability, race, ethnicity, origin, religion or economic or other status
10.3	**Ensure equal opportunity and reduce inequalities of outcome**, including by eliminating discriminatory laws, policies and practices and promoting appropriate legislation, policies and action in this regard
10.4	**Adopt policies, especially fiscal, wage and social protection policies**, and progressively **achieve greater equality**
10.5	**Improve the regulation and monitoring of global financial markets and institutions** and strengthen the implementation of such regulations
10.6	**Ensure enhanced representation and voice for developing countries in decision-making in global international economic and financial institutions** in order to deliver more effective, credible, accountable and legitimate institutions
10.7	**Facilitate orderly, safe, regular and responsible migration and mobility of people**, including through the implementation of planned and well-managed migration policies
10.a	**Implement the principle of special and differential treatment for developing countries**, in particular least developed countries, in accordance with World Trade Organization agreements
10.b	**Encourage official development assistance and financial flows**, including foreign direct investment, to States where the need is greatest, in particular least developed countries, African countries, small island developing States and landlocked developing countries, in accordance with their national plans and programmes
10.c	By 2030, **reduce to less than 3 per cent the transaction costs of migrant remittances and eliminate remittance corridors** with costs higher than 5 per cent
Goal 11. Make cities and human settlements inclusive, safe, resilient and sustainable	
11.1	By 2030, **ensure access** for all to **adequate, safe and affordable housing and basic services and upgrade slums**
11.2	By 2030, **provide access to safe, affordable, accessible and sustainable transport systems for all**, improving road safety, notably by expanding public transport, with special attention to the needs of those in vulnerable situations, women, children, persons with disabilities and older persons
11.3	By 2030, **enhance inclusive and sustainable urbanization and capacity** for participatory, integrated and sustainable human settlement planning and management in all countries
11.4	Strengthen efforts to **protect and safeguard the world's cultural and natural heritage**

Goals and targets	Keywords
11.5	By 2030, significantly **reduce** the number of **deaths** and the number of **people affected** and substantially **decrease the direct economic losses relative to global gross domestic product caused by disasters**, including water-related disasters, with a focus on protecting the poor and people in vulnerable situations
11.6	By 2030, **reduce the adverse per capita environmental impact of cities**, including by paying special attention to air quality and municipal and other waste management
11.7	By 2030, **provide** universal **access to safe, inclusive and accessible, green and public spaces**, in particular for women and children, older persons and persons with disabilities
11.a	**Support positive economic, social and environmental links between urban, peri-urban and rural areas** by strengthening national and regional development planning
11.b	By 2020, substantially **increase** the number of **cities and human settlements adopting and implementing integrated policies** and **plans towards inclusion, resource efficiency, mitigation and adaptation to climate change, resilience to disasters, and develop and implement**, in line with the Sendai Framework for Disaster Risk Reduction 2015–2030, holistic disaster risk management at all levels
11.c	**Support** least developed countries, **including through financial and technical assistance**, in **building sustainable and resilient buildings utilizing local materials**
Goal 12. Ensure sustainable consumption and production patterns	
12.1	Implement the **10-Year Framework of Programmes on Sustainable Consumption and Production Patterns**, all countries taking action, with developed countries taking the lead, taking into account the development and capabilities of developing countries
12.2	By 2030, **achieve the sustainable management and efficient use of natural resources**
12.3	By 2030, **halve per capita global food waste** at the retail and consumer levels and **reduce food losses along production and supply chains**, including post-harvest losses
12.4	By 2020, **achieve the environmentally sound management of chemicals and all wastes throughout their life cycle**, in accordance with agreed international frameworks, and significantly **reduce their release to air, water and soil** in order to minimize their adverse impacts on human health and the environment
12.5	By 2030, substantially **reduce waste generation through prevention, reduction, recycling and reuse**
12.6	**Encourage companies**, especially large and transnational companies, to **adopt sustainable practices** and **to integrate sustainability information into their reporting cycle**
12.7	**Promote public procurement practices that are sustainable**, in accordance with national policies and priorities
12.8	By 2030, **ensure that people** everywhere **have** the relevant **information and awareness for sustainable development** and **lifestyles in harmony with nature**
12.a	**Support** developing countries to **strengthen** their **scientific and technological capacity** to move towards more **sustainable patterns of consumption and production**
12.b	**Develop and implement tools** to **monitor sustainable development impacts for sustainable tourism** that creates jobs and promotes local culture and products
12.c	**Rationalize inefficient fossil-fuel subsidies that encourage wasteful consumption** by removing market distortions, in accordance with national circumstances, including by restructuring taxation and phasing out those harmful subsidies, where they exist, to reflect their environmental impacts, taking fully into account the specific needs and conditions of developing countries and minimizing the possible adverse impacts on their development in a manner that protects the poor and the affected communities
Goal 13. Take urgent action to combat climate change and its impacts	
13.1	**Strengthen resilience and adaptive capacity** to **climate-related hazards** and **natural disasters** in all countries
13.2	**Integrate climate change measures** into **national policies, strategies and planning**
13.3	Improve **education, awareness-raising and human and institutional capacity** on **climate change mitigation, adaptation, impact reduction and early warning**
13.a	Implement the commitment undertaken by developed country parties to the **United Nations Framework Convention on Climate Change** to a goal of mobilizing jointly $100 billion annually by 2020 from all sources to address the needs of developing countries in the context of **meaningful mitigation actions and transparency on implementation and fully operationalize the Green Climate Fund through its capitalization** as soon as possible
13.b	**Promote mechanisms** for **raising capacity for effective climate change-related planning and management** in least developed countries and small island developing States, including focusing on women, youth and local and marginalized communities
Goal 14. Conserve and sustainably use the oceans, seas and marine resources for sustainable development	
14.1	By 2025, prevent and significantly **reduce marine pollution** of all kinds, in particular from land-based activities, including marine debris and nutrient pollution
14.2	By 2020, sustainably **manage and protect marine and coastal ecosystems to avoid significant adverse impacts**, including by strengthening their resilience, and take action for their restoration in order to achieve healthy and productive oceans
14.3	**Minimize and address the impacts of ocean acidification**, including through enhanced scientific cooperation at all levels
14.4	By 2020, effectively **regulate harvesting and end overfishing**, illegal, unreported and unregulated fishing and destructive fishing practices and implement science-based management plans, in order to restore fish stocks in the shortest time feasible, at least to levels that can produce maximum sustainable yield as determined by their biological characteristics
14.5	By 2020, **conserve** at least 10 per cent of **coastal and marine areas**, consistent with national and international law and based on the best available scientific information

Goals and targets	Keywords
14.6	By 2020, **prohibit** certain forms of **fisheries subsidies which contribute to overcapacity and overfishing, eliminate subsidies that contribute to illegal, unreported and unregulated fishing and refrain from introducing new such subsidies**, recognizing that appropriate and effective special and differential treatment for developing and least developed countries should be an integral part of the World Trade Organization fisheries subsidies negotiation
14.7	By 2030, **increase the economic benefits to small island developing States** and least developed countries **from the sustainable use of marine resources**, including through sustainable management of fisheries, aquaculture and tourism
14.a	**Increase scientific knowledge, develop research capacity and transfer marine technology**, taking into account the Intergovernmental Oceanographic Commission Criteria and Guidelines on the Transfer of Marine Technology, in order **to improve ocean health and to enhance the contribution of marine biodiversity** to the development of developing countries, in particular small island developing States and least developed countries
14.b	**Provide access for small-scale artisanal fishers to marine resources and markets**
14.c	**Enhance the conservation and sustainable use of oceans and their resources** by **implementing international law** as reflected in the United Nations Convention on the Law of the Sea, which provides the legal framework for the conservation and sustainable use of oceans and their resources, as recalled in paragraph 158 of "The future we want"
Goal 15. Protect, restore and promote sustainable use of terrestrial ecosystems, sustainably manage forests, combat desertification, and halt and reverse land degradation and halt biodiversity loss	
15.1	By 2020, **ensure the conservation, restoration and sustainable use of terrestrial and inland freshwater ecosystems and their services**, in particular forests, wetlands, mountains and drylands, in line with obligations under international agreements
15.2	By 2020, **promote** the implementation of **sustainable management of** all types of **forests**, **halt deforestation, restore degraded forests** and substantially **increase afforestation and reforestation** globally
15.3	By 2030, **combat desertification**, **restore degraded land and soi**l, including land affected by desertification, drought and floods, and strive to achieve a land degradation neutral world
15.4	By 2030, **ensure the conservation of mountain ecosystems**, including their biodiversity, in order to enhance their capacity to provide benefits that are essential for sustainable development
15.5	Take urgent and significant action to **reduce the degradation of natural habitats, halt the loss of biodiversity** and, by 2020, protect and **prevent the extinction of threatened species**
15.6	**Promote** fair and equitable sharing of the **benefits arising from** the **utilization of genetic resources** and **promote** appropriate **access to such resources**, as internationally agreed
15.7	Take urgent action to **end poaching** and t**rafficking of protected species of flora and fauna** and **address** both **demand and supply of illegal wildlife products**
15.8	By 2020, introduce measures to **prevent the introduction** and significantly **reduce the impact of invasive alien species on land and water ecosystems** and **control or eradicate the priority species**
15.9	By 2020, **integrate ecosystem and biodiversity values into national and local planning, development processes, poverty reduction strategies and accounts**
15.a	Mobilize and significantly **increase financial resources** from all sources to **conserve and sustainably use biodiversity and ecosystems**
15.b	**Mobilize significant resources** from all sources and at all levels to **finance sustainable forest management** and **provide adequate incentives** to developing countries to advance such management, including for conservation and reforestation
15.c	Enhance **global support for efforts** to **combat poaching and trafficking of protected species**, including by increasing the capacity of local communities to pursue sustainable livelihood opportunities
Goal 16. Promote peaceful and inclusive societies for sustainable development, provide access to justice for all and build effective, accountable and inclusive institutions at all levels	
16.1	Significantly **reduce** all forms of **violence and related death rates** everywhere
16.2	**End abuse, exploitation, trafficking and all forms of violence** against and torture of children
16.3	**Promote** the **rule of law** at the national and international levels and **ensure equal access to justice for all**
16.4	By 2030, significantly **reduce illicit financial and arms flows**, **strengthen the recovery** and **return of stolen assets** and **combat** all forms of **organized crime**
16.5	Substantially **reduce corruption and bribery** in all their forms
16.6	**Develop effective, accountable and transparent institutions** at all levels
16.7	**Ensure responsive, inclusive, participatory and representative decision-making** at all levels
16.8	Broaden and strengthen **the participation of developing countries in the institutions of global governance**
16.9	By 2030, **provide legal identity for all**, including birth registration
16.10	**Ensure public access to information and protect fundamental freedoms**, in accordance with national legislation and international agreements
16.a	**Strengthen relevant national institutions**, including through international cooperation, **for building capacity** at all levels, in particular in developing countries**, to prevent violence** and **combat terrorism and crime**
16.b	Promote and enforce **non-discriminatory laws and policies for sustainable development**

Goals and targets	Keywords
Goal 17. Strengthen the means of implementation and revitalize the Global Partnership for Sustainable Development	
17.1	**Strengthen domestic resource mobilization**, including through international support to developing countries, to **improve domestic capacity for tax** and **other revenue collection**
17.2	**Developed countries** to implement fully their **official development assistance commitments**, including the commitment by many developed countries to achieve the target of 0.7 per cent of gross national income for official development assistance (ODA/GNI) to developing countries and 0.15 to 0.20 per cent of ODA/GNI to least developed countries; ODA providers are encouraged to consider setting a target to provide at least 0.20 per cent of ODA/GNI to least developed countries
17.3	**Mobilize additional financial resources for developing countries** from multiple sources
17.4	**Assist developing countries** in **attaining long-term debt sustainability** through **coordinated policies** aimed at fostering debt financing, debt relief and debt restructuring, as appropriate, and address the external debt of highly indebted poor countries to reduce debt distress
17.5	Adopt and implement **investment promotion regimes** for least developed countries
17.6	**Enhance** North-South, South-South and triangular regional and **international cooperation on and access to science, technology and innovation and enhance knowledge sharing** on mutually agreed terms, including through improved coordination among existing mechanisms, in particular at the United Nations level, and through a global technology facilitation mechanism
17.7	Promote the **development, transfer, dissemination and diffusion of environmentally sound technologies** to developing countries on favourable terms, including on concessional and preferential terms, as mutually agreed
17.8	**Fully operationalize** the **technology bank and science, technology and innovation capacity-building mechanism** for least developed countries by 2017 and enhance the use of enabling technology, in particular information and communications technology
17.9	**Enhance international support** for **implementing effective and targeted capacity-building in developing countries** to support national plans to implement all the Sustainable Development Goals, including through North-South, South-South and triangular cooperation
17.10	**Promote a universal, rules-based, open, non-discriminatory and equitable multilateral trading system** under the World Trade Organization, including through the conclusion of negotiations under its Doha Development Agenda
17.11	Significantly **increase the exports of developing countries**, in particular with a view to doubling the least developed countries' share of global exports by 2020
17.12	**Realize timely implementation of duty-free and quota-free market access on a lasting basis** for all least developed countries, consistent with World Trade Organization decisions, including by ensuring that preferential rules of origin applicable to imports from least developed countries are transparent and simple, and contribute to facilitating market access
17.13	**Enhance global macroeconomic stability**, including through policy coordination and policy coherence
17.14	**Enhance policy coherence for sustainable development**
17.15	**Respect each country's policy space and leadership** to establish and implement policies for poverty eradication and sustainable development
17.16	**Enhance** the **Global Partnership for Sustainable Development**, complemented by multi-stakeholder partnerships that mobilize and share knowledge, expertise, technology and financial resources, to support the achievement of the Sustainable Development Goals in all countries, in particular developing countries
17.17	**Encourage and promote effective public, publicprivate and civil society partnerships**, building on the experience and resourcing strategies of partnerships
17.18	By 2020, **enhance capacity-building support to developing countries**, including for least developed countries and small island developing States, to **increase** significantly the **availability of high-quality, timely and reliable data** disaggregated by income, gender, age, race, ethnicity, migratory status, disability, geographic location and other characteristics relevant in national contexts
17.19	By 2030, **build on existing initiatives** to **develop measurements of progress on sustainable development** that complement gross domestic product, and support statistical capacity-building in developing countries

Appendix B. Subbasins of the six rivers in China

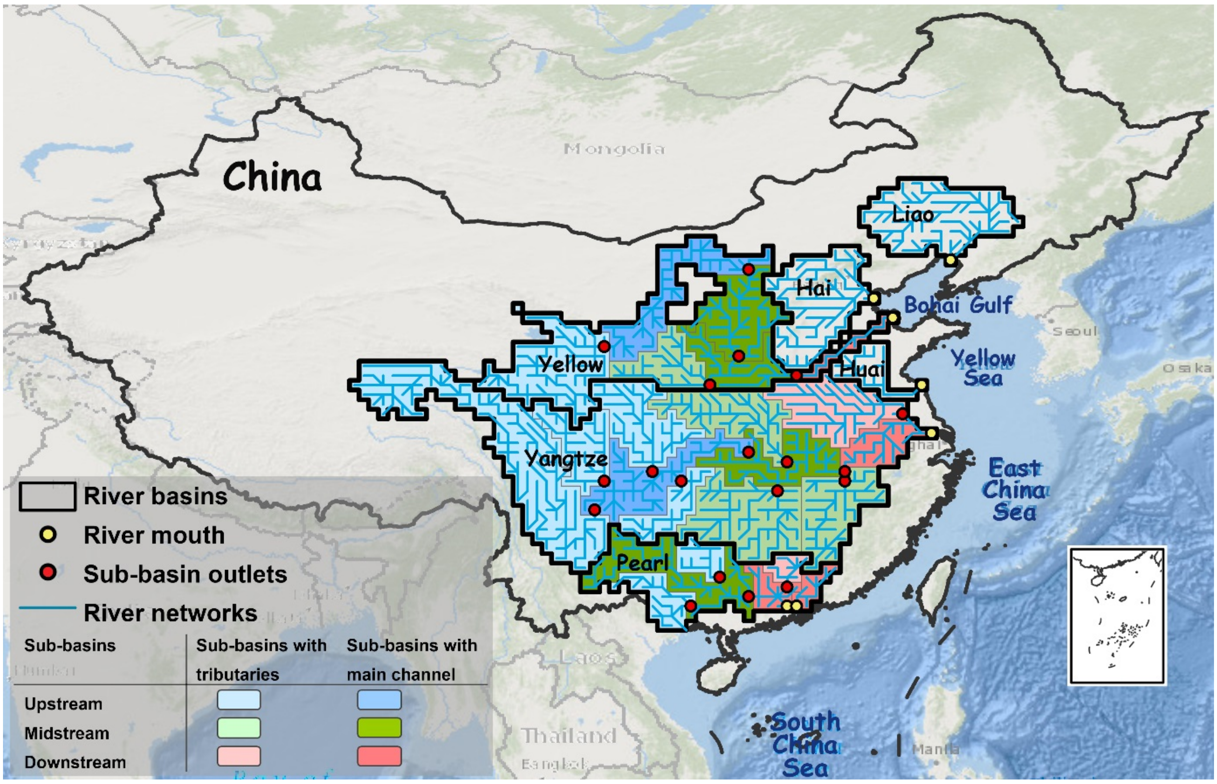

Supplementary Figure 1 Overview of the study area. We consider six large Chinese rivers: the Liao, Hai and Yellow rivers draining into the Bohai Gulf; the Huai River draining into the Yellow Sea; the Yangtze River draining into the East China Sea; and the Pearl River draining into the South China Sea. River drainage areas are delineated using the global drainage direction map (DDM-30, 30 arc minute resolution) (Döll and Lehner, 2002). The country boundary is from (RESDC, 2013).

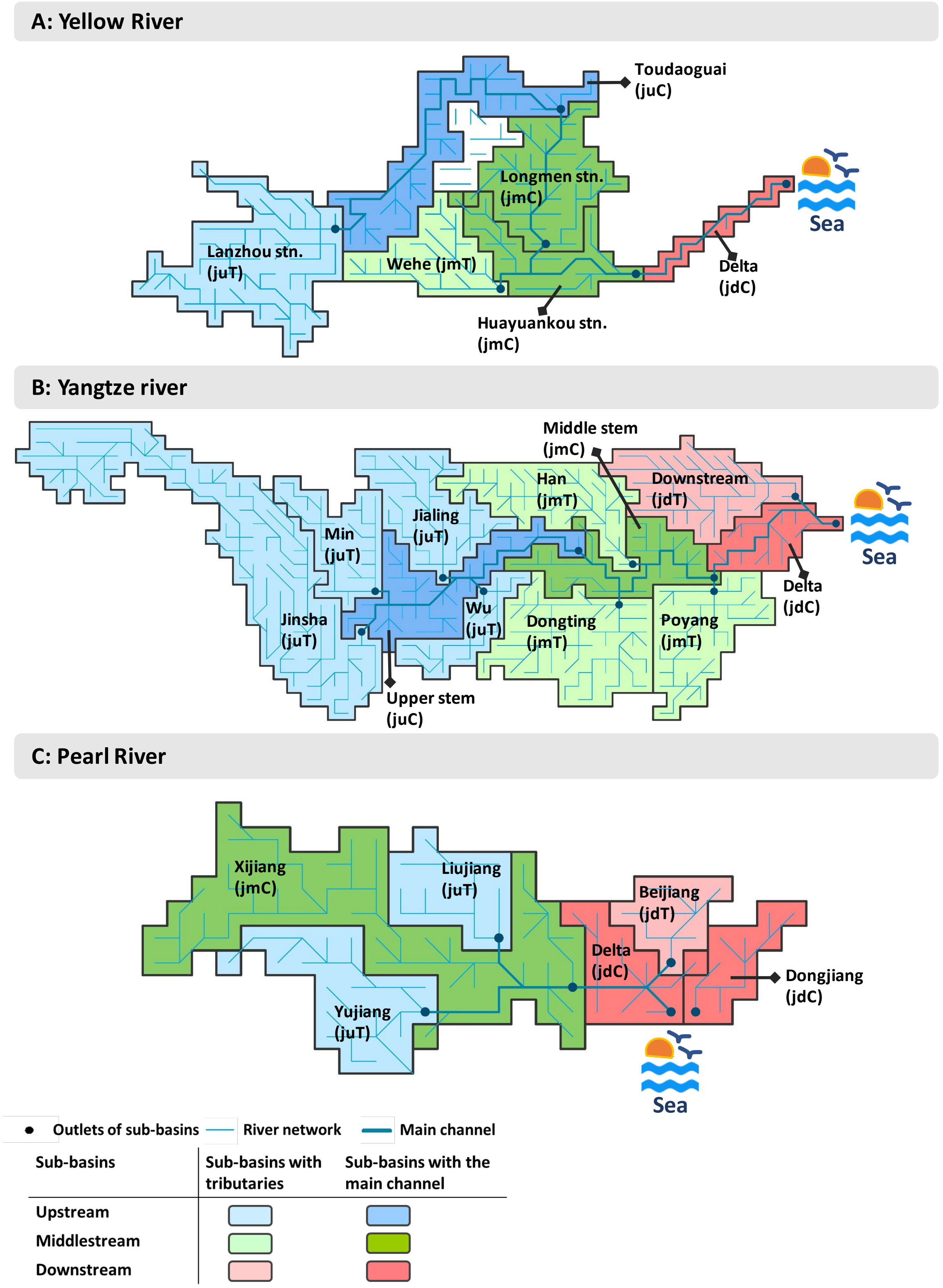

Supplementary Figure 2 Subbasins of the Yellow, Yangtze and Pearl Rivers in China. The delineation of the subbasins is from the MARINA (Model to Assess River Inputs of Nutrients to seAs) 2.0 model based on the global drainage direction map (DDM-30, 30 arc minute resolution) (Döll and Lehner, 2002).

Appendix C. Scenarios

Supplementary Table 6 Description of the scenarios for 2050. For 2050, six scenarios were explored: SSP5-RCP8.5 (Baseline; Shared Socio-economic Pathway 5), SE (improved sewage treatment), AG (improved nutrient use efficiencies in agriculture), AG+SE (combination of AG and SE), AG+SE+SFC (sustainable food consumption in addition to AG+SE), AG+SE+SFC+CLI (climate mitigation in addition to AG+SE+SFC).

		SSP5-RCP8.5[1]	SE	AG	AG+SE	AG+SE+SFC	AG+SE+SFC+CLI
**Sewage systems**	Population connected to sewage systems	10% for rural 90% for urban	100% for rural and urban[2]	As in SSP5-RCP8.5	As in SE	As in SE	As in AG+SE+SFC
	Nutrient removal rates	N: 12-42% for rural, 44-67% for urban<br>P: 13-47% for rural, 49-75% for urban	N: 80% for rural and urban[2]<br>P: 90% for rural and urban[2]	As in SSP5-RCP8.5	As in SE	As in SE	As in AG+SE+SFC
**Agriculture**	Use of synthetic fertilizers	20% reduction for N and 28% reduction for P relative to 2012	As in SSP5-RCP8.5	According to the need by the crops[3]	According to the need by the crops[3]	According to the need by the crops[3]	According to the need by the crops[3]
	Recycling of straw residues	15-41%	As in SSP5-RCP8.5	80%	As in AG	As in AG	As in AG+SE+SFC
	N and P excretion	20% reduction for N and P relative to 2012	As in SSP5-RCP8.5	12% reduction for N and P relative to SSP5-RCP8.5[4]	As in AG	As in AG	As in AG+SE+SFC
	NH_3 emissions in manure storage	20% of N excretion	As in SSP5-RCP8.5	5% of N excretion[5]	As in AG	As in AG	As in AG+SE+SFC
	N_2O emissions in manure storage	0.5% of N excretion	As in SSP5-RCP8.5	0.25% of N excretion[5]	As in AG	As in AG	As in AG+SE+SFC
	Manure applications on land*	70%	As in SSP5-RCP8.5	100%	As in AG	As in AG	As in AG+SE+SFC
	Maure dischagre to waters*	30%	As in SSP5-RCP8.5	0%	As in AG	As in AG	As in AG+SE+SFC
	Export of manure	No export of manure outside the subbasins	As in SSP5-RCP8.5	As in SSP5-RCP8.5	As in AG	Remove excessive manure by transporting to other regions	As in AG+SE+SFC

	SSP5-RCP8.5	SE	AG	AG+SE	AG+SE+SFC	AG+SE+SFC+CLI
**Dietary choices**	Meat-rich diets	As in SSP5-RCP8.5	As in SSP5-RCP8.5	As in AG	Reduction 20% of crop and animal food demand Import 80% of the soybean consumption in SSP5-RCP8.5[6]	As in AG+SE+SFC
**Mitigating N deposition**	As in 2012	As in SSP5-RCP8.5	As in SSP5-RCP8.5	As in AG	Reduction of 50% relative to SSP5-RCP8.5	As in AG+SE+SFC
**Climate mitigation**	Poor climate mitigations, Green House Gas (GHG) concentration reaches the level of RCP8.5	As in SSP5-RCP8.5	As in SSP5-RCP8.5	As in AG	As in SSP5-RCP8.5	Mitigating the GHGs Emissions to the level of RCP2.6

* Application or discharge of manure after correcting for N losses to the air through ammonia (NH_3), nitrous oxide (N_2O) and denitrification during manure storage and housing.

[1] Baseline scenario. Assumptions in SSP5-RCP8.5 are from Wang et al. (2020). [2] According to “National urban sewage treatment and recycling planning in 13th Five-Year (2015–2020)” (NDRC-MOHURD, 2016), 85-95% of wastewater in urban or centers of counties will be connected to sewage systems in 2020. We, therefore, assume that in 2050, all wastewater in rural and urban areas will be connected to sewage systems with the best treatment technologies, same as in Strokal et al. (2017). [3] Use of synthetic fertilizers in crop production was derived as the need of nutrients by the crops based on the balanced fertilization approach in Ma et al. (2013), Wang et al. (2018). [4] We assumed that the N and P excretion by animals will reach the level of SSP1 inzby improving feed quality and raising animal species with higher nutrient use efficiencies. [5] We assumed that NH_3 and N_2O emissions are mitigated to the level of SSP1 in Wang et al. (2017), Wang et al. (2020). [6] We assumed China imports 80% of its soybean consumption in 2050 following the assumptions in Ma et al. (2019).

Supplementary Table 7 Overview of the interactions between SDGs (Sustainable Development Goals) for clean water (6 & 14) and SDGs 2, 11, 12 and 13 at the target level that are addressed in scenarios SE (improved sewage treatment), AG (improved nutrient use efficiencies in agriculture), AG+SE (combination of AG and SE), AG+SE+SFC (sustainable food consumption in addition to AG+SE), AG+SE+SFC+CLI (climate mitigation in addition to AG+SE+SFC). The interactions are shown as a bullet list. Descriptions of these interactions are in Supplementary Tables 3-4.

		SE	AG	AG+SE	AG+SE+SFC	AG+SE+SFC+CLI
**Sewage systems**	Population connected to sewage systems	100% for rural and urban:<br>• *6.2-11.1*<br>• *6.3-11.1*<br>• *6.3-11.6*<br>• *6.5-11.6*<br>• *6.6-11.6*<br>• *14.1-11.1*<br>• *14.5-11.1*<br>• *14.1-11.6*<br>• *14.5-11.6*	-	As in SE	As in SE	As in AG+SE+SFC
	Nutrient removal rates	N: 80% for rural and urban;<br>P: 90% for rural and urban:<br>• *6.2-11.1*<br>• *6.3-11.1*<br>• *6.3-11.6*<br>• *6.5-11.6*<br>• *6.6-11.6*<br>• *14.1-11.1*<br>• *14.5-11.1*<br>• *14.1-11.6*<br>• *14.5-11.6*	-	As in SE	As in SE	As in AG+SE+SFC

		SE	AG	AG+SE	AG+SE+SFC	AG+SE+SFC+CLI
**Agriculture**	Use of synthetic fertilizers	-	According to the need by the crops:<br>• *6.3-2.3*<br>• *6.3-2.4*<br>• *6.3-2.a*<br>• *14.1-2.3*<br>• *14.2-2.3*<br>• *14.3-2.3*<br>• *14.5-2.3*<br>• *14.1-2.4*<br>• *14.2-2.4*<br>• *14.3-2.4*<br>• *14.5-2.4*<br>• *14.1-2.a*	As in AG	As in AG	As in AG+SE+SFC
	Recycling of straw residues	-	80%	As in AG	As in AG	As in AG+SE+SFC
	N and P excretion	-	12% reduction for N and P relative to SSP5-RCP8.5:<br>• *6.3-2.a*<br>• *14.1-2.3*<br>• *14.2-2.3*<br>• *14.3-2.3*<br>• *14.1-2.4*<br>• *14.2-2.4*<br>• *14.3-2.4*<br>• *14.1-2.a*	As in AG	As in AG	As in AG+SE+SFC
	NH_3 emissions in manure storage	-	5% of N excretion	As in AG	As in AG	As in AG+SE+SFC
	N_2O emissions in manure storage	-	0.25% of N excretion	As in AG	As in AG	As in AG+SE+SFC

		SE	AG	AG+SE	AG+SE+SFC	AG+SE+SFC+CLI
**Agriculture**	Manure applications on land*	-	100%:<br>• *6.3-2.3*<br>• *6.3-2.4*<br>• *14.1-2.3*<br>• *14.2-2.3*<br>• *14.3-2.3*<br>• *14.1-2.4*<br>• *14.2-2.4*<br>• *14.3-2.4*<br>• *14.1-2.a*	As in AG	As in AG	As in AG+SE+SFC
	Maure dischagre to waters*	-	0%:<br>• *6.3-2.3*<br>• *6.3-2.4*<br>• *14.1-2.3*<br>• *14.2-2.3*<br>• *14.3-2.3*<br>• *14.1-2.4*<br>• *14.2-2.4*<br>• *14.3-2.4*<br>• *14.1-2.a*	As in AG	As in AG	As in AG+SE+SFC
	Export of manure	-	-	As in AG	Remove excessive manure by transporting to other regions:<br>• *6.3-2.3*<br>• *6.3-2.4*<br>• *14.1-2.3*<br>• *14.2-2.3*<br>• *14.3-2.3*<br>• *14.1-2.4*<br>• *14.2-2.4*<br>• *14.3-2.4*<br>• *14.1-2.a*	As in AG+SE+SFC

		SE	AG	AG+SE	AG+SE+SFC
**Dietary choices**	-	-	As in AG	Reduction 20% of crop and animal food demand; Import 80% of the soybean consumption in SSP5-RCP8.5:<br>• *6.3-12.1*<br>• *6.4-12.1*<br>• *6.5-12.1*<br>• *6.6-12.1*<br>• *6.3-12.2*<br>• *6.6-12.2*<br>• *6.3-12.3*<br>• *6.3-12.5*<br>• *6.3-12.8*<br>• *6.3-12.a*<br>• *14.1-12.1*<br>• *14.3-12.1*<br>• *14.5-12.1*<br>• *14.1-12.2*<br>• *14.1-12.2*<br>• *14.1-12.3*<br>• *14.5-12.3*<br>• *14.1-12.5*<br>• *14.5-12.5*<br>• *14.1-12.8*<br>• *14.2-12.8*<br>• *14.5-12.8*<br>• *14.1-12.a*<br>• *14.3-12.a*<br>• *14.5-12.a*	As in AG+SE+SFC
**Mitigating N deposition**	-	-	As in AG	Reduction of 50% relative to SSP5-RCP8.5	As in AG+SE+SFC

		SE	AG	AG+SE	AG+SE+SFC
**Climate mitigation**	-	-	As in AG	As in SSP5-RCP8.5	Mitigating the GHGs Emissions to the level of RCP2.6:<br>• *6.1-13.2*<br>• *6.3-13.2*<br>• *6.6-13.2*<br>• *6.1-13.3*<br>• *6.3-13.3*<br>• *6.6-13.3*<br>• *14.1-13.2*<br>• *14.2-13.2*<br>• *14.3-13.2*<br>• *14.5-13.2*<br>• *14.1-13.3*<br>• *14.2-13.3*<br>• *14.3-13.3*<br>• *14.5-13.3*

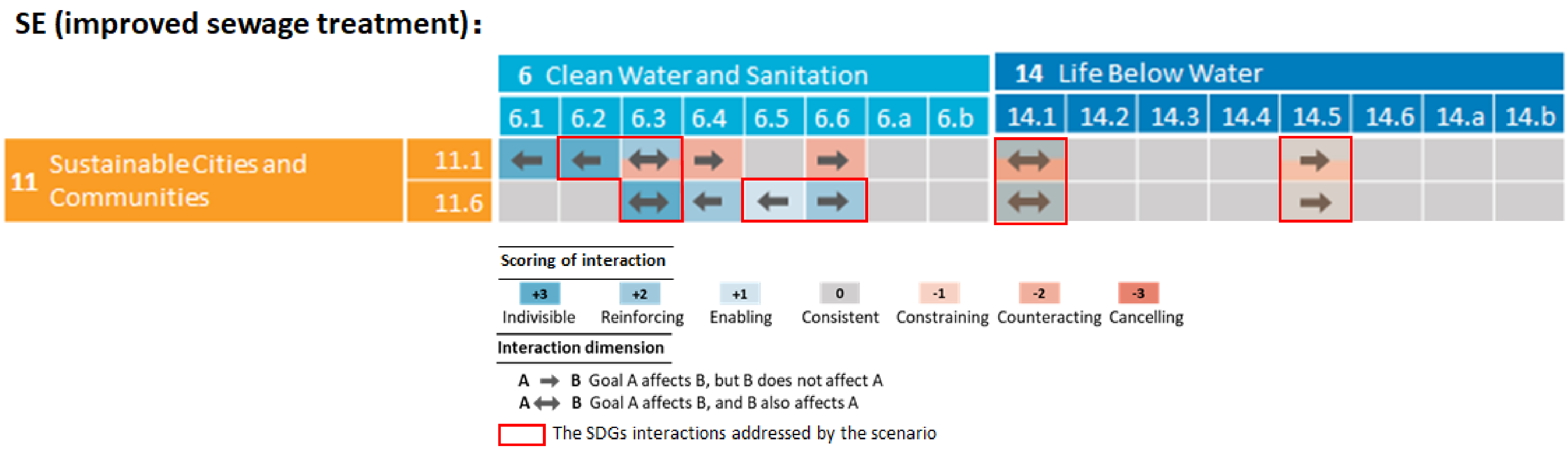

Supplementary Figure 3 The interactions between SDGs (Sustainable Development Goals) for clean water (6 & 14) and SDG 11 for sustainable cities and communities that are addressed (indicated by the red borders highlighting the selected boxes) by scenario SE (improved sewage treatment). The interactions between the SDGs are explained in Supplementary Tables 3-4. The assumptions in the SE scenario are in Supplementary Table 6. Supplementary Table 7 indicates which interactions are addressed in the SE scenario.

AG (improved nutrient use efficiencies in agriculture):

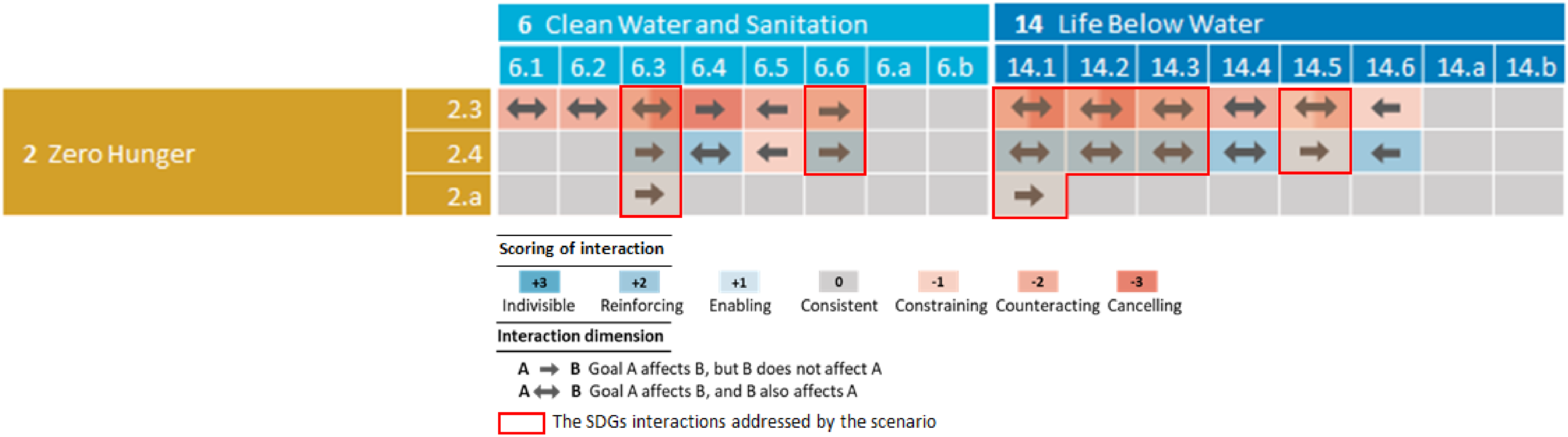

Supplementary Figure 4 The interactions between SDGs (Sustainable Development Goals) for clean water (6 & 14) and SDG 2 for no hunger that are addressed (indicated by the red borders highlighting the selected boxes) by scenario AG (improved nutrient use efficiencies in agriculture). The interactions between the SDGs are explained in Supplementary Tables 3-4. The assumptions in the AG scenario are in Supplementary Table 6. Supplementary Table 7 indicates which interactions are addressed in the AG scenario.

AG + SE (combination of AG and SE):

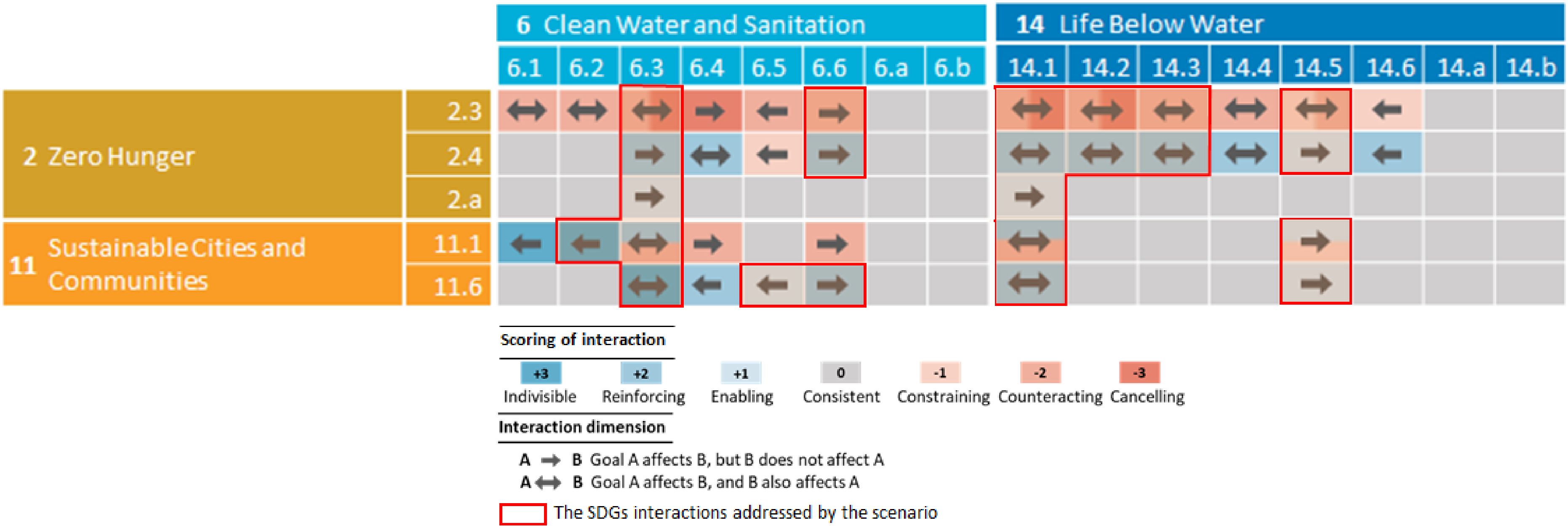

Supplementary Figure 5 The interactions between SDGs (Sustainable Development Goals) for clean water (6 & 14) and SDGs 2, 11 that are addressed (indicated by the red borders highlighting the selected boxes) by scenario AG +SE (combination of AG (improved nutrient use efficiencies in agriculture) and SE (improved sewage treatment)). The interactions between the SDGs are explained in Supplementary Tables 3-4. The assumptions in the AG+SE scenario are in Supplementary Table 6. Supplementary Table 7 indicates which interactions are addressed in the AG+SE scenario.

AG + SE + SFC (sustainable food consumption in addition to AG+SE):

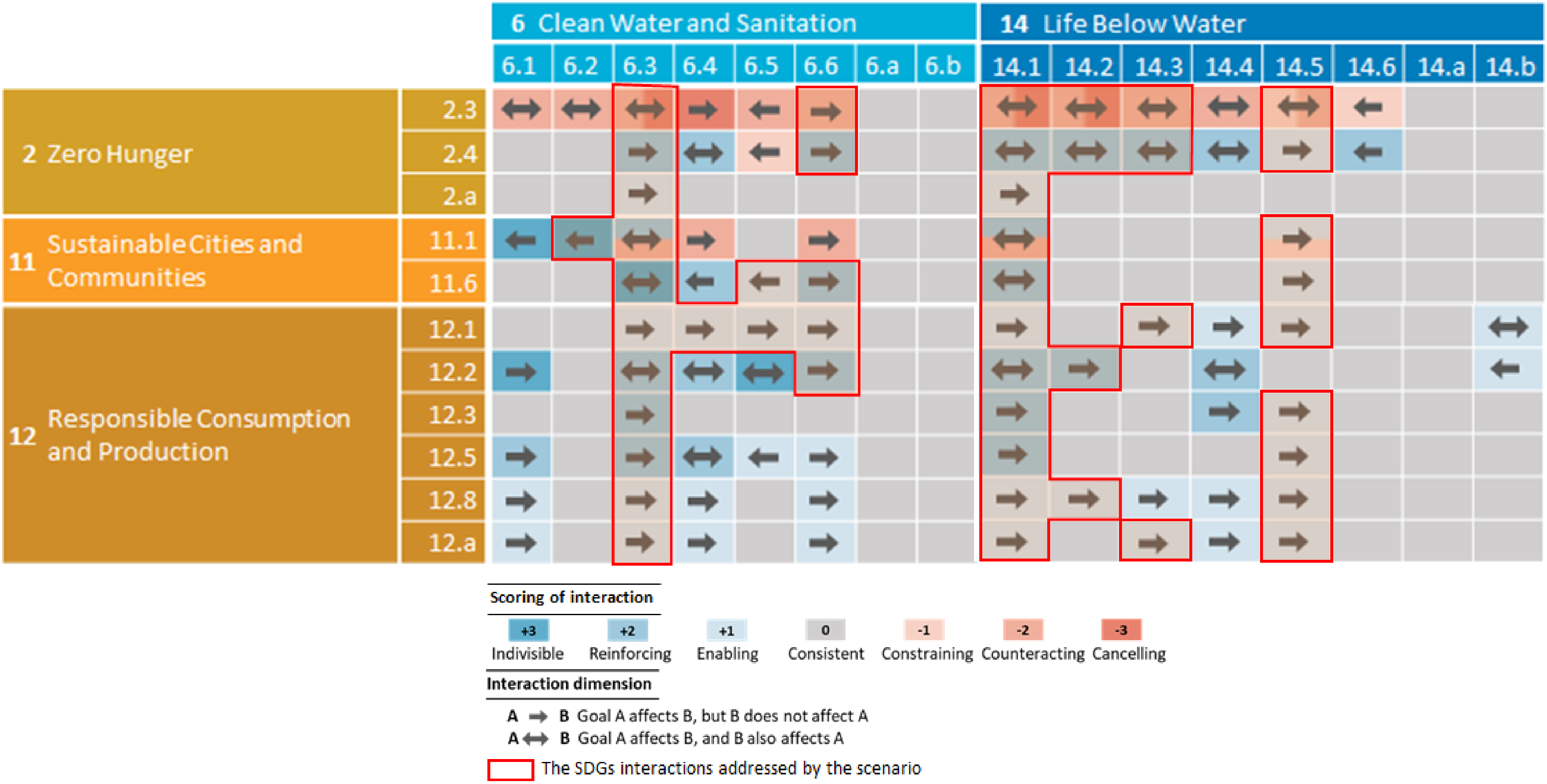

Supplementary Figure 6 The interactions between SDGs (Sustainable Development Goals) for clean water (6 & 14) and SDGs 2, 11, 12 that are addressed (indicated by the red borders highlighting the selected boxes) by scenario AG+SE+SFC (SFC (sustainable food consumption) in addition to AG (improved nutrient use efficiencies in agriculture) and SE (improved sewage treatment)). The interactions between the SDGs are explained in Supplementary Tables 3-4. The assumptions in the AG+SE+SFC scenario are in Supplementary Table 6. Supplementary Table 7 indicates which interactions are addressed in the AG+SE+SFC scenario.

AG + SE + SFC + CLI (climate mitigation in addition to AG+SE+SFC):

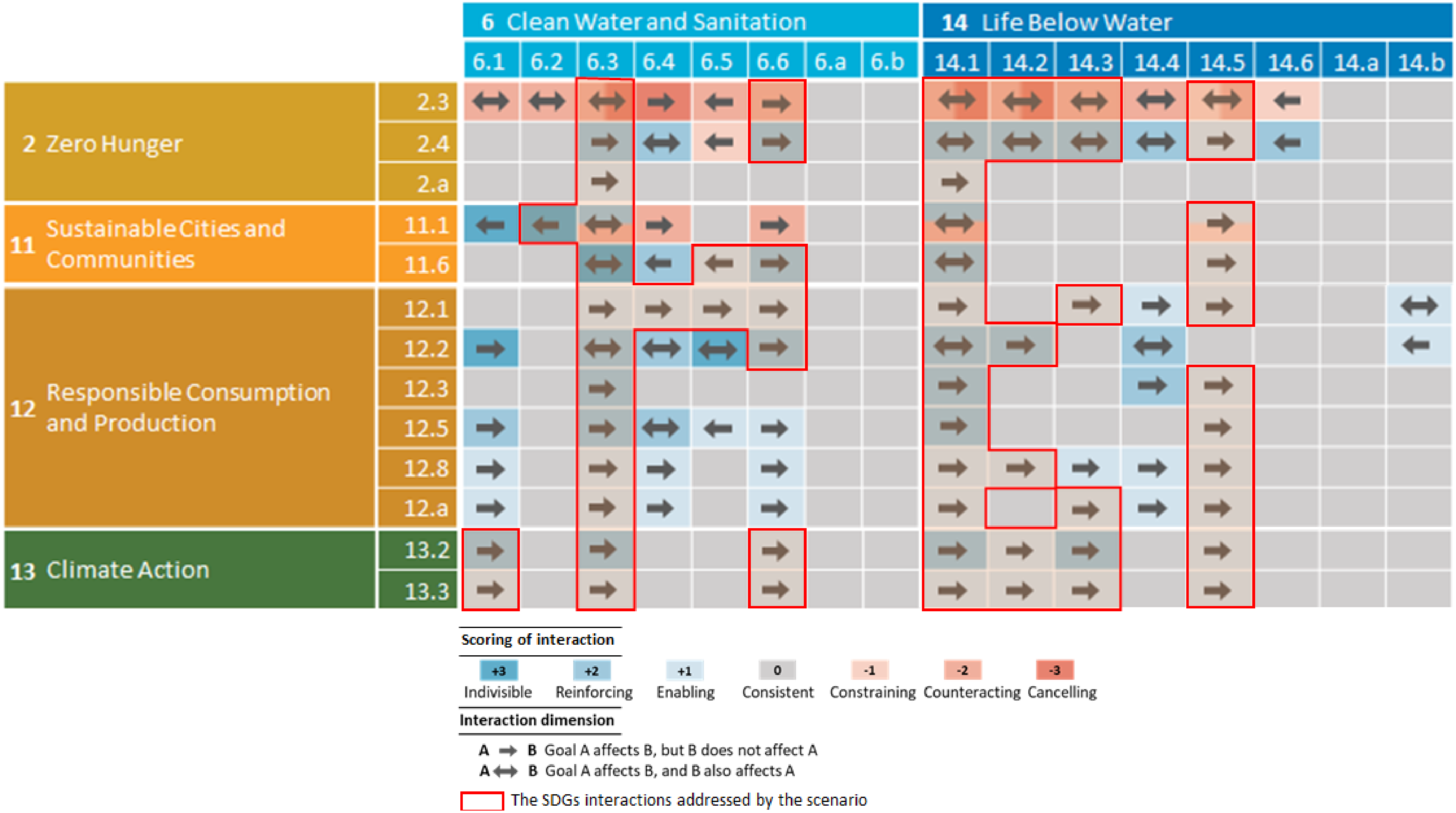

Supplementary Figure 7 The interactions between SDGs (Sustainable Development Goals) for clean water (6 & 14) and SDGs 2, 11, 12, 13 that are addressed (indicated by the red borders highlighting the selected boxes) by scenario AG+SE+SFC+CLI (Climate mitigation in addition to AG (improved nutrient use efficiencies in agriculture), SE (improved sewage treatment) and SFC (sustainable food consumption)). The interactions between the SDGs are explained in Supplementary Tables 3-4. The assumptions in the AG+SE+SFC+CLI scenario are in Supplementary Table 6. Supplementary Table 7 indicates which interactions are addressed in the AG+SE+SFC+CLI scenario.

C.1 Nutrient pollution in rivers and coastal waters

2012. Nutrient pollution levels in the six large rivers and coastal waters were relatively high in 2012. We calculate 18,553 kton of TDN and 2143 kton of TDP inputs to rivers (Figure 5). These nutrients are further transported by the rivers, leading to export of 4341 kton of TDN and 644 kton of TDP to the Chinese seas. More than half of the amounts of TDN and TDP are exported to the East China Sea (Figure 5). The rest is exported to the Bohai Gulf (15% of TDN, 23% of TDP), Yellow Sea (6% of TDN, 10% of TDP) and South China Sea (25% of TDP, 14% of TDP). River export of TDN and TDP in 2012 mainly origins from human activities on land including direct discharges of animal manure, use of synthetic fertilizers, and discharge of (treated or untreated) human waste (Figure 5, Supplementary Figures 8-12).

Baseline scenario SSP5-RCP8.5. In SSP5-RCP8.5, TDN input to rivers and river export of TDN to seas in 2050 are projected to remain as high as in 2012. However, TDP input to rivers in 2050 is 38% lower than that in 2012. As a result, river export of TDP is 42% lower compared to 2012. The decrease in P pollution can be largely explained by the improved manure management in SSP5-RCP8.5. In this scenario, recycling of animal manure (the amount available for application after losses during manure storage) is assumed to improve from 46% to 70% between 2012 and 2050 (Wang et al., 2017) (Supplementary Table 6). This reduces largely the direct discharge of manure to rivers in 2050. However, TDN input to rivers from the direct discharge of manure to rivers remains high in SSP5-RCP8.5. This is the net effect of increased manure recycling, and reduced ammonia (NH_3) emissions during manure storage and housing (increased N in manure available for application) in SSP5-RCP8.5 (Supplementary Table 6). Human activities on land (e.g., direct discharge of animal manure, use of synthetic fertilizers, and discharge of human waste) remain the dominant sources of river export of TDN and TDP to Chinese seas (Figure 5, Supplementary Figures 8-12).

Future nutrient pollution in rivers and coastal waters may be lower in 2050, but the level varies among the alternative scenarios (Figure 5). **AG+SE+SFC+CLI** is found to be the most effective scenario to improve water quality. Below we discuss the result of each scenario for 2050.

Alternative scenario SE. In this scenario, all wastewater from rural and urban areas is connected to sewage systems with relatively high nutrient removal efficiencies. Thus in 2050, TDN and TDP inputs to rivers from treated and untreated human waste is calculated to reduce by 54% and 79%, respectively, compared to SSP5-RCP8.5. River export of nutrients is 9% lower for TDN, and 25% lower for TDP in SE than in the baseline SSP5-RCP8.5.

Alternative scenario AG. Improved nutrient management in agriculture in AG is projected to be more effective than SE to reduce future nutrient pollution in rivers and coastal water. We quantified 12,091 kton of TDN and 551 kton of TDP inputs to rivers in 2050 in AG. River export of TDN is 39% lower than in the baseline SSP5-RCP8.5, and of TDP 63% lower. Nutrient pollution in rivers and coastal waters are much reduced by restricting the direct discharge of animal manure, recycling of manure on land, and reducing over-fertilization in crop production.

Alternative scenario AG+SE. This scenario combines the improved nutrient management of SE and AG, and thus combines their effectiveness in reducing nutrient pollution in rivers and coastal waters in 2050. Nutrient inputs to rivers in AG+SE are projected to be 42% lower for TDN, and 88% lower for TDP compared to the baseline SSP5-RCP8.5. River export of TDN is 48% lower, and of TDP is 89% lower than in SSP5-RCP8.5. N and P inputs to the subbasins from agriculture and human waste in this scenario.

Alternative scenario AG+SE+SFC. We calculate further reduction in nutrient pollution in rivers and coastal waters in this scenario compared to AG+SE. This scenario builds on AG+SE and assumes healthy and efficient food consumption, treatment of excessive manure, and mitigation of N deposition. Nutrient use in crop and animal production is lower than in AG+SE because of reduced consumption of meat, less food waste and import of soybeans. We calculate 7870 kton of TDN, and 46 kton of TDP inputs to rivers in 2050 in AG+SE+SFC. These nutrient inputs to rivers result in export of 1714 kton of TDN and 39 kton of TDP to seas. This amount is 60% and 90% lower for TDN and TDP, respectively, compared to the baseline SSP5-RCP8.5.

Alternative scenario AG+SE+SFC+CLI. This scenario is found to be the most effective to reduce nutrient pollution in rivers and coastal waters in China. In addition to the improve nutrient management in AG+SE+SFC, this scenario assumes optimistic climate mitigation by countries in the future. The climate-induced decreases in water runoff and discharge are projected to reduce TDN and TDP inputs to rivers by 15% and 9% for TDN and TDP, respectively, compared to AG+SE+SFC. As a result, in 2050, river export of TDN is 68% lower, and of TDP 91% lower in AG+SE+SEF+CLI than in SSP5-RCP8.5. Combing the best nutrient management in all other alternative scenarios, N and P inputs to rivers from human activities (i.e., agriculture and human waste) are much reduced in this scenario (Figure 5). Thus other sources (i.e., biological N_2 fixation, atmospheric N deposition, P weathering, and leaching of organic matters) may become important sources of nutrient pollution in rivers and coastal waters in 2050 (Figure 5, Supplementary Figures 8-12).

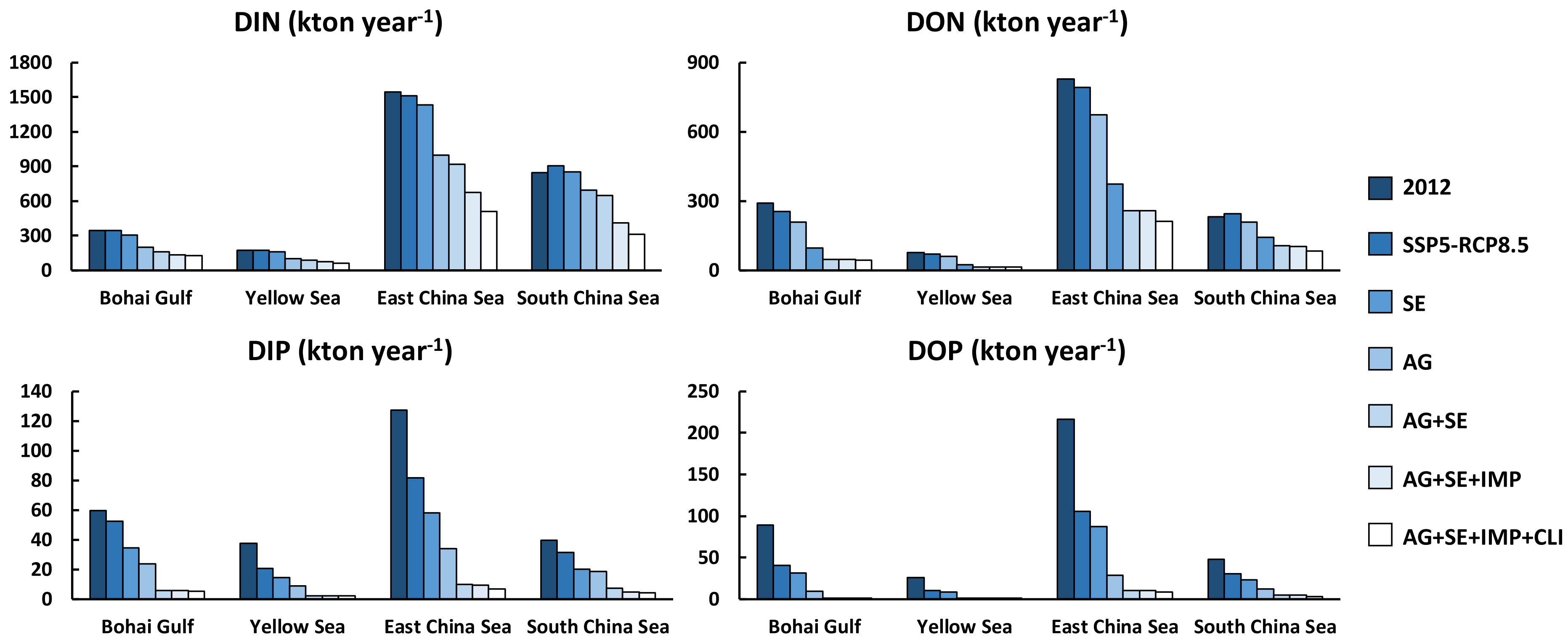

Supplementary Figure 8 River export of dissolved inorganic and organic nitrogen (DIN, DON) and phosphorus (DIP, DOP) from six large rives to the Bohai Gulf, Yellow Sea, East China Sea and South China Sea in 2012 and 2050 (kton year^{-1}). The six large rivers are: Liao, Hai, Yellow, Huai, Yangtze and Pearl rivers. For 2050, six scenarios were explored: Baseline SSP5-RCP8.5 (Shared Socio-economic Pathway 5), and alternative scenarios SE (improved sewage treatment), AG (improved nutrient use efficiencies in agriculture), AG+SE (a combination of AG and SE), AG+SE+SFC (sustainable food consumption in addition to AG+SE), AG+SE+SFC+CLI (climate mitigation in addition to AG+SE+SFC).

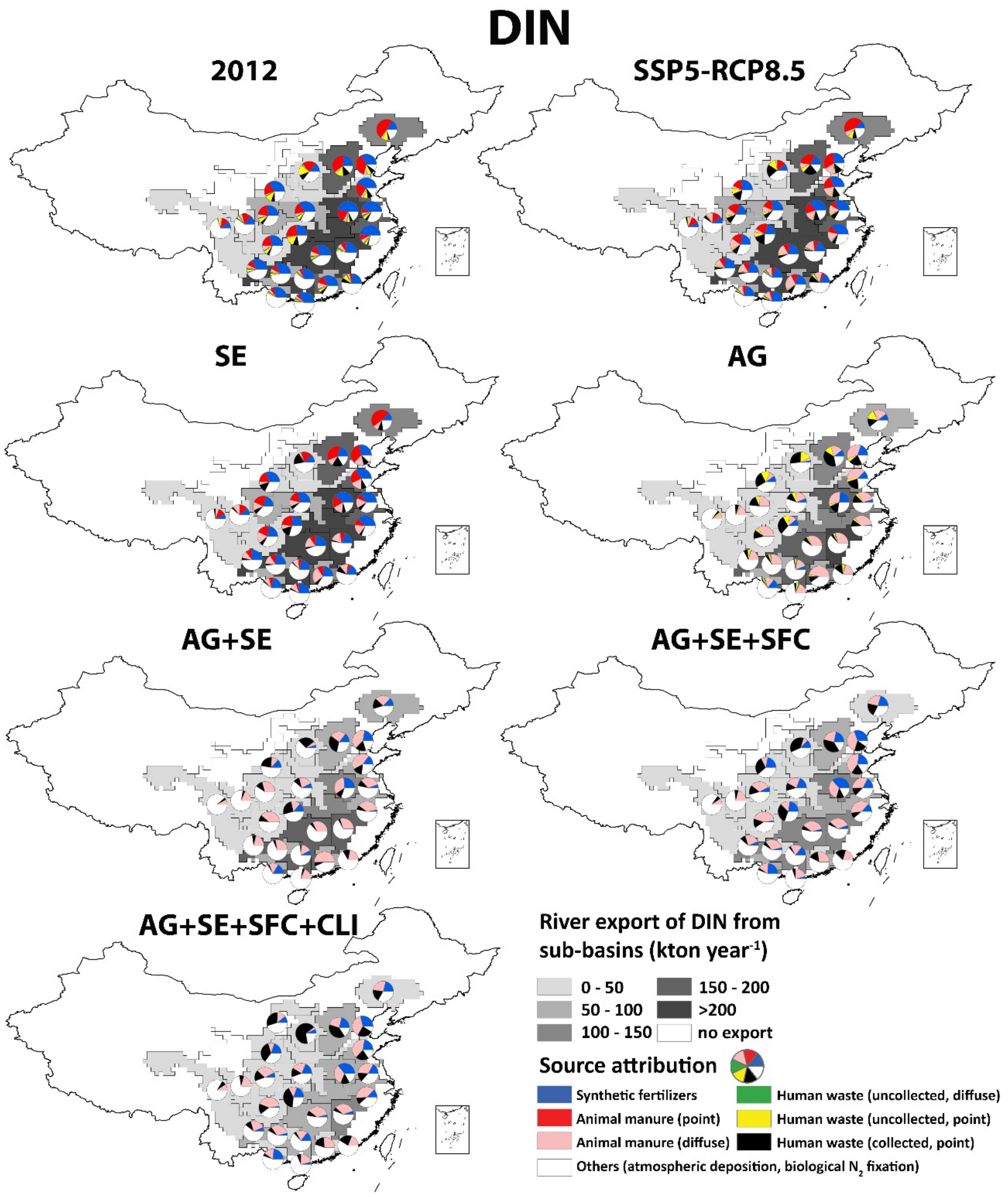

Supplementary Figure 9 River export of dissolved inorganic nitrogen (DIN) from subbasins of six large Chinese rivers by source in 2012 and 2050 (kton year^{-1}). The six large rivers are: Liao, Hai, Yellow, Huai, Yangtze and Pearl rivers. For 2050, six scenarios were explored: Baseline SSP5-RCP8.5 (Shared Socio-economic Pathway 5), and alternative scenarios SE (improved sewage treatment), AG (improved nutrient use efficiencies in agriculture), AG+SE (a combination of AG and SE), AG+SE+SFC (sustainable food consumption in addition to AG+SE), AG+SE+SFC+CLI (climate mitigation in addition to AG+SE+SFC).

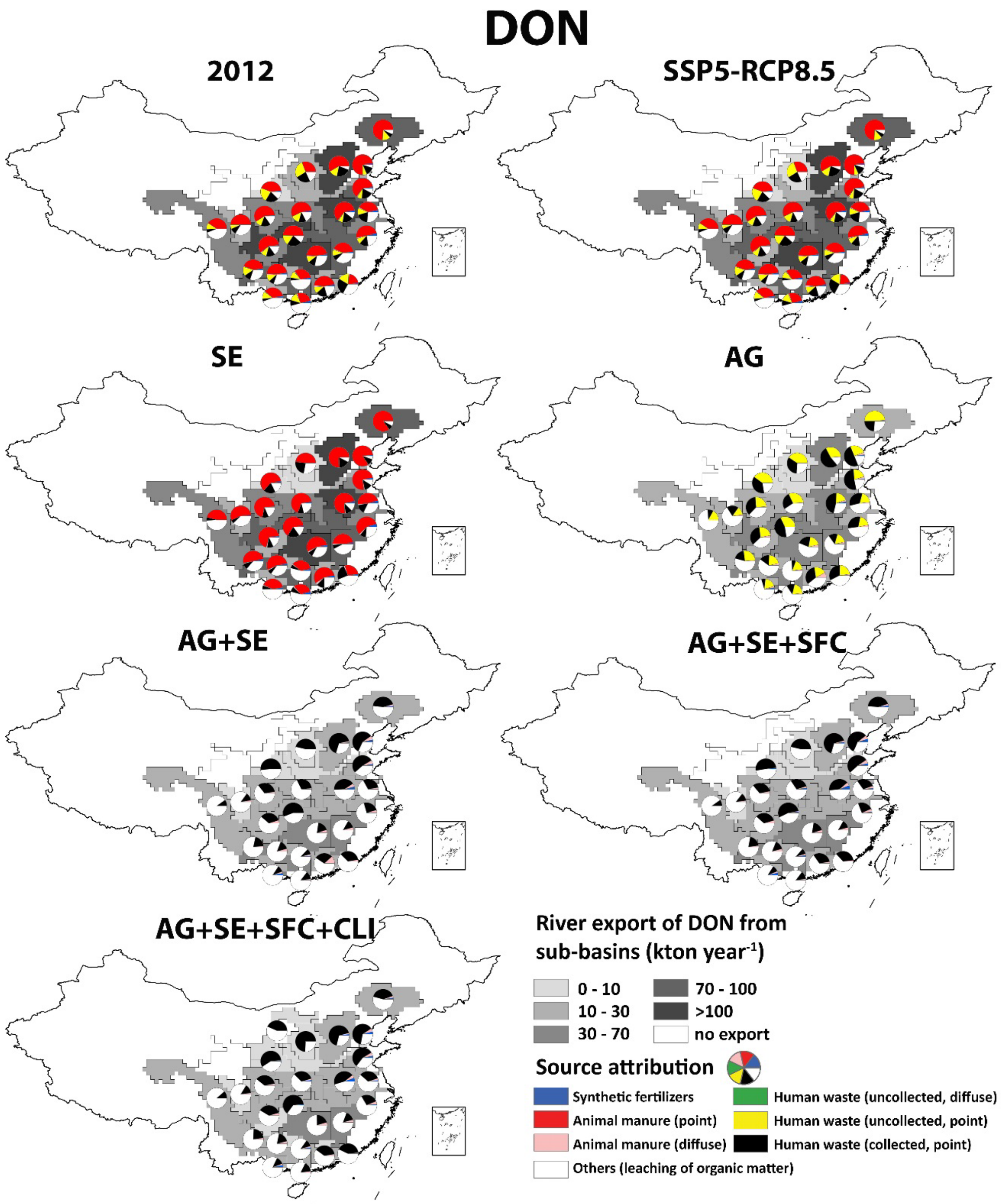

Supplementary Figure 10 River export of dissolved organic nitrogen (DON) from subbasins of six large Chinese rivers by source in 2012 and 2050 (kton year^{-1}). The six large rivers are: Liao, Hai, Yellow, Huai, Yangtze and Pearl rivers. For 2050, six scenarios were explored: Baseline SSP5-RCP8.5 (Shared Socio-economic Pathway 5), and alternative scenarios SE (improved sewage treatment), AG (improved nutrient use efficiencies in agriculture), AG+SE (a combination of AG and SE), AG+SE+SFC (sustainable food consumption in addition to AG+SE), AG+SE+SFC+CLI (climate mitigation in addition to AG+SE+SFC).

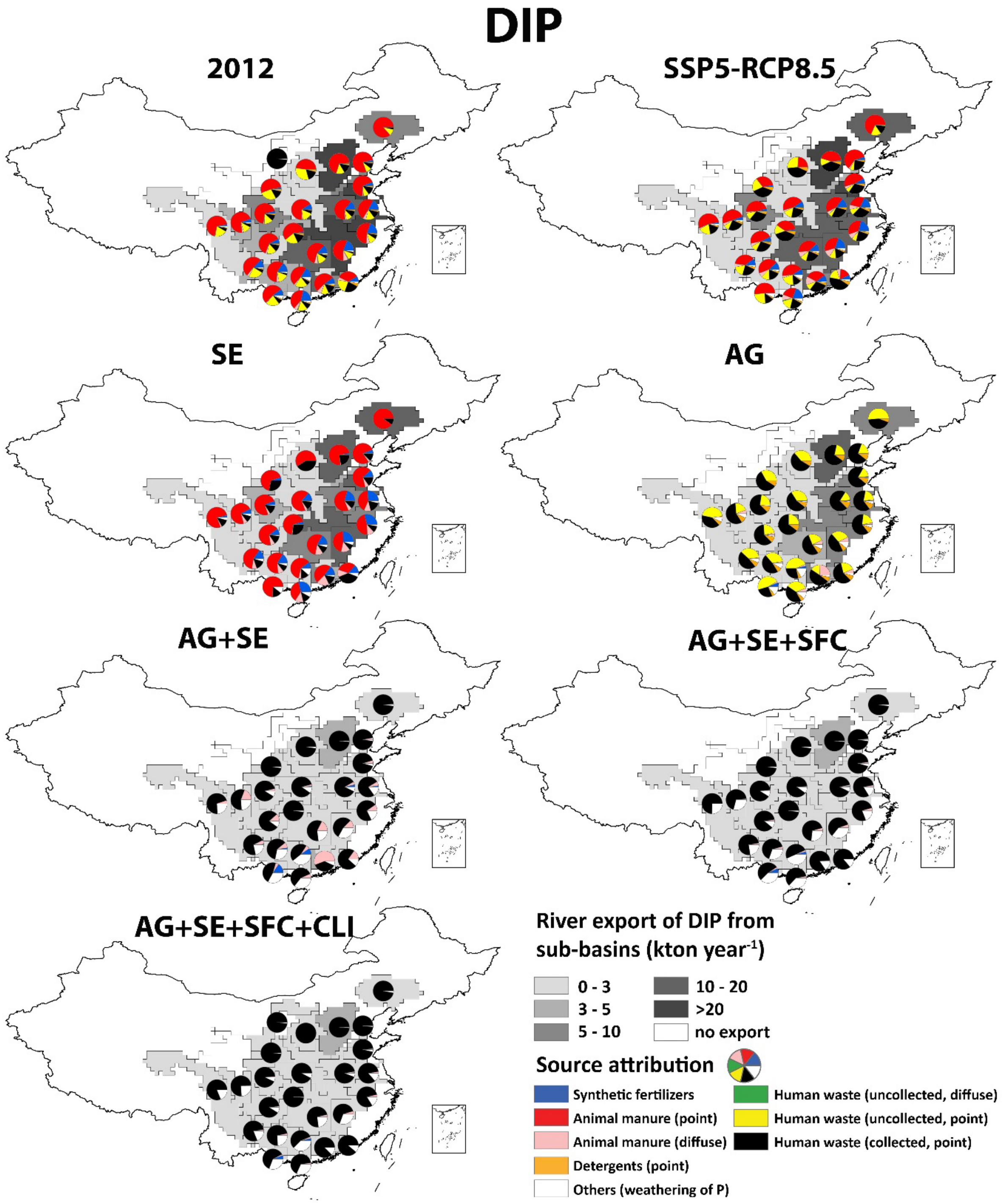

Supplementary Figure 11 River export of dissolved inorganic phosphorus (DIP) from subbasins of six large Chinese rivers by source in 2012 and 2050 (kton year^{-1}). The six large rivers are: Liao, Hai, Yellow, Huai, Yangtze and Pearl rivers. For 2050, six scenarios were explored: Baseline SSP5-RCP8.5 (Shared Socio-economic Pathway 5), and alternative scenarios SE (improved sewage treatment), AG (improved nutrient use efficiencies in agriculture), AG+SE (a combination of AG and SE), AG+SE+SFC (sustainable food consumption in addition to AG+SE), AG+SE+SFC+CLI (climate mitigation in addition to AG+SE+SFC).

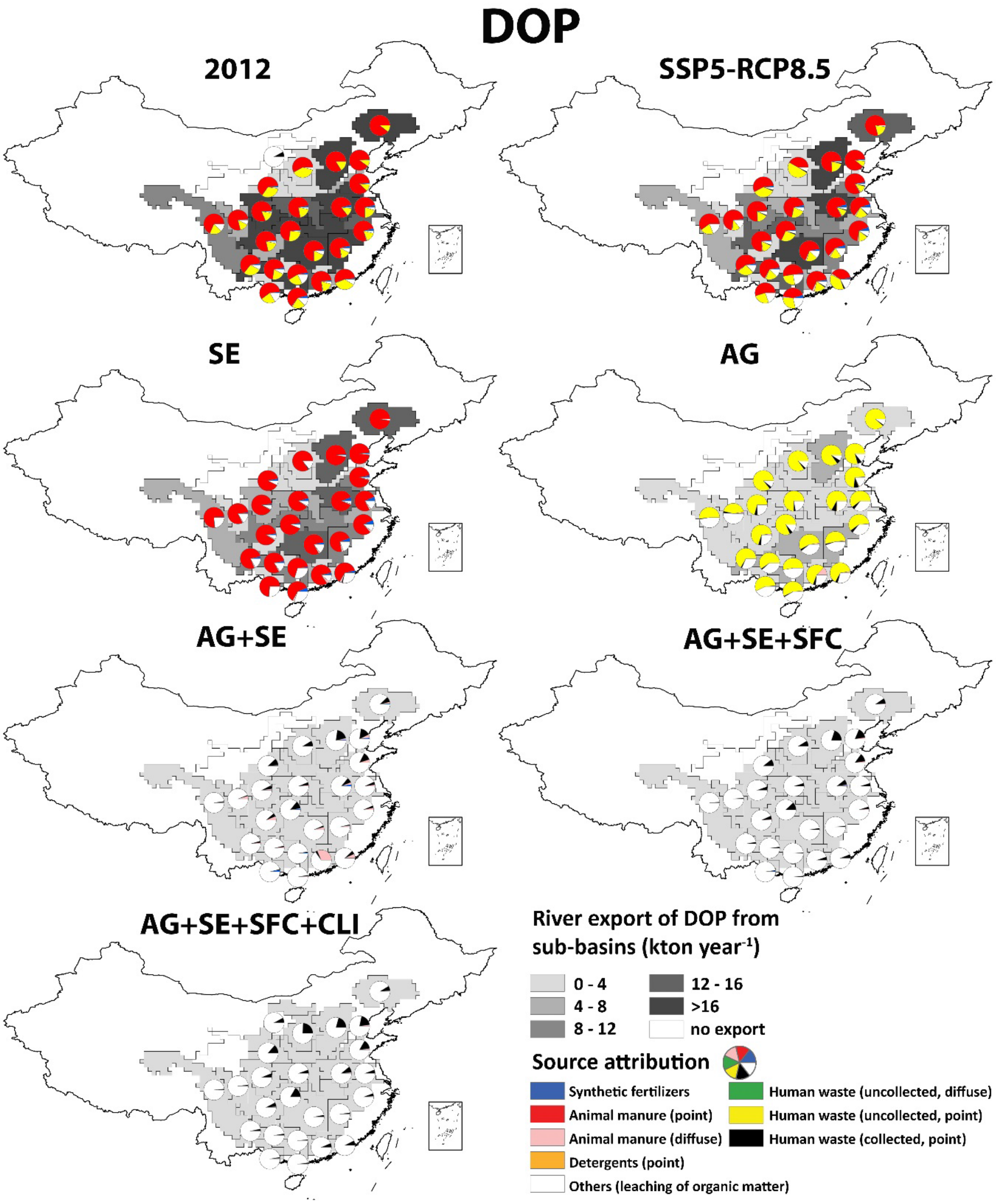

Supplementary Figure 12 River export of dissolved organic phosphorus (DOP) from subbasins of six large Chinese rivers by source in 2012 and 2050 (kton year^{-1}). The six large rivers are: Liao, Hai, Yellow, Huai, Yangtze and Pearl rivers. For 2050, six scenarios were explored: Baseline SSP5-RCP8.5 (Shared Socio-economic Pathway 5), and alternative scenarios SE (improved sewage treatment), AG (improved nutrient use efficiencies in agriculture), AG+SE (a combination of AG and SE), AG+SE+SFC (sustainable food consumption in addition to AG+SE), AG+SE+SFC+CLI (climate mitigation in addition to AG+SE+SFC).

Supplementary Table 8 The contributions of the six scenarios to the achievement of seven relevant SDGs. SSP5-RCP8.5 (a combination of Shared Socio-economic Pathway 5 and Representative Concertation Pathway 8.5) is used as the baseline for the assessment of contributions to meet SDGs by the alternative scenarios: SE (improved sewage treatment), AG (improved nutrient use efficiencies in agriculture), AG+SE (combination of AG and SE), AG+SE+SFC (sustainable food consumption in addition to AG+SE), AG+SE+SFC+CLI (climate mitigation in addition to AG+SE+SFC). In this table we explain how each alternative scenario may contribute to meeting the relevant SDGs. n.a. means that the scenario does not include potential improvements that may contribute to meet the SDGs.

	**SDG6 ***	**SDG14 ***	**SDG2 ***	**SDG11 ***	**SDG12 ***	**SDG13 ***
**SSP5-RCP8.5**						
**SE**	• lower nutrient losses to water systems because of sewage connection and treatment	Same as SE for SDG 6	n.a.	improved sewage connection and treatment	n.a.	n.a.
**AG**	• lower nutrient losses to water systems because of improved nutrient use efficiency in agriculture	Same as AG for SDG 6	• improved nutrient use efficiency in agriculture	n.a.	• improved nutrient use efficiency in agriculture	n.a.
**AG+SE**	A combination of AG+SE	Same as AG+SE for SDG 6	A combination of AG+SE	A combination of AG+SE	A combination of AG+SE	n.a.
**AG+SE+SFC**	In addition to AG+SE: • lower nutrient losses to water systems because of healthy and efficient food consumption	Same as AG+SE+SFC for SDG 6	In addition to AG+SE: • healthy and efficient food consumption	In addition to AG+SE: • healthy and efficient food consumption	In addition to AG+SE: • healthy and efficient food consumption	n.a.
**AG+SE+SFC+CLI**	In addition to AG+SE+SFC: • lower nutrient losses from land to water systems because of climate mitigation	Same as AG+SE+SFC+CLI for SDG 6	Same as AG+SE+SFC	• Same as AG+SE+SFC	• Same as AG+SE+SFC	• climate mitigation

*The six SDGs include: SDG 6 – “Ensure access to water and sanitation for all”, SDG 14 – “Conserve and sustainably use the oceans, seas and marine resources”, SDG 2 – “Zero hunger”, SDG 11 – “Make cities inclusive, safe, resilient and sustainable”, SDG 12 – “Ensure sustainable consumption and production patterns”, and SDG 13 – “Take urgent action to combat climate change and its impacts”.

Appendix D. Water quality standards in China and in other world regions

Supplementary Table 9 Water quality standards for nitrogen and phosphorus concentrations in rivers for different world regions. The sources of the standards are summarized in Supplementary Table 10.

Purposes of the surface water in rivers	Nutrient form	China	WHO	EU	US	Japan	Australia & New Zeeland	South Africa
**Drinking water**	Nitrate (NO_3^-)	10 (as NO_3^--N)	50	50	10 (as NO_3^--N)	10 (as NO_3^--N)	50	0-6 (as NO_3^--N)
	Nitrite (NO_2^-)	1	3	0.2	1 (as NO_2^--N)	0.04	3	0-6 (as NO_2^--N)
	Ammonium (NH_4^+)	0.5 (as NH_4^+-N)	1.5 (odour)<br>35 (taste)	0.5	-	-	0.5	0-1
	Ammonia (NH_3)	1 (as NH_3-N)	-	-	-	-	-	-
	Total Nitrogen	-	-	1-3 (excluding NO_3^-)	-	-	-	-
	Total Phosphorus	0.2	-	0.4-0.7 (P_2O_5)	-	-	-	-
**Bathing water**	Nitrate (NO_3^-)	-	-	[a]	-	-	10 (as NO_3^--N)	-
	Nitrite (NO_2^-)	-	-	-	-	-	0.1 (as NO_2^--N)	-
	Ammonia (NH_3)	1 (as NH_3-N)	-	-	-	-	-	-
	Total Phosphorus	0.2	-	-	-	-	-	-
**Aquaculture**	Nitrite (NO_2^-)	-	-	0.01[b];0.03[c]	-	-	-	-
	Ammonium (NH_4^+)	-	-	0.04-1[b];0.2-1[c]	-	-	-	-
	Ammonia (NH_3)	1 (as NH_3-N)	-	-	-	-	-	-
	Total Nitrogen	-	-	-	-	0.2-1	-	-
	Total Phosphorus	0.2	-	0.2[b];0.4[c]	-	0.01-0.1	-	-
**Irrigation**	Ammonium (NH_4^+)	-	-	2-20 (as NH_4^+-N)	-	-	-	-
	Ammonia (NH_3)	2 (as NH_3-N)	-	-	-	-	-	-
	Total Nitrogen	-	-	-	-	1	5 (long term)[d]<br>25-125 (short term)[e]	-
	Total Phosphorus	0.4	-	25	-	0.1	0.05 (long term)[d]<br>0.8-12 (short term)[e]	-
**Livestock watering**	Nitrate (NO_3^-)	-	-	-	-	-	400	[f]
	Nitrite (NO_2^-)	-	-	-	-	-	30	[f]
	Ammonia (NH_3)	2 (as NH_3-N)	-	-	-	-	-	-

[a] "The concentrations of nitrates, ammonia and phosphates must be checked by the competent authorities when there is a tendency towards the eutrophication of the water"; no specific quantitative standards are given
[b] Salmonid waters
[c] Cyprinid waters
[d] Long term (up to 100 years); the standard for TP is used 'to minimize bio-clogging of irrigation equipment only'.
[e] Short term (up to 20 years)
[f] Specific for animal species

Supplementary Table 10 Sources of the water quality standards in Supplementary Table 9.

Purposes of the surface water in rivers	Nutrient form	China	WHO	EU	US	Japan	Australia & New Zeeland	South Africa
**Drinking water**	Nitrate (NO_3^-)	(MHPRC and SAC, 2006)	(WHO, 2017)	(2009/54/EC, 2009)	(GAO, 2009)	(ME, 1976)	(NHMRC, 2011)	(DWAF, 1996a)
	Nitrite (NO_2^-)	(MHPRC and SAC, 2006)	(WHO, 2017)	(2009/54/EC, 2009)	(GAO, 2009)	(ME, 1976)	(NHMRC, 2011)	(DWAF, 1996a)
	Ammonium (NH_4^+)	(MHPRC and SAC, 2006)	(WHO, 2017)	(2009/54/EC, 2009)	-	-	(NHMRC, 2011)	(DWAF, 1996a)
	Ammonia (NH_3)	(MHPRC and SAC, 2006)	-	-	-	-	-	-
	Total Nitrogen	-	-	(2009/54/EC, 2009)	-	-	-	-
	Total Phosphorus	(MEP, 2002)	-	(2009/54/EC, 2009)	-	-	-	-
**Bathing water**	Nitrate (NO_3^-)	-	-	(2000/60/EC, 2000)	-	-	(Anzecc, 2000)	-
	Nitrite (NO_2^-)	-	-	-	-	-	(Anzecc, 2000)	-
	Ammonia (NH_3)	(MEP, 2002)	-	-	-	-	-	-
	Total Phosphorus	(MEP, 2002)	-	-	-	-	-	-
**Aquaculture**	Nitrite (NO_2^-)	-	-	(2006/44/EC, 2006)	-	-	-	-
	Ammonium (NH_4^+)	-	-	(2006/44/EC, 2006)	-	-	-	-
	Ammonia (NH_3)	(MEP, 2002)	-	-	-	-	-	-
	Total Nitrogen	-	-	-	-	(ME, 1976)	-	-
	Total Phosphorus	(MEP, 2002)	-	(2006/44/EC, 2006)	-	(ME, 1976)	-	-
**Irrigation**	Ammonium (NH_4^+)	-	-	(Aquarec, 2006)	-	-	-	-
	Ammonia (NH_3)	(MEP, 2002)	-	-	-	-	-	-
	Total Nitrogen	-	-	-	-	(ME, 1976)	(Anzecc, 2000)	-
	Total Phosphorus	(MEP, 2002)	-	(Aquarec, 2006)	-	(ME, 1976)	(Anzecc, 2000)	-
**Livestock watering**	Nitrate (NO_3^-)	-	-	-	-	-	(Anzecc, 2000)	(DWAF, 1996b)
	Nitrite (NO_2^-)	-	-	-	-	-	(Anzecc, 2000)	(DWAF, 1996b)
	Ammonia (NH_3)	(MEP, 2002)	-	-	-	-	-	-

Appendix E. Sensitivity analysis

Supplementary Table 11 The setup of the sensitivity analysis for the AG+SE+SFC+CLI scenario implemented in the MARINA 2.0 (Model to Assess River Inputs of Nutrients to seAs) model. The MARINA 2.0 model quantifies nutrient inputs to rivers, and river export of nutrients to seas. In total 12 model inputs are affected by the assumptions in the AG+SE+SFC+CLI scenario. This includes model inputs for sewage systems (no. 1-2), agriculture (no. 3-10), mitigation of atmospheric nitrogen deposition (no. 11), and climate mitigation actions (no. 12).

Model input		Description	Unit	Change relative to AG+SE+SFC+CLI in 2050	
1	$Pop_{con.j}$	population with sewage connection in sub-basin j	people	-10%	+10%
2	$hr_{frem.E.j}$	removal fractions of nutrient element (E) during treatment in sewage systems in sub-basin j	0-1	-10%	+10%
3	$WSdif_{E.fe.j}$	inputs of nutrient element (E) in synthetic fertilizers to agricultural land in sub-basin j	kg year^{-1}	-10%	+10%
4	$fr_{ag.straw.j}$	the fraction of straw that is recycled/applied in agricultural land in sub-basin j	0-1	-10%	+10%
5	$Eexc_{ma.j}$	nutrient element (E) excretion by animals in sub-basin j	kg year^{-1}	-10%	+10%
6	$Gloss_{ma.NH3.j}$	gaseous losses (NH_3 emission) during manure storage in sub-basin j	kg year^{-1}	-10%	+10%
7	$Gloss_{ma.N2O.j}$	gaseous losses (N_2O emission) during manure storage in sub-basin j	kg year^{-1}	-10%	+10%
8	$WSdif_{E.ma.j}$	inputs of nutrient element (E) in animal manure to agricultural land in sub-basin j	kg year^{-1}	-10%	+10%
9	$EXP_{ma.j}$	export of excessive manure to other regions in sub-basin j	kg year^{-1}	-10%	+10%
10	$IM_{food.feed.j}$	import of food and animal feeds in sub-basin j	kg year^{-1}	-10%	+10%
11	$WSdif_{N.dep.j}$	atmospheric nitrogen deposition in sub-basin j	kg year^{-1}	-10%	+10%
12	$Q_{nat.j}$	natural river discharge at the outlet of sub-basin j before water is removed for consumption	km^3 year^{-1}	-10%	+10%

E: nutrient element (nitrogen, phosphorus) j: sub-basin

Supplementary Table 12 Results of the sensitivity analysis for 26 subbasins: changes in model outputs as a result of +10% and -10% change in model inputs, relative to the model run for the AG+SE+SFC+CLI scenario in 2050. Model outputs are annual total dissolved nitrogen (TDN) to rivers. Numbers in the column of “total” refer to the changes in total TDN inputs to rivers (sum for 26 subbasins). Numbers in the column of “max” and “min” refer to the maximum and minimum changes in TDN inputs to rivers for individual subbasins. See Supplementary Table 11 for the setup of the sensitivity analysis and descriptions of the selected model inputs.

Changed model inputs		Changes in model outputs: TDN inputs to rivers (% change)					
		-10% change in model inputs			+10% change in model inputs		
		total	max	min	total	max	min
1	$Pop_{con.j}$	4%	16%	1%	n.a.	n.a.	n.a.
2	$hr_{frem.E.j}$	6%	25%	2%	-6%	-2%	-26%
3	$WSdif_{E.fe.j}$	-2%	0%	-7%	2%	7%	0%
4	$fr_{ag.straw.j}$	2%	3%	0%	-2%	0%	-3%
5	$Eexc_{ma.j}$	-1%	0%	-1%	1%	1%	0%
6	$Gloss_{ma.NH3.j}$	0%	0%	0%	0%	0%	0%
7	$Gloss_{ma.N2O.j}$	0%	0%	0%	0%	0%	0%
8	$WSdif_{E.ma.j}$	14%	41%	4%	n.a.	n.a.	n.a.
9	$EXP_{ma.j}$	0%	6%	0%	n.a.	n.a.	n.a.
10	$IM_{food.feed.j}$	-5%	-1%	-8%	5%	8%	1%
11	$WSdif_{N.dep.j}$	0%	0%	0%	0%	0%	0%
12	$Q_{nat.j}$	-8%	-3%	-10%	8%	10%	3%

Supplementary Table 13 Results of the sensitivity analysis for 26 subbasins: changes in model outputs as a result of +10% and -10% change in model inputs, relative to the model run for the AG+SE+SFC+CLI scenario in 2050. Model outputs are annual total dissolved phosphorus (TDP) to rivers. Numbers in the column of “total” refer to the changes in total TDP inputs to rivers (sum for 26 subbasins). Numbers in the column of “max” and “min” refer to the maximum and minimum changes in TDP inputs to rivers for individual subbasins. See Supplementary Table 11 for the setup of the sensitivity analysis and descriptions of the selected model inputs.

Changed model inputs		Changes in model outputs: TDP inputs to rivers (% change)					
		-10% change in model inputs			+10% change in model inputs		
		total	max	min	total	max	min
1	$Pop_{con.j}$	54%	71%	21%	n.a.	n.a.	n.a.
2	$hr_{frem.E.j}$	68%	87%	37%	-68%	-37%	-87%
3	$WSdif_{E.fe.j}$	-1%	0%	-6%	1%	16%	0%
4	$fr_{ag.straw.j}$	2%	5%	0%	-2%	0%	-5%
5	$Eexc_{ma.j}$	0%	0%	0%	0%	0%	0%
6	$Gloss_{ma.NH3.j}$	0%	0%	0%	0%	0%	0%
7	$Gloss_{ma.N2O.j}$	0%	0%	0%	0%	0%	0%
8	$WSdif_{E.ma.j}$	109%	267%	22%	n.a.	n.a.	n.a.
9	$EXP_{ma.j}$	0%	7%	0%	n.a.	n.a.	n.a.
10	$IM_{food.feed.j}$	0%	0%	-1%	0%	1%	0%
11	$WSdif_{N.dep.j}$	0%	0%	0%	0%	0%	0%
12	$Q_{nat.j}$	-3%	0%	-7%	3%	7%	0%

Supplementary Table 14 Results of the sensitivity analysis for 26 subbasins: changes in model outputs as a result of +10% and -10% change in model inputs, relative to the model run for the AG+SE+SFC+CLI scenario in 2050. Model outputs are annual river export of total dissolved nitrogen (TDN) to sea. Numbers in the column of "total" refer to the changes in total river export of TDN to sea (sum for 26 subbasins). Numbers in the column of "max" and "min" refer to the maximum and minimum changes in river export of TDN to sea for individual subbasins. See Supplementary Table 11 for the setup of the sensitivity analysis and descriptions of the selected model inputs.

Changed model inputs		Changes in model outputs: river export of TDN to sea (% change)					
		-10% change in model inputs			+10% change in model inputs		
		total	max	min	total	max	min
1	$Pop_{con.j}$	7%	36%	0%	n.a.	n.a.	n.a.
2	$hr_{frem.E.j}$	7%	28%	0%	-7%	0%	-29%
3	$WSdif_{E.fe.j}$	-2%	0%	-6%	2%	6%	0%
4	$fr_{ag.straw.j}$	1%	2%	0%	-1%	0%	-2%
5	$Eexc_{ma.j}$	-1%	0%	-1%	1%	1%	0%
6	$Gloss_{ma.NH3.j}$	0%	0%	0%	0%	0%	0%
7	$Gloss_{ma.N2O.j}$	0%	0%	0%	0%	0%	0%
8	$WSdif_{E.ma.j}$	21%	79%	0%	n.a.	n.a.	n.a.
9	$EXP_{ma.j}$	0%	5%	0%	n.a.	n.a.	n.a.
10	$IM_{food.feed.j}$	-4%	0%	-7%	4%	7%	0%
11	$WSdif_{N.dep.j}$	0%	0%	0%	0%	0%	0%
12	$Q_{nat.j}$	-9%	0%	-20%	9%	19%	0%

Supplementary Table 15 Results of the sensitivity analysis for 26 subbasins: changes in model outputs as a result of +10% and -10% change in model inputs, relative to the model run for the AG+SE+SFC+CLI scenario in 2050. Model outputs are annual river export of total dissolved phosphorus (TDP) to sea. Numbers in the column of "total" refer to the changes in total river export of TDP to sea (sum for 26 subbasins). Numbers in the column of "max" and "min" refer to the maximum and minimum changes in river export of TDP to sea for individual subbasins. See Supplementary Table 11 for the setup of the sensitivity analysis and descriptions of the selected model inputs.

Changed model inputs		Changes in model outputs: river export of TDP to sea (% change)					
		-10% change in model inputs			+10% change in model inputs		
		total	max	min	total	max	min
1	$Pop_{con.j}$	74%	502%	0%	n.a.	n.a.	n.a.
2	$hr_{frem.E.j}$	48%	81%	0%	-48%	0%	-81%
3	$WSdif_{E.fe.j}$	-1%	0%	-4%	1%	12%	0%
4	$fr_{ag.straw.j}$	1%	4%	0%	-1%	0%	-4%
5	$Eexc_{ma.j}$	0%	0%	0%	0%	0%	0%
6	$Gloss_{ma.NH3.j}$	0%	0%	0%	0%	0%	0%
7	$Gloss_{ma.N2O.j}$	0%	0%	0%	0%	0%	0%
8	$WSdif_{E.ma.j}$	143%	394%	0%	n.a.	n.a.	n.a.
9	$EXP_{ma.j}$	1%	7%	0%	n.a.	n.a.	n.a.
10	$IM_{food.feed.j}$	0%	0%	0%	0%	0%	0%
11	$WSdif_{N.dep.j}$	0%	0%	0%	0%	0%	0%
12	$Q_{nat.j}$	-7%	0%	-61%	7%	22%	0%

Supplementary Table 16 Results of the sensitivity analysis with -10% changes in model inputs: number of subbasins for which model outputs change by <5%, 5-10%, >10%. Model outputs are annual total dissolved nitrogen (TDN) inputs to rivers. See Supplementary Table 11 for the setup of the sensitivity analysis and descriptions of the selected model inputs.

Changes in model outputs (%)	Number of the subbasins with the change in model outputs for TDN inputs to rivers											
	$Pop_{con.j}$	$hr_{frem.E.j}$	$WSdif_{E.fe.j}$	$fr_{ag.straw.j}$	$Eexc_{ma.j}$	$Gloss_{ma.NH3.j}$	$Gloss_{ma.N2O.j}$	$WSdif_{E.ma.j}$	$EXP_{ma.j}$	$IM_{food.feed.j}$	$WSdif_{N.dep.j}$	$Q_{nat.j}$
<5	15	10	24	26	26	26	26	4	25	19	26	1
5-10	8	11	2	0	0	0	0	6	1	7	0	25
>10	3	5	0	0	0	0	0	16	0	0	0	0

Supplementary Table 17 Results of the sensitivity analysis with +10% changes in model inputs: number of subbasins for which model outputs change by <5%, 5-10%, >10%. Model outputs are annual total dissolved nitrogen (TDN) inputs to rivers. See Supplementary Table 11 for the setup of the sensitivity analysis and descriptions of the selected model inputs.

Changes in model outputs (%)	Number of the subbasins with the change in model outputs for TDN inputs to rivers											
	$Pop_{con.j}$	$hr_{frem.E.j}$	$WSdif_{E.fe.j}$	$fr_{ag.straw.j}$	$Eexc_{ma.j}$	$Gloss_{ma.NH3.j}$	$Gloss_{ma.N2O.j}$	$WSdif_{E.ma.j}$	$EXP_{ma.j}$	$IM_{food.feed.j}$	$WSdif_{N.dep.j}$	$Q_{nat.j}$
<5	n.a.	10	24	26	26	26	26	n.a.	n.a.	19	26	1
5-10	n.a.	10	2	0	0	0	0	n.a.	n.a.	7	0	25
>10	n.a.	6	0	0	0	0	0	n.a.	n.a.	0	0	0

Supplementary Table 18 Results of the sensitivity analysis with -10% changes in model inputs: number of subbasins for which model outputs change by <5%, 5-10%, >10%. Model outputs are annual total dissolved phosphorus (TDP) inputs to rivers. See Supplementary Table 11 for the setup of the sensitivity analysis and descriptions of the selected model inputs.

	Number of the subbasins with the change in model outputs for TDP inputs to rivers											
Changes in model outputs (%)	$Pop_{con.j}$	$hr_{frem.E.j}$	$WSdif_{E.fe.j}$	$fr_{ag.straw.j}$	$Eexc_{ma.j}$	$Gloss_{ma.NH3.j}$	$Gloss_{ma.N2O.j}$	$WSdif_{E.ma.j}$	$EXP_{ma.j}$	$IM_{food.feed.j}$	$WSdif_{N.dep.j}$	$Q_{nat.j}$
<5	0	0	25	25	26	26	26	0	25	26	26	19
5-10	0	0	1	1	0	0	0	0	1	0	0	7
>10	26	26	0	0	0	0	0	26	0	0	0	0

Supplementary Table 19 Results of the sensitivity analysis with +10% changes in model inputs: number of subbasins for which model outputs change by <5%, 5-10%, >10%. Model outputs are annual total dissolved phosphorus (TDP) inputs to rivers. See Supplementary Table 11 for the setup of the sensitivity analysis and descriptions of the selected model inputs.

	Number of the subbasins with the change in model outputs for TDP inputs to rivers											
Changes in model outputs (%)	$Pop_{con.j}$	$hr_{frem.E.j}$	$WSdif_{E.fe.j}$	$fr_{ag.straw.j}$	$Eexc_{ma.j}$	$Gloss_{ma.NH3.j}$	$Gloss_{ma.N2O.j}$	$WSdif_{E.ma.j}$	$EXP_{ma.j}$	$IM_{food.feed.j}$	$WSdif_{N.dep.j}$	$Q_{nat.j}$
<5	n.a.	0	24	25	26	26	26	n.a.	n.a.	26	26	20
5-10	n.a.	0	0	1	0	0	0	n.a.	n.a.	0	0	6
>10	n.a.	26	2	0	0	0	0	n.a.	n.a.	0	0	0

Supplementary Table 20 Results of the sensitivity analysis with -10% changes in model inputs: number of subbasins for which model outputs change by <5%, 5-10%, >10%. Model outputs are annual river export of total dissolved nitrogen (TDN) to seas. See Supplementary Table 11 for the setup of the sensitivity analysis and descriptions of the selected model inputs.

Changes in model outputs (%)	Number of the subbasins with the change in model outputs for river export of TDN											
	$Pop_{con.j}$	$hr_{frem.E.j}$	$WSdif_{E.fe.j}$	$fr_{ag.straw.j}$	$Eexc_{ma.j}$	$Gloss_{ma.NH3.j}$	$Gloss_{ma.N2O.j}$	$WSdif_{E.ma.j}$	$EXP_{ma.j}$	$IM_{food.feed.j}$	$WSdif_{N.dep.j}$	$Q_{nat.j}$
<5	9	9	25	26	26	26	26	1	25	22	26	3
5-10	9	9	1	0	0	0	0	4	1	4	0	15
>10	8	8	0	0	0	0	0	21	0	0	0	8

Supplementary Table 21 Results of the sensitivity analysis with +10% changes in model inputs: number of subbasins for which model outputs change by <5%, 5-10%, >10%. Model outputs are annual river export of total dissolved nitrogen (TDN) to seas. See Supplementary Table 11 for the setup of the sensitivity analysis and descriptions of the selected model inputs.

Changes in model outputs (%)	Number of the subbasins with the change in model outputs for river export of TDN											
	$Pop_{con.j}$	$hr_{frem.E.j}$	$WSdif_{E.fe.j}$	$fr_{ag.straw.j}$	$Eexc_{ma.j}$	$Gloss_{ma.NH3.j}$	$Gloss_{ma.N2O.j}$	$WSdif_{E.ma.j}$	$EXP_{ma.j}$	$IM_{food.feed.j}$	$WSdif_{N.dep.j}$	$Q_{nat.j}$
<5	n.a.	9	25	26	26	26	26	n.a.	n.a.	22	26	3
5-10	n.a.	8	1	0	0	0	0	n.a.	n.a.	4	0	16
>10	n.a.	9	0	0	0	0	0	n.a.	n.a.	0	0	7

Supplementary Table 22 Results of the sensitivity analysis with -10% changes in model inputs: number of subbasins for which model outputs change by <5%, 5-10%, >10%. Model outputs are annual river export of total dissolved phosphorus (TDP) to seas. See Supplementary Table 11 for the setup of the sensitivity analysis and descriptions of the selected model inputs.

	Number of the subbasins with the change in model outputs for river export of TDP											
Changes in model outputs (%)	$Pop_{con.j}$	$hr_{frem.E.j}$	$WSdif_{E.fe.j}$	$fr_{ag.straw.j}$	$Eexc_{ma.j}$	$Gloss_{ma.NH3.j}$	$Gloss_{ma.N2O.j}$	$WSdif_{E.ma.j}$	$EXP_{ma.j}$	$IM_{food.feed.j}$	$WSdif_{N.dep.j}$	$Q_{nat.j}$
<5	1	1	26	26	26	26	26	1	25	26	26	7
5-10	0	1	0	0	0	0	0	0	1	0	0	12
>10	25	24	0	0	0	0	0	25	0	0	0	7

Supplementary Table 23 Results of the sensitivity analysis with +10% changes in model inputs: number of subbasins for which model outputs change by <5%, 5-10%, >10%. Model outputs are annual river export of total dissolved phosphorus (TDP) to seas. See Supplementary Table 11 for the setup of the sensitivity analysis and descriptions of the selected model inputs.

	Number of the subbasins with the change in model outputs for river export of TDP											
Changes in model outputs (%)	$Pop_{con.j}$	$hr_{frem.E.j}$	$WSdif_{E.fe.j}$	$fr_{ag.straw.j}$	$Eexc_{ma.j}$	$Gloss_{ma.NH3.j}$	$Gloss_{ma.N2O.j}$	$WSdif_{E.ma.j}$	$EXP_{ma.j}$	$IM_{food.feed.j}$	$WSdif_{N.dep.j}$	$Q_{nat.j}$
<5	n.a.	1	24	26	26	26	26	n.a.	n.a.	26	26	9
5-10	n.a.	1	1	0	0	0	0	n.a.	n.a.	0	0	12
>10	n.a.	24	1	0	0	0	0	n.a.	n.a.	0	0	5

Supplementary References

1. 2000/60/EC, D. 2000. Directive 2000/60/EC of the European Parliament and of the Council establishing a framework for the Community action in the field of water policy. *Official Journal of the European union,* OJ L 327.
2. 2006/44/EC, D. 2006. Directive 2006/44/EC of the European Parliament and of the Council of 6 September 2006 on the quality of fresh waters needing protection or improvement in order to support fish life (codified version). *Official Journal of the European union,* L 264/20.
3. 2009/54/EC, D. 2009. Directive 2009/54/EC of the European Parliament and of the Council of 18 June 2009 on the exploitation and marketing of natural mineral waters. *Official Journal of the European union,* L 164/45.
4. ABBASI, H. N., LU, X., XU, F. & XIE, J. 2016. Wastewater treatment strategies in china: An overview. *Sci. Lett,* 4**,** 15-25.
5. ALAM, M. J. & DUTTA, D. 2013. Predicting climate change impact on nutrient pollution in waterways: a case study in the upper catchment of the Latrobe River, Australia. *Ecohydrology,* 6**,** 73-82.
6. ANZECC, A. 2000. Australian and New Zealand guidelines for fresh and marine water quality. *Australian and New Zealand Environment and Conservation Council and Agriculture and Resource Management Council of Australia and New Zealand, Canberra***,** 1-103.
7. AQUAREC 2006. Guidelines for quality standards for water reuse in Europe. EVK1-CT-2002-00130, Work package 2, Deliverable D15, Edited by Miquel Salgot and Esther Huertas, Unievrsity of Barcelona.
8. BLANKE, A., ROZELLE, S., LOHMAR, B., WANG, J. & HUANG, J. 2007. Water saving technology and saving water in China. *Agricultural water management,* 87**,** 139-150.
9. BOYD, P. W. 2011. Beyond ocean acidification. *Nature Geoscience,* 4**,** 273.
10. BROOKS, N. 2015. Impending Water Crisis in China. Staff writing for the Global Water Crisis project at the Arlington Institute.
11. CHEN, N., HONG, H., HUANG, Q. & WU, J. 2011. Atmospheric nitrogen deposition and its long-term dynamics in a southeast China coastal area. *Journal of environmental management,* 92**,** 1663-1667.
12. CHIU, A. S. 2011. Sustainable consumption and production policy options in Asia and the Pacific. *Procedia-Social and Behavioral Sciences,* 25**,** 413-418.
13. DANKELMAN, I. 2012. On the road to sustainable development: Promoting gender equality and adressing climate change. *Powerful Synergies***,** 25.
14. DÖLL, P. & LEHNER, B. 2002. Validation of a new global 30-min drainage direction map. *Journal of Hydrology,* 258**,** 214-231.
15. DWAF 1996a. South African Water Quality Guidelines (second edition). Volume 1: Domestic Use. *In:* FORESTRY, D. O. W. A. A. (ed.).
16. DWAF 1996b. South African Water Quality Guidelines (second edition). Volume 5: Livestock Watering. *In:* FORESTRY, D. O. W. A. A. (ed.).
17. GAFFNEY, O. 2014. Sustainable Development Goals: Improving human and planetary wellbeing. *Glob Change,* 82**,** 20-23.
18. GAO 2009. Bottled water: FDA Safety and Consumer Protections are often less stringent than comparable EPA protections for tap water. Washington, D.C., United States: Government Accountability Office.
19. GLIBERT, P. M. 2013. Harmful Algal Blooms in Asia: an insidious and escalating water pollution phenomenon with effects on ecological and human health. *ASIANetwork Exchange,* 21.
20. GRIGGS, D., NILSSON, M., STEVANCE, A. & MCCOLLUM, D. 2017. *A guide to SDG interactions: from science to implementation*, International Council for Science, Paris.
21. GROSS, C. & HAGY III, J. D. 2017. Attributes of successful actions to restore lakes and estuaries degraded by nutrient pollution. *Journal of environmental management,* 187**,** 122-136.
22. GU, B., GE, Y., REN, Y., XU, B., LUO, W., JIANG, H., GU, B. & CHANG, J. 2012a. Atmospheric reactive nitrogen in China: sources, recent trends, and damage costs. *Environmental science & technology,* 46**,** 9420-9427.
23. GU, B. J., GE, Y., REN, Y., XU, B., LUO, W. D., JIANG, H., GU, B. H. & CHANG, J. 2012b. Atmospheric Reactive Nitrogen in China: Sources, Recent Trends, and Damage Costs. *Environmental Science & Technology,* 46**,** 9420-9427.
24. HAN, D., CURRELL, M. J. & CAO, G. 2016. Deep challenges for China's war on water pollution. *Environmental Pollution,* 218**,** 1222-1233.
25. KATUKIZA, A., RONTELTAP, M., NIWAGABA, C., FOPPEN, J., KANSIIME, F. & LENS, P. 2012. Sustainable sanitation technology options for urban slums. *Biotechnology advances,* 30**,** 964-978.
26. KELLY, R. P., FOLEY, M., FISHER, W., FEELY, R., HALPERN, B., WALDBUSSER, G. & CALDWELL, M. 2011. Mitigating local causes of ocean acidification with existing laws. *Science,* 332**,** 1036-1037.
27. KHOSLA, P., VAN WIJK, C., VERHAGEN, J., FRANCIS, J. & ARCE, M. 2004. *Gender and water*, International Water and Sanitation Centre (IRC) Delft, the Netherlands.
28. LI, Y., ZHANG, W., MA, L., HUANG, G., OENEMA, O., ZHANG, F. & DOU, Z. 2013. An analysis of China's fertilizer policies: Impacts on the industry, food security, and the environment. *Journal of environmental quality,* 42**,** 972-981.
29. LIU, J. & YANG, W. 2012. Water sustainability for China and beyond. *Science,* 337**,** 649-650.
30. LIU, X., ZHANG, Y., HAN, W., TANG, A., SHEN, J., CUI, Z., VITOUSEK, P., ERISMAN, J. W., GOULDING, K. & CHRISTIE, P. 2013. Enhanced nitrogen deposition over China. *Nature,* 494**,** 459-462.
31. LIU, Y., PENG, Z., ZHOU, R., SONG, S., LIU, W., YOU, C.-F., LIN, Y.-P., YU, K., WU, C.-C. & WEI, G. 2014. Acceleration of modern acidification in the South China Sea driven by anthropogenic CO 2. *Scientific reports,* 4**,** 5148.

32. LIU, Y., ZHOU, Y. & WU, W. 2015. Assessing the impact of population, income and technology on energy consumption and industrial pollutant emissions in China. *Applied Energy,* 155**,** 904-917.
33. LU, Y., ZHANG, Y., CAO, X., WANG, C., WANG, Y., ZHANG, M., FERRIER, R. C., JENKINS, A., YUAN, J. & BAILEY, M. J. 2019. Forty years of reform and opening up: China's progress toward a sustainable path. *Science advances,* 5**,** eaau9413.
34. MA, L., BAI, Z., MA, W., GUO, M., JIANG, R., LIU, J., OENEMA, O., VELTHOF, G. L., WHITMORE, A. P., CRAWFORD, J., DOBERMANN, A., SCHWOOB, M. & ZHANG, F. 2019. Exploring Future Food Provision Scenarios for China. *Environmental Science & Technology,* 53**,** 1385-1393.
35. MA, L., WANG, F., ZHANG, W., MA, W., VELTHOF, G., QIN, W., OENEMA, O. & ZHANG, F. 2013. Environmental assessment of management options for nutrient flows in the food chain in China. *Environmental science & technology,* 47**,** 7260-7268.
36. ME 1976. Environmental Quality Standards for Water pollution. *In:* ENVIRONMENT, M. O. T. (ed.). Japan.
37. MEP 2002. Environmental Quality Standard for Surface Water. *In:* CHINA, M. O. E. A. E. O. T. P. S. R. O. (ed.). Beijing, China.
38. MHPRC & SAC 2006. Standards for Drinking Water Quality. *In:* MINISTRY OF HEALTH OF THE PEOPLE'S REPUBLIC OF CHINA, S. A. O. C. (ed.). Beijing, China.
39. MURRAY, A., MEKALA, G. D. & CHEN, X. 2011. Evolving policies and the roles of public and private stakeholders in wastewater and faecal-sludge management in India, China and Ghana. *Water International,* 36**,** 491-504.
40. NDRC-MOHURD 2016. National Urban Sewage Treatment and Recycling Planning in 13th Five-Year (2015-2020). *In:* NATIONAL DEVELOPMENT AND REFORM COMMISSION, M. O. H. A. U.-R. D. O. T. P. S. R. O. C. (ed.).
41. NHMRC, N. 2011. Australian Drinking Water Guidelines Paper 6 National Water Quality Management Strategy. *In:* NATIONAL HEALTH AND MEDICAL RESEARCH COUNCIL, N. R. M. M. C., COMMONWEALTH OF AUSTRALIA (ed.). Canberra.
42. NYSTRÖM, M., NORSTRÖM, A. V., BLENCKNER, T., DE LA TORRE-CASTRO, M., EKLÖF, J. S., FOLKE, C., ÖSTERBLOM, H., STENECK, R. S., THYRESSON, M. & TROELL, M. 2012. Confronting feedbacks of degraded marine ecosystems. *Ecosystems,* 15**,** 695-710.
43. PARTNERSHIP, G. W. 2015. China's water resources management challenge: The 'three red lines'. Global Water Partnership (GWP) Secretariat Stockholm.
44. QIN, W. 2015. *Exploring options for improving water and nitrogen use efficiency in crop production systems.* Wageningen University.
45. RESDC. 2013. Data Center for Resources and Environmental Sciences Chinese Academy of Sciences Available: http://www.resdc.cn [Accessed 02-15 2016].
46. SHEN, D. 2006. Access to water and sanitation in China: history, current situation and challenges. Human Development Report Office (HDRO), United Nations Development Programme ….
47. SIMS, J., MA, L., OENEMA, O., DOU, Z. & ZHANG, F. 2013. Advances and challenges for nutrient management in China in the 21st century. *Journal of environmental quality,* 42**,** 947-950.
48. SMITH, L. E. & SICILIANO, G. 2015. A comprehensive review of constraints to improved management of fertilizers in China and mitigation of diffuse water pollution from agriculture. *Agriculture, Ecosystems & Environment,* 209**,** 15-25.
49. STROKAL, M., KROEZE, C., WANG, M. & MA, L. 2017. Reducing future river export of nutrients to coastal waters of China in optimistic scenarios. *Science of The Total Environment,* 579**,** 517-528.
50. SUN, B., ZHANG, L., YANG, L., ZHANG, F., NORSE, D. & ZHU, Z. 2012. Agricultural non-point source pollution in China: causes and mitigation measures. *Ambio,* 41**,** 370-379.
51. SUTTON, M. A., BLEEKER, A., HOWARD, C., ERISMAN, J., ABROL, Y., BEKUNDA, M., DATTA, A., DAVIDSON, E., DE VRIES, W. & OENEMA, O. 2013. Our nutrient world. The challenge to produce more food & energy with less pollution. Centre for Ecology & Hydrology.
52. SZOGI, A. A., VANOTTI, M. B. & RO, K. S. 2015. Methods for treatment of animal manures to reduce nutrient pollution prior to soil application. *Current Pollution Reports,* 1**,** 47-56.
53. TAO, T. & XIN, K. 2014. Public health: A sustainable plan for China's drinking water. *Nature News,* 511**,** 527.
54. WANG, M., KROEZE, C., STROKAL, M. & MA, L. 2017. Reactive nitrogen losses from China's food system for the shared socioeconomic pathways (SSPs). *Science of The Total Environment,* 605–606**,** 884-893.
55. WANG, M., KROEZE, C., STROKAL, M., VAN VLIET, M. T. H. & MA, L. 2020. Global change can make coastal eutrophication control in China more difficult. *Earth's Future,* n/a**,** e2019EF001280.
56. WANG, M., MA, L., STROKAL, M., CHU, Y. & KROEZE, C. 2018. Exploring nutrient management options to increase nitrogen and phosphorus use efficiencies in food production of China. *Agricultural Systems,* 163**,** 58-72.
57. WANG, M., WEBBER, M., FINLAYSON, B. & BARNETT, J. 2008. Rural industries and water pollution in China. *Journal of Environmental management,* 86**,** 648-659.
58. WHO 2017. Guidelines for drinking-water quality: fourth edition incorporating the first addendum. Geneva: World Health Organization.
59. ZENG, X., CHEN, X. & ZHUANG, J. 2015. The positive relationship between ocean acidification and pollution. *Marine Pollution Bulletin,* 91**,** 14-21.
60. ZHANG, J. J., MAUZERALL, D. L., ZHU, T., LIANG, S., EZZATI, M. & REMAIS, J. 2010. Environmental health in China: challenges to achieving clean air and safe water. *Lancet,* 375**,** 1110.
61. ZHANG, K. 2016. Regime shifts and resilience in China's coastal ecosystems. *Ambio,* 45**,** 89-98.
62. ZHANG, Y., ZHENG, L., LIU, X., JICKELLS, T., CAPE, J. N., GOULDING, K., FANGMEIER, A. & ZHANG, F. 2008. Evidence for organic N deposition and its anthropogenic sources in China. *Atmospheric Environment,* 42**,** 1035-1041.

63. ZHEN, N., FU, B., LU, Y. & WANG, S. 2014. Poverty reduction, environmental protection and ecosystem services: A prospective theory for sustainable development. *Chinese geographical science,* 24**,** 83-92.
64. ZHU, J., HE, N., WANG, Q., YUAN, G., WEN, D., YU, G. & JIA, Y. 2015. The composition, spatial patterns, and influencing factors of atmospheric wet nitrogen deposition in Chinese terrestrial ecosystems. *Science of the Total Environment,* 511**,** 777-785.
